# Food groups and urologic cancers risk: a systematic review and meta-analysis of prospective studies

**DOI:** 10.3389/fnut.2023.1154996

**Published:** 2023-05-17

**Authors:** Jingyi Qi, Peng An, Dekui Jin, Yuting Ji, Sitong Wan, Xu Zhang, Yongting Luo, Junjie Luo, Chengying Zhang

**Affiliations:** ^1^Department of Nutrition and Health, Beijing Advanced Innovation Center for Food Nutrition and Human Health, China Agricultural University, Beijing, China; ^2^Department of General Practice, The Third Medical Center of Chinese PLA General Hospital, Beijing, China

**Keywords:** urologic cancers, foods, prospective cohort, meta-analysis, dose–response

## Abstract

**Background:**

To assess the association between 12 food groups intake and the risk of urologic cancers.

**Methods:**

We scanned PubMed and Web of Science databases up to April 1st, 2023, and 73 publications met the inclusion criteria in the meta-analysis. We used a random effects model to estimate the summary risk ratios (RRs) and 95% confidence intervals (95% CI).

**Results:**

In the linear dose–response meta-analysis, an inverse association was found between each additional daily 100 g of fruits [RR: 0.89, 95%CI = (0.83, 0.97)], 100 g of vegetables [RR: 0.92, 95%CI = (0.85, 0.99)], 12 g of alcohol [RR: 0.91, 95%CI = (0.88, 0.94)] and 1 cup of coffee [RR: 0.95, 95%CI = (0.83, 0.97)] intake and the risk of renal cell carcinoma. Conversely, each additional daily 100 g of red meat intake was positively associated with renal cell carcinoma [RR: 1.41, 95%CI = (1.03, 2.10)]. Inverse associations were observed between each additional daily 50 g of egg [RR: 0.73, 95%CI = (0.62, 0.87)] and each additional daily 1 cup of tea consumption and bladder cancer risk [RR: 0.97, 95%CI = (0.94, 0.99)]. There were no significant associations for nonlinear dose–response relationships between 12 food groups and urological cancers.

**Conclusion:**

Our meta-analysis strengthens the evidence that appropriate intake of specific food groups, such as fruits, vegetables, alcohol, tea, and coffee, is associated with the risk of renal cell carcinoma or bladder cancer. More studies are required to fill the knowledge gap on the links between various food groups and urologic cancers because the evidence was less credible in this meta-analysis.

**Systematic Review Registration:**

This study was registered on PROSPERO (CRD42022340336).

**Graphical Abstract:**
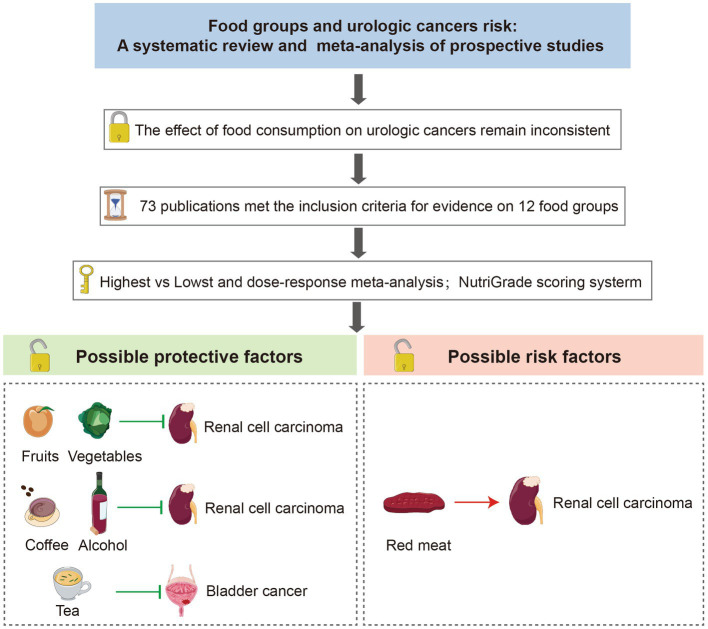


## 1. Introduction

Urologic cancers can occur anywhere in the kidney, bladder, renal pelvis, ureter, and urethra. Notably, renal cell carcinoma (RCC) and bladder cancer (BC) are the most common urinary system tumors in both men and women. RCC and BC incidence have been steadily increasing worldwide over the past decades (1). According to GLOBOCAN data, more than 431,000 new cases of RCC were diagnosed and more than 573,000 new cases of BC were recorded worldwide in 2020 (2). Despite the identification of several modifiable lifestyle risk factors, including excess body weight (3), hypertension (4), smoking (5), and physical inactivity (6), there has been little progress in understanding the origin of urologic cancers.

Efforts to find a connection between diet and urologic cancers have a long history in cancer research. Currently, several studies have demonstrated a strong connection between food and the risk of urologic cancers (7–10). Many dietary factors, such as total fruits, total vegetables, processed meat, and alcohol consumption, are thought to influence urologic cancers risk (11–14). However, insights from epidemical studies on the modifying effects of food consumption on urologic cancers are still controversial (15–18). Therefore, more updated and sufficient evidence is needed to address these long-controversial issues. In this meta-analysis, we aim to investigate the associations of 12 food groups with the risk of urologic cancers, evaluate the food groups’ credibility of meta-evidence on their association with urologic cancers risk, and propose optimally effective strategies for the prevention of urologic cancers.

## 2. Methods

The PROSPERO International Prospective Register of Systematic Reviews has acknowledged the protocol for this meta-analysis (CRD42022340336). This systematic review was developed based on the guidelines of the Preferred Reporting Items for Systematic Reviews and meta-Analyses (PRISMA) (19).

### 2.1. Search strategy and study selection

To investigate the association between specific food group intake and urologic cancer risk, articles published in PubMed and Web of Science before April 1st, 2023 were searched. The search was restricted to the English language. The keywords used in the search strategy are presented as search terms (Supplementary Appendix S1). To discover pertinent research more comprehensively, the electronic search method also looked through all related earlier reviews. The study inclusion criteria were as follows: (1) prospective cohort studies, case cohorts, nested case–control studies; (2) studies reported the association for at least one of the 12 food groups (fruits, vegetables, legumes, egg, dairy, fish, red meat, processed meat, sugar-sweetened beverages (SSB), alcoholic drinks, coffee, and tea) and risk of urologic cancers; (3) the authors reported the risk ratio (RR) estimates or hazard ratios (HRs) with 95% confidence intervals (CIs) or the number of urologic cancers events. Exclusion criteria were (1) studies did not report relevant exposure; (2) studies did not contain cases of urinary system cancer; (3) reviews, meta-analyses, retrospective studies, non-human studies, studies without sufficient data, case–control studies, cross-sectional designs, and interventional studies.

After screening the titles and abstracts, duplicate papers and those that did not fit the criteria for inclusion had been removed. The full-texts of the remaining records were then assessed for eligibility.

### 2.2. Data extraction

Our 2 reviewers (S.W. and X.Z.) independently extracted the information as follows: first author’s name, year of publication, country, cohort name, study duration (years of follow-up), sex, age, cases, sample size, exposure assessment method, outcome, type of food groups, quantity of food intake, risk estimate (most adjusted RRs or HRs with 95% CI), and covariates used for adjustment. When the same study appeared to have multiple publications, we selected the version which contains the largest sample and longest follow-up.

### 2.3. Risk of bias assessment

The Newcastle-Ottawa scale (NOS) was used to assess the methodological quality of prospective cohort studies included (20). It contains 8 categories relating to methodological quality: representativeness of the exposed cohort, selection of non-exposed cohort, ascertainment of exposure, demonstration that the outcome of interest was not present at the start of the study, comparability of the cohorts on the basis of the design or analysis, assessment of outcome, follow-up duration, and adequacy of follow up of cohorts. This scoring system suggests classifying the meta-evidence into three categories: low (0–3 points), moderate (4–6 points), and high (7–9 points).

### 2.4. Statistical analysis

We used the random effects model to calculate the pooled RR and 95% CI, and linear or non-linear dose–response analysis. The HRs reported in the included studies were considered equal to RRs. We carried out the dose–response meta-analysis using the approach suggested by Greenland and Longnecker et al. (21). The distribution of cases, person-years or non-cases, as well as the RRs with 95% CIs, were required for at least three quantitative exposure categories when we applied this method. In dose–response meta-analysis, the lowest intake category from each study was used as the reference, and the other intake categories were compared to the reference. When the exposure category was reported in the closed interval, consumption was considered as the midpoint of the interval. When the exposure category was open-ended, we assumed that its length was the same as the adjacent category.

Restricted cubic splines for each study with more than 3 quantiles of exposure were calculated to explore possible nonlinear associations. We used three fixed knots through the total range of the reported intake at 10, 50, and 90% (22, 23). Units of exposure were defined as follows: total fruits (100 g/day), total vegetables (100 g/day), legumes (100 g/day), egg (50 g/day), dairy (200 g/day), fish (100 g/day), red meat (100 g/day), processed meat (50 g/day), alcohol (12 g/day), coffee (1 cup/day), tea (1 cup/day) and SSB (1 drink/day). When the studies did not specify the quantitative amount or reported food intake as serving size only, we adopted the WCRF 2017 suggested conversions (Supplementary Table S1).

We performed subgroup analyses of potential influencing factors to discern the source of heterogeneity. If there were an adequate number of studies (*n* ≥ 5) available for a particular food group in the meta-analysis, subgroup analyses by geography (US, UK, Asia), sex (Male, Female, Male and female), follow-up duration (mean ≥ 10 years vs. <10 years), no of participants (≥100,000 vs. <100,000). Egger’s linear regression tests and visual inspection of funnel plots were used to evaluate publication bias (24, 25). Furthermore, we conducted sensitivity analysis by omitting one study at a time when significant publication bias (*p* > 0.05) or heterogeneity (I^2^ ≥ 50%) was detected in the results. All statistical analyses in this systemic review were performed with Stata (version 14; Stata Corp). Two-tailed was used in all tests and value of *p* of less than 0.05 was considered to indicate statistical significance.

### 2.5. Quality of meta-evidence

Two independent researchers (J.Q. and D.J.) evaluated the overall quality of the evidence using the NutriGrade scoring system (max 10 points). This tool comprises the following items: (1) risk of bias, study quality, and study limitations; (2) precision; (3) heterogeneity; (4) directness; (5) publication bias; (6) funding bias; (7) effect size; and (8) dose–response (26). Scores between 0 and 3.99, 4–5.99, 6–7.99 were categorized as very low, low, and moderate, and score between 8–10 represents good quality meta-evidence, respectively. Disagreements were settled by conversation until an agreement was achieved.

## 3. Results

### 3.1. Study characteristics

**Figure 1:**
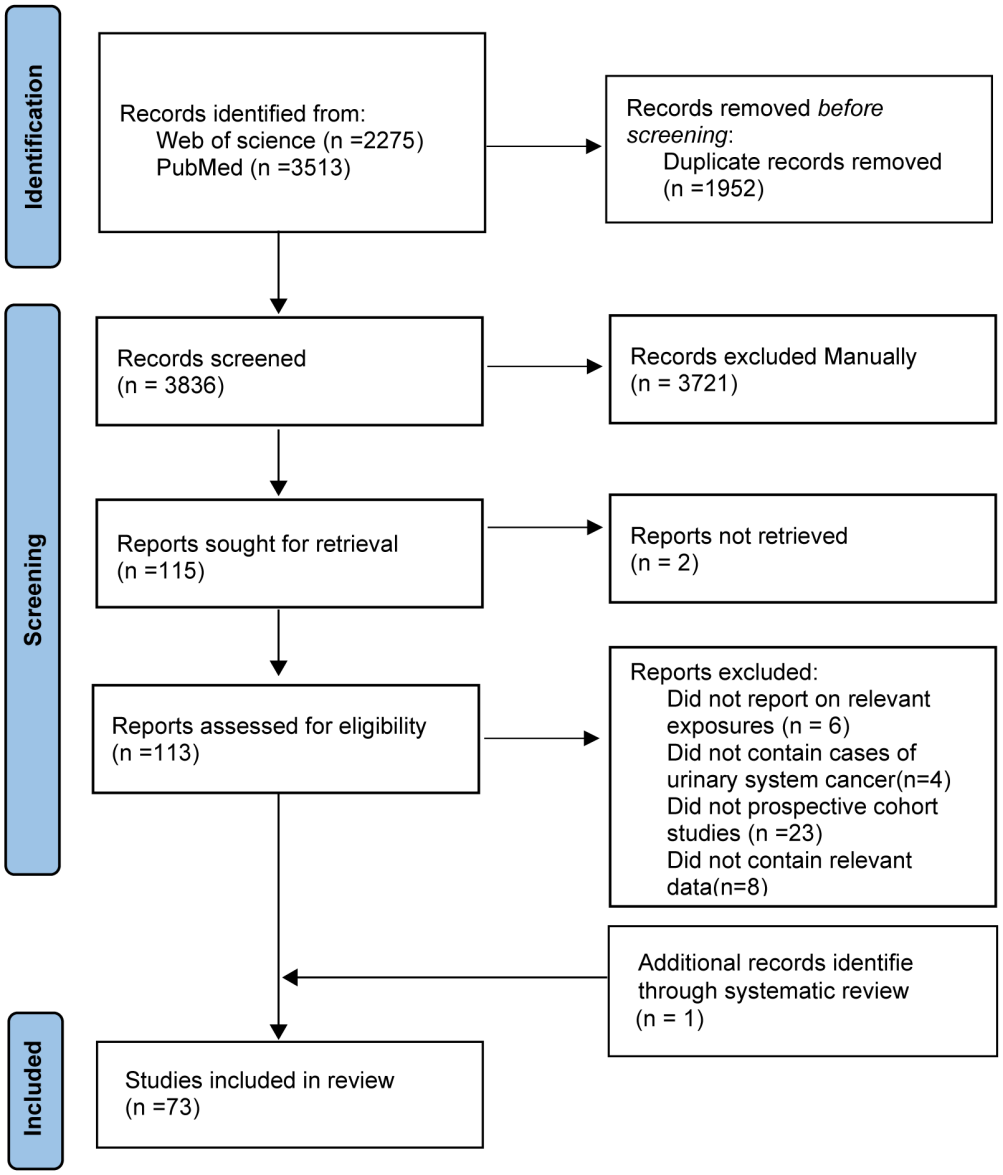
Flowchart of study selection.

The selection of the studies and the outcomes of the literature search were reported in Figure 1. In total, the original search turned up 5,788 articles. Duplicate papers and those that did not fit the criteria for inclusion have been removed. 113 full-text articles from potentially relevant studies were further evaluated. After a full-text review, additional 41 articles were excluded (Supplementary Appendix S2). And one additional record was added through systematic review. At last, 73 publications were included in the meta-analysis. During a mean of 13.6 years of follow-up, 4,903,674 participants were documented, of which 15,666 cases were ascertained (Supplementary Table S2). We evaluated the quality of the studies and yielded an average score of 8.38. Details of quality scores for all included studies are presented in Supplementary Table S3.

### 3.2. Foods associated with increased urologic cancers risk

#### 3.2.1. Red meat

**Figure 2:**
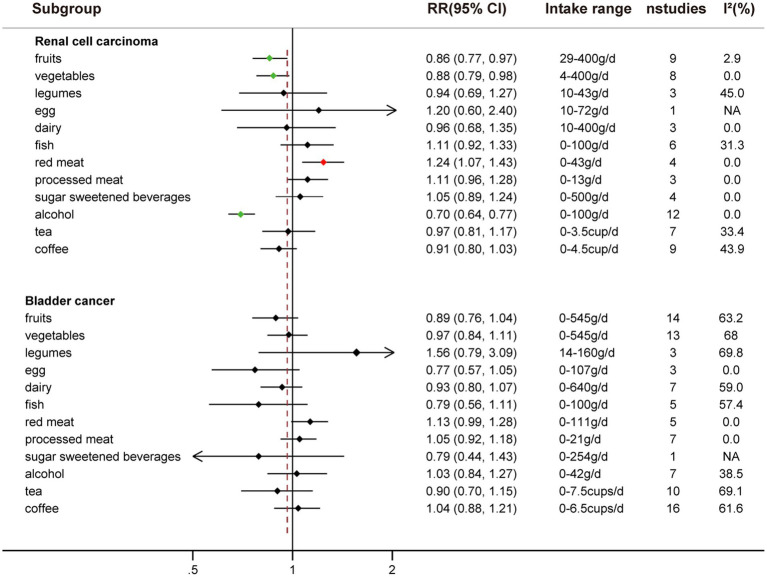
The highest versus lowest meta-analysis of food groups and the risk of RCC and BC.

**Figure 3:**
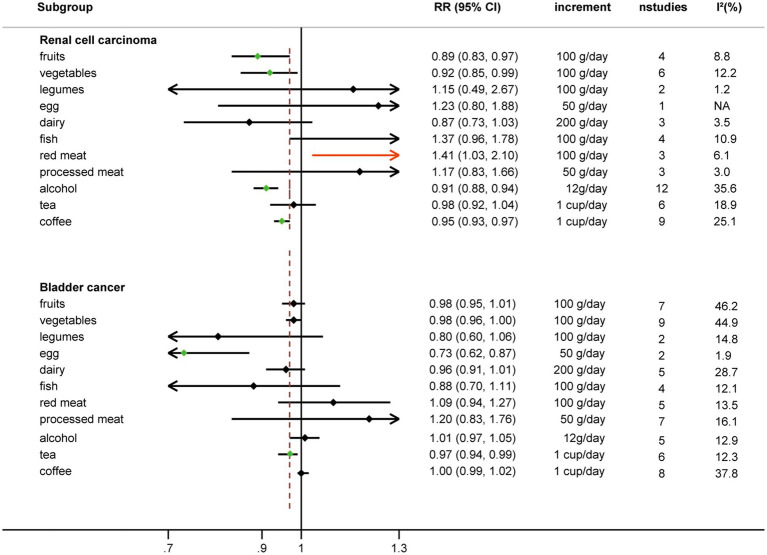
Linear dose–response of food groups and the risk of RCC and BC.

**Figure 4:**
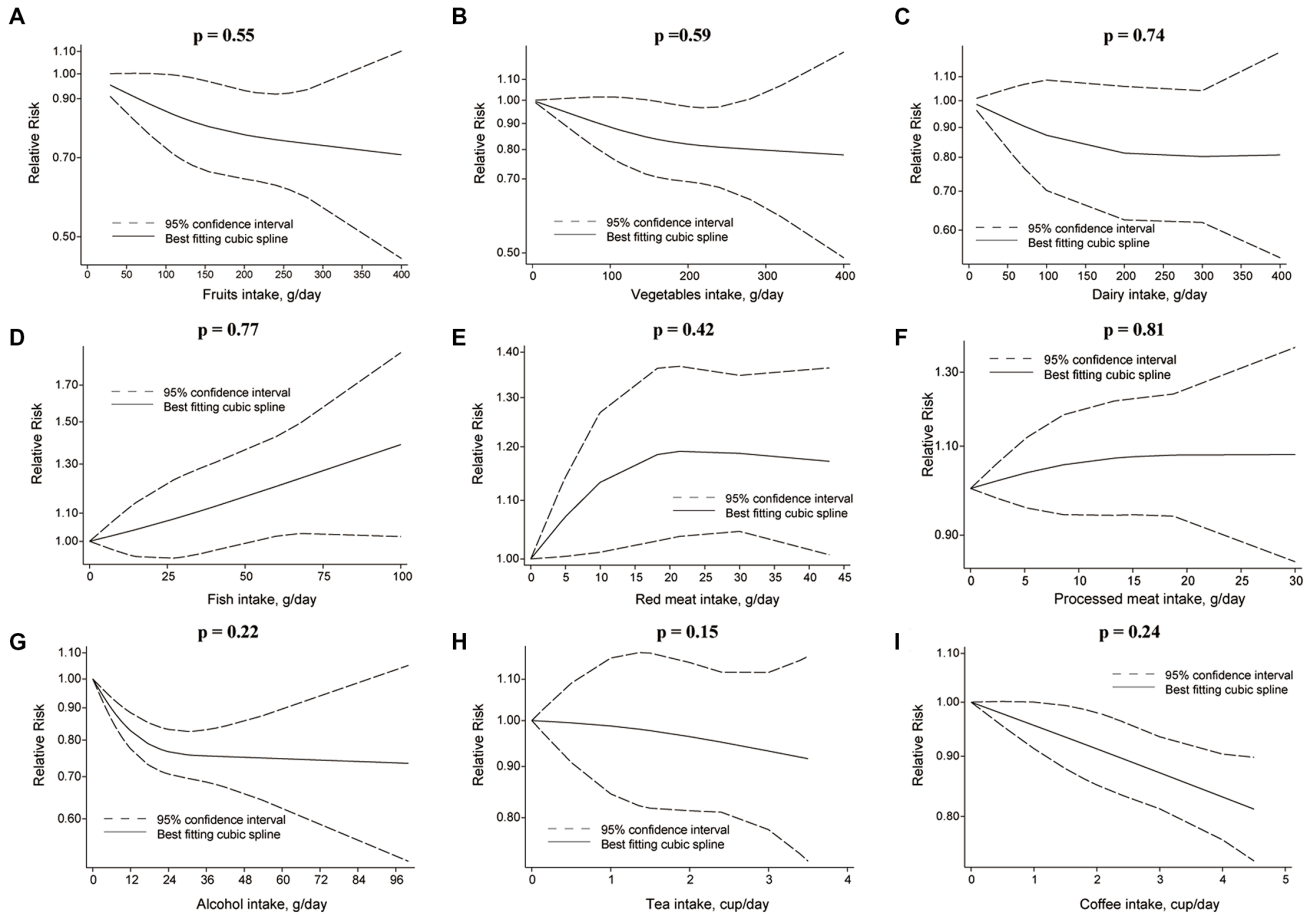
Non-linear dose–response relationship between food groups and risk of RCC. **(A)** Fruits, **(B)** vegetables, **(C)** dairy, **(D)** fish, **(E)** red meat, **(F)** processed meat, **(G)** alcohol, **(H)** tea, and **(I)** coffee.

**Figure 5:**
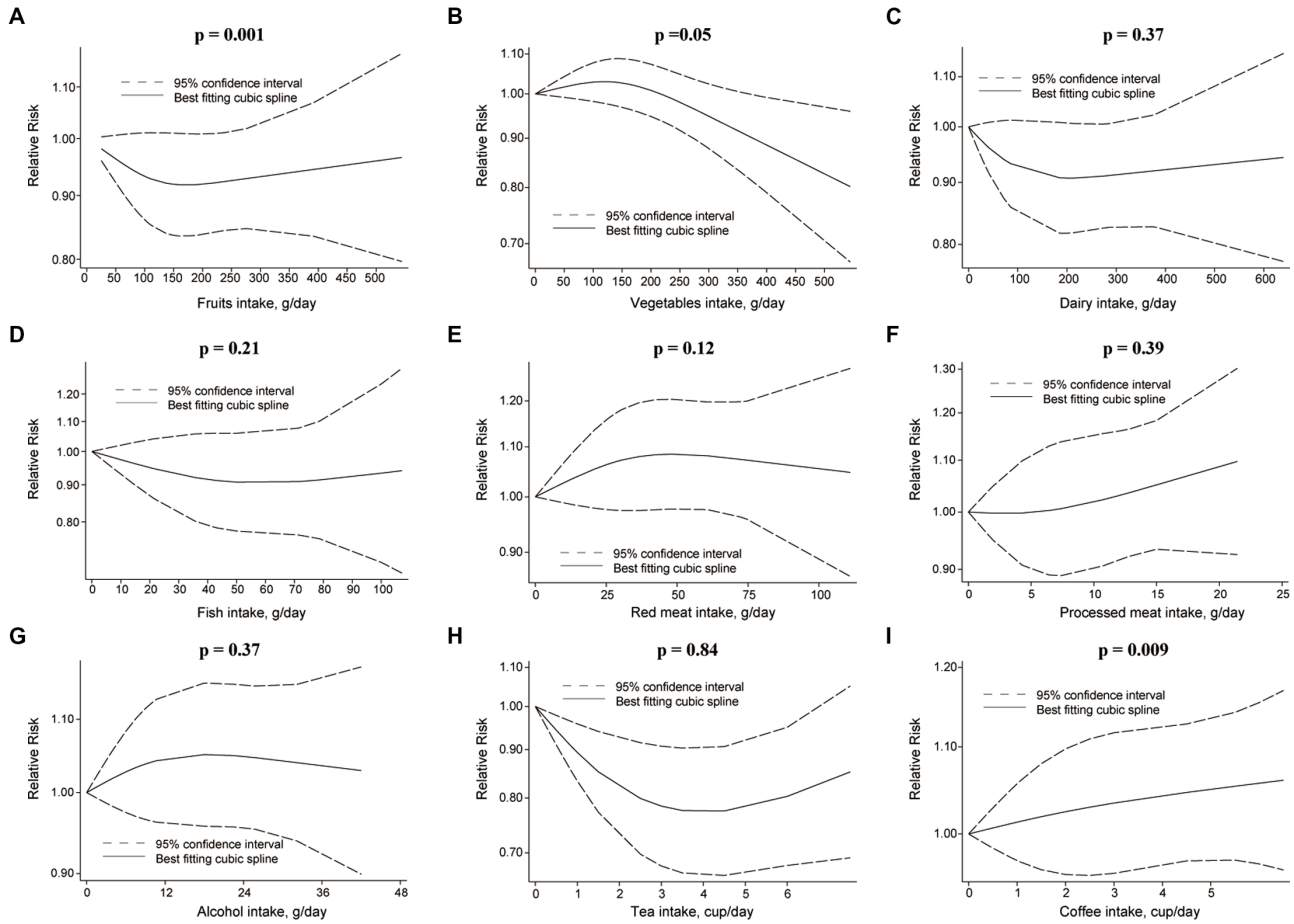
Non-linear dose–response relationship between food groups and risk of BC. **(A)** Fruits, **(B)** vegetables, **(C)** dairy, **(D)** fish, **(E)** red meat, **(F)** processed meat, **(G)** alcohol, **(H)** tea, and **(I)** coffee.

Comparing extreme intake categories (0 g/d vs. 43 g/d), a positive association was observed between red meat consumption and risk of RCC [RR: 1.24, 95%CI = (1.07, 1.43)] (Figure 2; Supplementary Figure S1). A positive association was found for each additional 100 g/d of red meat consumption and risk of RCC [RR: 1.41, 95%CI = (1.03, 2.10)], but not for BC [RR: 1.09, 95%CI = (0.94, 1.27)] (Figure 3). No nonlinear dose–response relationship was found between red meat consumption and RCC (*p* = 0.42) (Figure 4E) or BC (*p* = 0.12) (Figure 5E).

### 3.3. Foods associated with decreased urologic cancers risk

#### 3.3.1. Fruits

An inverse association was observed between fruits consumption and RCC risk when extreme intake categories were compared [RR: 0.86, 95%CI = (0.77, 0.97)] (Figure 2; Supplementary Figure S2). However, we found that fruit intake did not reduce the risk of BC when comparing the highest and lowest intake categories [RR: 0.89, 95%CI = (0.76, 1.04)] (Figure 2; Supplementary Figure S4).

Each additional 100 g/d of fruits was inversely associated with RCC risk [RR: 0.89, 95%CI = (0.83, 0.97)] (Figure 3), but not for BC risk [RR: 0.98, 95%CI = (0.95, 1.01)]. Nonetheless, fruits intake and RCC risk did not appear to have a nonlinear dose–response relationship (*p* = 0.55) (Figure 4A). Although fruit intake and risk of RCC did appear to be associated in a non-linear dose–response manner (*p* = 0.001) (Figure 5A), the CI overlaps with RR = 1, so it is not a statistically significant association.

In a stratified analysis of high-category versus low-category fruit intake and BC risk, there was no indication of heterogeneity between subgroups (I^2^ < 50%). These differences between the subgroups were not statistically significant (*p* > 0.05) (Supplementary Table S6).

#### 3.3.2. Vegetables

Comparing the highest to the lowest categories of vegetable intake, increased vegetable consumption was linked to a lower RCC risk [RR: 0.88, 95%CI = (0.79, 0.98)], but not for BC risk [RR: 0.97, 95%CI = (0.84, 1.11)] (Figure 2; Supplementary Figure S3). Additionally, vegetable intake was inversely correlated with the risk of RCC for each additional 100 g consumed daily [RR: 0.92, 95%CI = (0.85, 0.99)] (Figure 3). But each additional 100 g/d vegetable intake [RR: 0.98, 95%CI = (0.96, 1.00)] was not associated with the risk of BC (Figure 3). In stratified studies of vegetable intake and BC risk, there was no indication of heterogeneity between subgroups (Supplementary Table S7).

In the nonlinear dose–response meta-analysis, no association was observed between vegetable intake and RCC risk (*p* = 0.59) (Figure 4B) or BC risk (*p* = 0.05) (Figure 5B).

#### 3.3.3. Alcohol

An inverse association between alcohol consumption and risk of RCC was found when comparing the highest to lowest categories [RR: 0.70, 95%CI = (0.64, 0.77)] (Figure 2; Supplemental Figure S4). No correlation between alcohol consumption and the risk of BC was seen when the highest to lowest categories are compared [RR: 1.03, 95%CI = (0.84, 1.27)] (Figure 2; Supplementary Figure S4).

The risk of RCC was inversely correlated with the additional daily 12 g of alcohol intake [RR: 0.91, 95%CI = (0.88, 0.94)] (Figure 3). But no association was found between the additional daily 12 g of alcohol intake [RR: 1.01, 95%CI = (0.97, 1.05)] and the risk of BC (Figure 4). No non-linear dose–response association between alcohol consumption and RCC risk (*p* = 0.22) or BC risk was found (*p* = 0.37) (Figure 5).

#### 3.3.4. Tea

Comparing the highest to the lowest categories, no associations between tea intake and risk of RCC [RR: 0.97, 95%CI = (0.91, 1.17)] and BC [RR: 0.90, 95%CI = (0.70, 1.15)] were observed (Figure 2; Supplementary Figure S5).

An inverse association was observed for each additional daily 1 cup of tea and risk of BC [RR: 0.97, 95% CI = (0.94, 0.99)], but not for RCC [RR: 0.98, 95%CI = (0.92 to 1.04)] (Figure 3). There was no evidence of a non-linear dose–response association between tea and RCC risk (*p* = 0.15) (Figure 4H) or BC risk (*p* = 0.84) (Figure 5H).

No evidence of heterogeneity was detected between subgroups in stratified analyses on tea and bladder cancer (Supplementary Table S8).

#### 3.3.5. Coffee

While comparing the highest to lowest categories, there was no association between coffee consumption and RCC risk [RR: 0.91, 95%CI = (0.80, 1.03)] or BC risk [RR: 1.04, 95%CI = (0.88, 1.21)] (Figure 2; Supplementary Figure S6). The risk of RCC decreased with each additional daily cup of coffee [RR: 0.95, 95%CI = (0.93, 0.97)] (Figure 3). There was no correlation between the risk of BC and the additional daily cup of coffee [RR: 1.00, 95%CI = (0.99, 1.01)] (Figure 3). No non-linear dose–response association between coffee consumption and BC risk was found (*p* = 0.009) (Figure 5I). After stratification by Geographic location, heterogeneity was observed, demonstrating a positive association between coffee consumption and incidence of BC only in research conducted in Europe [RR: 1.19, 95%CI = (1.01, 1.39)] (Supplementary Table S5).

### 3.4. Foods not associated with urologic cancers risk

#### 3.4.1. Legumes

There was no association between legumes intake and risk of RCC [RR: 0.94, 95%CI = (0.69, 1.27)] or risk of BC [RR: 1.56, 95%CI = (0.79, 3.09)] when the highest and lowest categories of legumes intake were compared (Figure 2; Supplementary Figure S7). There was no correlation between each additional daily intake of 100 g of legumes and the risk of RCC [RR: 1.15, 95%CI = (0.49, 2.67)] or BC [RR: 0.80, 95%CI = (0.60, 1.06)] (Figure 3). Due to the limited availability of the data, non-linear dose–response meta-analysis was not applicable.

#### 3.4.2. Egg

No correlation between egg intake and risk of RCC [RR: 1.20, 95%CI = (0.60, 2.40)] or risk of BC [RR: 0.77, 95%CI = (0.57, 1.05)] was observed when comparing the highest to lowest categories of egg consumption (Figure 2; Supplementary Figure S8). An inverse association was found between each additional daily 50 g of egg consumption and the risk of BC [RR: 0.73, 95%CI = (0.62, 0.87)] (Figure 3). Due to the scarcity of data in prospective cohort studies, it was not possible to analyze the non-linear dose–response relationship between egg intake and urological cancers.

#### 3.4.3. Dairy

There was no association between dairy intake and the risk of RCC [RR: 0.96, 95%CI = (0.68, 1.35)], or BC [RR: 0.93, 95%CI = (0.80, 1.07)] when the highest and lowest categories of dairy intake were compared (Figure 2; Supplementary Figure S9). Each additional 200 g of dairy consumption daily did not affect the risk of RCC [RR: 0.87, 95%CI = (0.73, 1.03)] or BC [RR: 0.96, 95%CI = (0.91, 1.01)] (Figure 3). There was also no evidence of a non-linear dose–response relationship between dairy consumption and RCC risk (*p* = 0.74) (Figure 4C) or BC risk (*p* = 0.37) (Figure 5C). In stratified analyses of dairy consumption and BC risk, no significant evidence of heterogeneity was found between subgroups (Supplementary Table S9).

#### 3.4.4. Fish

Comparing the highest to the lowest categories, no association between fish intake and RCC risk [RR: 1.11, 95%CI = (0.92, 1.33)] or BC risk [RR: 0.79, 95% CI = (0.56, 1.11)] was observed (Figure 2; Supplementary Figure S10). Each additional daily 100 g of fish intake was not associated with the risk of RCC [RR: 1.37, 95% CI = (0.96, 1.78)] or BC [RR: 0.88, 95% CI = (0.70, 1.11)] (Figure 3).

There was no evidence of a non-linear dose–response association between fish intake and RCC risk (*p* = 0.77) (Figure 4D), or BC risk (*p* = 0.21) (Figure 5D). Subgroup analyses showed evidence of heterogeneity, for the prospective cohort studies in America and studies with ≥100,000 participants, a high intake of fish has been linked to a significantly lower risk of BC (Supplementary Table S4).

#### 3.4.5. Processed meat

There was no significant association between processed meat consumption and the risk of RCC [RR: 1.11, 95%CI = (0.96, 1.28)], or BC [RR: 1.05, 95%CI = (0.92, 1.18)] (Figure 2; Supplementary Figure S11). Each additional 50 g of processed meat consumed daily was not associated with a higher risk of RCC [RR: 1.17, 95%CI = (0.83, 1.66)], or BC [RR: 1.20, 95%CI = (0.83, 1.76)] (Figure 3). There was no nonlinear dose–response relationship between processed meat intake and RCC risk (*p* = 0.81) (Figure 4F), or BC risk (*p* = 0.39) (Figure 5F).

#### 3.4.6. Sugar-sweetened beverages

There was no association between SSB intake and the risk of RCC [RR: 1.05, 95%CI = (0.89, 1.24)] or BC [RR: 0.79, 95%CI = (0.44, 1.43)] when the highest and lowest categories were compared (Figure 2; Supplementary Figure S12). In addition, dose–response meta-analysis was not possible due to a lack of data availability.

### 3.5. Publication bias and sensitivity analysis

Based on the funnel plot (Supplementary Figures S13–S17) and Egger’s test, there was no publication bias for alcohol intake and risk of RCC (*p* = 0.848, *n* = 12 studies), fruit (*p* = 0.402, *n* = 14 studies), vegetables (*p* = 0.469, *n* = 13 studies), tea (*p* = 0.186, *n* = 10 studies), coffee (*p* = 0.748, *n* = 16 studies) intake for BC. In the influence analysis in which we excluded one study from high versus low meta-analysis with high heterogeneity (I^2^ ≥ 50%), in turn, the summary estimates were not substantially altered for all of the exposures (fruits, vegetables, legumes, dairy, fish, tea, and coffee) (Supplementary Figures S18–S24).

### 3.6. Quality of evidence

We graded and assessed the quality of meta-evidence regarding the association between food groups and the risk of RCC and BC. In our results, the classification of RCC’s NutriGrade meta-evidence was given as follows: “high” for alcohol, “moderate” for fruits, vegetables, red meat, and coffee, and “low” for the other seven food groups (Table 1). The NutriGrade grading on BC was rated “moderate” for fruits, vegetables, tea, and coffee, and “low” for the 8 other food groups (Table 2).

**Table 1:** NutriGrade assessment of confidence in estimate effect of studies evaluated the association between various food groups and risk of RCC.

Food groups	Risk of bias^1^	Precision^2^	Indirectness	Heterogeneity^3^	Publication bias	Effect size	Dose response	Funding bias	Total score	Confidence evidence^4^
Fruits	2	1	1	0.5	0.5	0	1	1	7	Moderate
Vegetables	2	1	1	0.5	0.5	0	1	1	7	Moderate
Legumes	2	0	1	0	0	0	0	1	4	Low
Egg	2	0	1	0	0	0	0	1	4	Low
Dairy	2	0	1	0	0	0	0	1	4	Low
Fish	2	0	1	0.5	0.5	0	0	1	5	Low
Red meat	2	1	1	1	0	0	1	1	7	Moderate
Processed meat	2	0	1	0	0	0	0	1	4	Low
Sugar-sweetened drinks	2	0	1	0	0	0	0	1	4	Low
Alcohol	2	1	1	1	1	1	1	1	9	High
Tea	2	0	1	0.5	0.5	0	0	1	5	Low
Coffee	2	0	1	0.5	0.5	0	1	1	6	Moderate

NutriGrade, Nutrition Grading of Recommendations Assessment, Development and Evaluation; RR, risk ratio.

1 Risk of bias was based on the Newcastle-Ottawa Scale, where ≥ 7 = 2 points; 4–6.9 = 1 point; and 0–3.9 = 0 points.

2 Precision is 1 point if the number of events ≥500 and the 95% CI excludes the null value; precision is 0 points if the number of events <500 or number of events ≥500, but 95% CI includes the null value and 95% CI fails to exclude an important benefit (RR of 0.8) or harm (RR of 1.2).

3 Based on the funnel plots, Egger or Begg’s test. For the outcomes with small number of studies (*n* < 10), the risk of publication bias was not formally assessed.

4 High quality indicates that there is high confidence in the effect estimate, and further research probably will not change the confidence in the effect estimate. Moderate quality indicates that we are moderately confident in the effect estimate: the true effect is likely to be close to the estimate of the effect, but there is a possibility that it is substantially different. Low quality indicates that our confidence in the effect estimate is limited: the true effect may be substantially different from the estimate of the effect.

**Table 2:** NutriGrade assessment of confidence in estimate effect of studies evaluated the association between various food groups and risk of BC.

Food groups	Risk of bias^1^	Precision^2^	Indirectness	Heterogeneity	Publication bias^3^	Effect size	Dose response	Funding bias	Total score	Confidence evidence^4^
Fruits	2	0	1	1	1	0	0	1	6	Moderate
Vegetables	2	0	1	1	1	0	0	1	6	Moderate
Legumes	2	0	1	0	0	0	0	1	4	Low
Egg	2	0	1	0	0	0	1	1	5	Low
Dairy	2	0	1	0	0	0	0	1	4	Low
Fish	2	0	1	0	0	0	0	1	4	Low
Red meat	2	0	1	0	0	0	0	1	4	Low
Processed meat	2	0	1	0.5	0.5	0	0	1	5	Low
Sugar-sweetened drinks	2	0	1	0	0	0	0	1	4	Low
Alcohol	2	0	1	0	0	0	0	1	4	Low
Tea	2	0	1	1	1	0	1	1	7	Moderate
Coffee	2	0	1	1	1	0	0	1	6	Moderate

NutriGrade, Nutrition Grading of Recommendations Assessment, Development and Evaluation; RR, risk ratio.

1 Risk of bias was based on the Newcastle-Ottawa Scale, where ≥7 = 2 points; 4–6.9 = 1 point; and 0–3.9 = 0 points.

2 Precision is 1 point if the number of events ≥500 and the 95% CI excludes the null value; precision is 0 points if the number of events <500 or number of events ≥500, but 95% CI includes the null value and 95% CI fails to exclude an important benefit (RR of 0.8) or harm (RR of 1.2).

3 Based on the funnel plots, Egger or Begg’s test. For the outcomes with small number of studies (*n* < 10), the risk of publication bias was not formally assessed.

4 High quality indicates that there is high confidence in the effect estimate, and further research probably will not change the confidence in the effect estimate. Moderate quality indicates that we are moderately confident in the effect estimate: the true effect is likely to be close to the estimate of the effect, but there is a possibility that it is substantially different. Low quality indicates that our confidence in the effect estimate is limited: the true effect may be substantially different from the estimate of the effect.

## 4. Discussion

### 4.1. Principal findings

In this study, we evaluated the associations of targeted food groups—fruits, vegetables, legumes, egg, dairy, fish, red meat, processed meat, SSB, alcohol, tea, and coffee—and the risk of RCC and BC. First, we found that fruits, vegetables, and alcohol were associated with a decreased risk of RCC in the high versus low meta-analysis, while red meat was associated with an increased risk of RCC. Second, in the linear dose–response meta-analysis, an inverse association was found between fruits, vegetables, alcohol, coffee intake and risk of RCC, and a positive association was found between red meat intake and RCC. Differently, tea consumption was negatively associated with the risk of BC. At last, there were no indications for nonlinear dose–response relationships between preselected food groups intake and risk of RCC and BC.

The NutriGrade tool suggested a high confidence in the estimate of the alcohol intake and risk of RCC, and moderate confidence in the estimate of the effect of fruits, vegetables, tea, and coffee intake for the risk of RCC. The NutriGrade tool for BC was classified as “moderate” for fruits, vegetables, tea, and coffee, and the confidence for other food groups was lower.

### 4.2. Strengths of the study

The study has some advantages. First, the article is the first meta-analysis that includes all the available prospective cohort studies to estimate the connection between food groups and the risk of urologic cancers.

Of note, we only included urologic cancers that occur in both men and women, which minimizes gender differences. Furthermore, we performed various types of analyses that enable us to thoroughly identify the associations between food groups and urological cancers and found an ideal intake with the lowest risk. This meta-analysis included prospective studies only, the recall bias was successfully avoided, and the likelihood of selection bias was decreased (27). Additionally, the overall quality of evidence is further ensured by the Newcastle-Ottawa scale assessment (8.38 on average).

### 4.3. Comparison with other studies

Our findings are consistent with earlier meta-analyses that showed an inverse association between fruits and vegetable intake and RCC risk (28–32). While other research indicates that the incidence of urologic cancers is not associated with overall fruit and vegetable consumption (11–13, 33–37). The base of the dietary pyramid, fruits and vegetables, contain many compounds that may prevent cancers, yet it is challenging to determine the proportional value of each component. Any preventative impact could most likely be attributed to a confluence of actions on many carcinogenesis-related pathways. Numerous antioxidant elements (including carotenoids and vitamin C), minerals, dietary fiber, phenols, flavonoids, and phytochemicals are present in many fruits and vegetables, which may influence the processes governing cell proliferation and death (38). These processes are assumed to be primarily responsible for the association between consuming fruits and vegetables and a decreased risk of urological cancers. For example, cruciferous vegetables have high levels of glucosinolates, which are converted into isothiocyanates by the enzyme myrosinase during food preparation and change the way carcinogens are metabolized, which could decrease the risk of cancers (39).

No association was found in our meta-analysis between dairy consumption and RCC and BC risk. Similarly, previous studies found no association between BC risk and milk consumption (40, 41), whereas some previous meta-analyses suggested dairy (such as milk) consumption is positively associated with the risk of BC (42–44). Calcium, vitamins, and protein are the elements found in dairy, which may benefit human health (45). Suggestions on dairy consumption to urologic cancers cannot yet be made due to conflicting results.

In this meta-analysis, we found a statistically significant positive association between red meat intake and RCC. A positive association has previously been reported between red meat consumption and the risk of RCC (46, 47), while some meta-analyses found no associations between red meat intake and the risk of RCC (48). The difference could be explained by the inclusion of prospective studies only. However, we observed no associations between red meat and processed meat intake and the risk of BC. Meat plays a significant role in human nutrition because it offers high-quality protein as well many vital minerals, including iron, zinc, and vitamin B12 (49). Nevertheless, a high intake of red and processed meat is associated with an increased risk for diseases. A recent study has found the consumption of Neu5Gc (N-Glycolylneuraminic acid) from red meat increases the risk of cancers (50). On the cell surface of the majority of mammals, Neu5Gc exists naturally. Due to the inactivation of the gene encoding CMP-N-acetylneuraminic acid hydroxylase, it is not present in human tissues. Whenever people eat too much red meat, Neu5Gc enters cells, where the immune system recognizes it as a foreign threat and produces antibodies to destroy it. Repeated consumption of these meats will trigger this immune response, leading to long-term chronic inflammation and an increased risk of tumor formation (51, 52).

The association between alcohol intake and the risk of BC is not consistent. A meta-analysis reported that in males, alcohol may increase the risk of BC in a dose-independent manner (53), whereas another study reported that there is no material relationship between high levels of alcohol consumption and BC risk (17). No significant association was found between alcohol consumption and BC risk in our results, which is consistent with previous meta-analyses (54). However, there was a statistically significant and persuasive relationship between alcohol and RCC. The findings from our meta-analysis support the previous hypothesis that alcoholic beverage intake is inversely associated with risk of RCC (17, 55, 56).

There are various theories as to how alcohol may lower the risk of kidney cancer, but the exact processes remain elusive: (1) Moderate alcohol consumption is linked to lower rates of type 2 diabetes and hyperinsulinemia, which may be risk factors for kidney cancer; (2) Antioxidant phenolic substances that can prevent cell cycle progression and reduce oxidative stress may be present in alcoholic beverages (57, 58); (3) The diuretic impact of alcohol, which increases urine volume and shortens exposure times, is another theory that might apply (59). (4) It is worth noting that the relationship between alcohol intake and RCC risk would seem to be influenced by inter-individual germline variation in alcohol-metabolizing genes (60). Taken together, to corroborate our findings, further investigate other particular demographic groups, and identify possible regulator genes or biomarkers based on molecular epidemiology, additional high-quality studies should be carried out.

Our findings did not support the conclusion that tea consumption is related to a decreased risk of RCC, which is consistent with previous studies (61, 62). Our findings of an inverse correlation between tea consumption and BC were indeed consistent with the findings of several previous studies (63, 64). Studies in animals have demonstrated that some tea constituents may have a restraining effect on BC development (65). This inhibitory activity is believed to be primarily due to the antioxidative and possibly antiproliferative effects of polyphenol compounds (such as epigallocatechin gallate), through inhibition of metabolic or signal-transduction pathways (66, 67).

In our meta-analysis, there was no significant statistical association between coffee drinking and BC risk, and no correlation was detected in earlier cohort studies (68–70). Our results support that the consumption of coffee is associated with a reduced risk of RCC. Results from previous analyses provide evidence of the benefit of caffeinated coffee (71), while other studies demonstrate no significant association between coffee consumption and RCC (72). According to epidemiological research, coffee consumption was inversely associated with the risk of several cancers (73). Many studies have suggested mechanisms by which coffee intake reduces RCC risk. For example, the presence of phytochemicals, such as caffeine, chlorogenic acid, and caffeic acid, may be responsible for enhanced insulin sensitivity (74). Furthermore, convincing evidence showed that being overweight may increase the risk of developing RCC (75). However, caffeine may enhance energy balance by suppressing appetites, raising basal metabolic rate, and stimulating food-induced thermogenesis to regulate weight (76). The exact mechanism by which obesity raises the risk of RCC is uncertain, but the accumulation of body fat directly affects insulin levels in the body, thereby elevating the probability of hypertension, both of which are strongly associated with the development of RCC (77, 78).

The high versus low meta-analysis and dose–response analysis revealed no association between legumes and the risk of RCC or BC. To support our findings, additional well-designed prospective studies will be required, taking into account the limitations of the included research (69). In addition to being an excellent source of fiber, legumes also contain certain bioactive substances, which are peptides formed from proteins that have been shown in *in vitro* experiments to have antioxidant effects (79). We observed an inverse association between egg consumption and BC risk, which is different from previous meta-analyses (80, 81). This inconsistency could be explained by the inclusion of new studies. Egg yolks contain accessible xanthophyll carotenoids, which have anti-inflammatory and antioxidant properties and may be able to prevent cancers (82). Taking into account the limitations of the included studies and the low credibility of meta-evidence confidence, this result should be considered with caution. In our meta-analysis, no association was found between fish consumption and RCC risk or BC risk. Similarly, a previous cohort study has found no association between BC risk and fish consumption (83). On the contrary, an investigation reported a beneficial effect (84). Multiple studies have suggested that ω-3 fatty acids which is abundant in fatty fish may have a reducing influence on the chance of developing cancer (84–86). Further well-designed prospective studies are required to further investigate the impact of fish on RCC and BC due to the dearth of studies in this area.

### 4.4. Limitations of the study

Unfortunately, this study has some technical and biological limitations. First, substantial heterogeneity exists in some analyses, which could not be further explored due to the limited number of studies. The heterogeneity between studies was not completely omitted after subgroup analysis, and the interpretation of the findings should be done with caution. Second, food frequency questionnaires, which rely on recall are a common source for estimates of food category intake. Therefore, measurement errors seemed inevitable and can lead to the misclassification of exposures. In addition, since the food categories in each food questionnaire were not fully standardized, the food items in our meta-analysis were only counted according to broad categories, and the completeness and credibility of this analysis would be higher if more detailed and standardized food categories were available in the future. Of note, sex is a significant factor in epidemiological studies, but the data were not sufficient to support analyses by sex of the meta-analysis. As a result, some of these results need to be evaluated carefully. Therefore, it is meaningful to design more effective and comprehensive prospective cohort studies to investigate associations between food groups and urological cancers risk.

## 5. Conclusion

Overall, our meta-analysis collectively tends to show some correlation between food group intake and urological cancers. We found that a high intake of red meat increases the risk of RCC. High fruit, vegetable, alcohol, and coffee intake may play a protective role against RCC. A high intake of tea may decrease the risk of BC. For urethral cancer and renal pelvis carcinoma, the number of related studies is too small to support meta-analysis. In summary, these findings may contribute to developing food-based dietary recommendations for preventing urologic cancers.

## Author contributions

JQ: conceptualization, investigation, data curation, software, and writing–original draft. PA: conceptualization and methodology. DJ: data curation and writing–original draft preparation. YJ: visualization and investigation. SW and XZ: software and validation. YL: writing-reviewing and funding acquisition. JL: writing–reviewing and editing. CZ: supervision and project administration. All authors contributed to the article and approved the submitted version.

## Funding

This work was supported by the Beijing Advanced Innovation Center for Food Nutrition and Human Health, the National Natural Science Foundation of China (31970717 and 82170429), the Chinese Universities Scientific Fund (2020TC015), and the Beijing Municipal Natural Science Foundation (7222111).

## Conflict of interest

The authors declare that the research was conducted in the absence of any commercial or financial relationships that could be construed as a potential conflict of interest.

## Publisher’s note

All claims expressed in this article are solely those of the authors and do not necessarily represent those of their affiliated organizations, or those of the publisher, the editors and the reviewers. Any product that may be evaluated in this article, or claim that may be made by its manufacturer, is not guaranteed or endorsed by the publisher.
