# Supplementary material for: Food groups and urologic cancers risk: a systematic review and meta-analysis of prospective studies
Source: Front Nutr. 2023 May 17;10:1154996. doi: 10.3389/fnut.2023.1154996 (PMC10231388; doi:10.3389/fnut.2023.1154996)
Supplement: Supplementary file 1 [file Data_Sheet_1.docx]

**Supplementary materials**

**Supplemental Appendix 1:**

Search strategy

#1 (food) OR (vegetable)) OR (potato) OR (fruit) OR (legume) OR (bean) OR (soy) OR (egg) OR (dairy) OR (dairies) OR (milk) OR (yogurt) OR (cheese) OR (fish)OR (seafood) OR (meat) OR (processed meat) OR (sugar sweetened beverage) OR（soft drink）OR (coffee) OR (tea) OR (alcohol)

#2 (prospective study) OR (follow up) OR (cohort study) OR (longitudinal study) OR (cohort) OR (prospective) OR (longitudinal) OR(nested case-control)

#3 (renal cell carcinoma) OR (nephroid carcinoma) OR (adenocarcinoma of kidney) OR (renal cell cancer) OR(kidney cancer)OR(renal cancer)OR(kidney Neoplasm)OR(Renal Neoplasm) OR (renal adenocarcinoma) OR (renal carcinoma) OR (renal cell adenocarcinoma) OR (papillary renal cell carcinoma) OR (Chromophil Renal Cell Carcinoma) OR (Clear Cell Renal Cell Carcinoma) OR (Hypernephroid Carcinoma) OR (Hypernephroma) OR (Collecting Duct Carcinoma of the Kidney) OR (Collecting Duct Carcinoma) OR (Collecting Duct Carcinomas) OR (Ureteral Neoplasm) OR (Ureter Neoplasm) OR (Neoplasm, Ureteral) OR (Cancer of Ureter) OR (Ureter Cancer) OR (Ureteral Cancer) OR (Urinary Bladder Neoplasms) OR (Urinary Bladder Neoplasm) OR (Bladder Tumor) OR (Bladder Neoplasm) OR (Urinary Bladder Cancer) OR (Bladder Cancer) OR (Cancer of Bladder) OR (urothelial carcinoma of the renal pelvis) OR (urothelial carcinoma of the kidney pelvis)

#4（#1AND#2AND#3）

**Supplemental Appendix 2：Full-text articles excluded**

| Reference | Reason for exclusion |
| --- | --- |
| (Adami et al., 1992; Allen et al., 2013; An & Arellano, 2021; Dianatinasab et al., 2022; Jochems et al., 2018; Zhou et al., 2014) | Not report on relevant exposure |
| (Åsli et al., 2018; Park et al., 2017; Townsend et al., 2013; Wu et al., 2022) | Not contain cases of urinary system cancer |
| (Abid et al., 2014; Abufaraj et al., 2019; Acham et al., 2020; Alicandro et al., 2017; Allam, 2005; Allen et al., 2009; Buykx et al., 2016; Fraser, 1999; Hirayama, 1986; Hsu et al., 2007; Jochems et al., 2020; Lee et al., 2008, 2009; Lee, Hunter, Spiegelman, Adami, Albanes, et al., 2007; Lee, Hunter, Spiegelman, Adami, Bernstein, et al., 2007; Washio et al., 2014; Weng et al., 2016; Wolk et al., 1996; J. Wu et al., 2020; S. Wu et al., 2019; Xu et al., 2015; E. Y. W. Yu, Dai, et al., 2020; E. Y. W. Yu, Wesselius, et al., 2020; E. Y.-W. Yu et al., 2021) | Not prospective cohort studies |
| (Abbaoui et al., 2017; Büchner et al., 2011; Donat et al., 2003; Jochems et al., 2018b; Nomura et al., 1986; Tang et al., 2010; Jacobsen et al., 1986; Weikert et al., 2006; ) | Not contain relevant data |

**References**

Abid, Z., Cross, A. J., & Sinha, R. (2014). Meat, dairy, and cancer. *The American Journal of Clinical Nutrition*, *100 Suppl 1*, 386S-93S.

Abufaraj, M., Tabung, F. K., Shariat, S. F., Moschini, M., Devore, E., Papantoniou, K., Yang, L., Strohmaier, S., Rohrer, F., Markt, S. C., Zhang, X., Giovannucci, E., & Schernhammer, E. (2019). Association between Inflammatory Potential of Diet and Bladder Cancer Risk: Results of 3 United States Prospective Cohort Studies. *The Journal of Urology*, *202*(3), 484–489.

Acham, M., Wesselius, A., van Osch, F. H. M., Yu, E. Y.-W., van den Brandt, P. A., White, E., Adami, H.-O., Weiderpass, E., Brinkman, M., Giles, G. G., Milne, R. L., & Zeegers, M. P. (2020). Intake of milk and other dairy products and the risk of bladder cancer: A pooled analysis of 13 cohort studies. *European Journal of Clinical Nutrition*, *74*(1), 28–35.

Adami, H. O., McLaughlin, J. K., Hsing, A. W., Wolk, A., Ekbom, A., Holmberg, L., & Persson, I. (1992). Alcoholism and cancer risk: A population-based cohort study. *Cancer Causes & Control: CCC*, *3*(5), 419–425.

Alicandro, G., Tavani, A., & La Vecchia, C. (2017). Coffee and cancer risk: A summary overview. *European Journal of Cancer Prevention: The Official Journal of the European Cancer Prevention Organisation (ECP)*, *26*(5), 424–432.

Allam, M. F. (2005). Fluid intake and urinary bladder cancer. *European Journal of Cancer Prevention: The Official Journal of the European Cancer Prevention Organisation (ECP)*, *14*(1), 77–78.

Allen, N. E., Appleby, P. N., Key, T. J., Bueno-de-Mesquita, H. B., Ros, M. M., Kiemeney, L. A. L. M., Tjønneland, A., Roswall, N., Overvad, K., Weikert, S., Boeing, H., Chang-Claude, J., Teucher, B., Panico, S., Sacerdote, C., Tumino, R., Palli, D., Sieri, S., Peeters, P., … Riboli, E. (2013). Macronutrient intake and risk of urothelial cell carcinoma in the European prospective investigation into cancer and nutrition. *International Journal of Cancer*, *132*(3), 635–644.

Allen, N. E., Roddam, A. W., Sieri, S., Boeing, H., Jakobsen, M. U., Overvad, K., Tjønneland, A., Halkjaer, J., Vineis, P., Contiero, P., Palli, D., Tumino, R., Mattiello, A., Kaaks, R., Rohrmann, S., Trichopoulou, A., Zilis, D., Koumantaki, Y., Peeters, P. H., … Riboli, E. (2009). A prospective analysis of the association between macronutrient intake and renal cell carcinoma in the European Prospective Investigation into Cancer and Nutrition. *International Journal of Cancer*, *125*(4), 982–987.

An, T. J., & Arellano, R. S. (2021). Comparison of Safety and Efficacy of Percutaneous Microwave Ablation of Central Versus Peripheral Renal Cell Carcinoma. *Cardiovascular and Interventional Radiology*, *44*(2), 281–288.

Buykx, P., Li, J., Gavens, L., Hooper, L., Lovatt, M., Gomes de Matos, E., Meier, P., & Holmes, J. (2016). Public awareness of the link between alcohol and cancer in England in 2015: A population-based survey. *BMC Public Health*, *16*(1), 1194.

Dianatinasab, M., Wesselius, A., Salehi-Abargouei, A., Yu, E. Y. W., Fararouei, M., Brinkman, M., van den Brandt, P., White, E., Weiderpass, E., Le Calvez-Kelm, F., Gunter, M. J., Huybrechts, I., & Zeegers, M. P. (2022). Dietary fats and their sources in association with the risk of bladder cancer: A pooled analysis of 11 prospective cohort studies. *International Journal of Cancer*, *151*(1), 44–55.

Fraser, G. E. (1999). Associations between diet and cancer, ischemic heart disease, and all-cause mortality in non-Hispanic white California Seventh-day Adventists. *The American Journal of Clinical Nutrition*, *70*(3 Suppl), 532S-538S.

Hirayama, T. (1986). [A large scale cohort study of dietary habits and cancer mortality]. *Gan No Rinsho. Japan Journal of Cancer Clinics*, *32*(6), 610–622.

Hsu, C. C., Chow, W.-H., Boffetta, P., Moore, L., Zaridze, D., Moukeria, A., Janout, V., Kollarova, H., Bencko, V., Navratilova, M., Szeszenia-Dabrowska, N., Mates, D., & Brennan, P. (2007). Dietary risk factors for kidney cancer in Eastern and Central Europe. *American Journal of Epidemiology*, *166*(1), 62–70.

Jochems, S. H. J., Reulen, R. C., van Osch, F. H. M., Witlox, W. J. A., Goossens, M. E., Brinkman, M., Giles, G. G., Milne, R. L., van den Brandt, P. A., White, E., Weiderpass, E., Huybrechts, I., Hémon, B., Agudo, A., Bueno-de-Mesquita, B., Cheng, K. K., van Schooten, F. J., Bryan, R. T., Wesselius, A., & Zeegers, M. P. (2020). Fruit consumption and the risk of bladder cancer: A pooled analysis by the Bladder Cancer Epidemiology and Nutritional Determinants Study. *International Journal of Cancer*, *147*(8), 2091–2100.

Jochems, S. H. J., van Osch, F. H. M., Reulen, R. C., van Hensbergen, M., Nekeman, D., Pirrie, S. J., Wesselius, A., van Schooten, F. J., James, N. D., Wallace, D. M. A., Bryan, R. T., Cheng, K. K., & Zeegers, M. P. (2018). Total Fluid Intake and the Risk of Recurrence in Patients With Non-Muscle Invasive Bladder Cancer: A Prospective Cohort Study. *Bladder Cancer (Amsterdam, Netherlands)*, *4*(3), 303–310.

Lee, J. E., Hunter, D. J., Spiegelman, D., Adami, H.-O., Albanes, D., Bernstein, L., van den Brandt, P. A., Buring, J. E., Cho, E., Folsom, A. R., Freudenheim, J. L., Giovannucci, E., Graham, S., Horn-Ross, P. L., Leitzmann, M. F., McCullough, M. L., Miller, A. B., Parker, A. S., Rodriguez, C., … Smith-Warner, S. A. (2007). Alcohol intake and renal cell cancer in a pooled analysis of 12 prospective studies. *Journal of the National Cancer Institute*, *99*(10), 801–810.

Lee, J. E., Hunter, D. J., Spiegelman, D., Adami, H.-O., Bernstein, L., van den Brandt, P. A., Buring, J. E., Cho, E., English, D., Folsom, A. R., Freudenheim, J. L., Gile, G. G., Giovannucci, E., Horn-Ross, P. L., Leitzmann, M., Marshall, J. R., Männistö, S., McCullough, M. L., Miller, A. B., … Smith-Warner, S. A. (2007). Intakes of coffee, tea, milk, soda and juice and renal cell cancer in a pooled analysis of 13 prospective studies. *International Journal of Cancer*, *121*(10), 2246–2253.

Lee, J. E., Männistö, S., Spiegelman, D., Hunter, D. J., Bernstein, L., van den Brandt, P. A., Buring, J. E., Cho, E., English, D. R., Flood, A., Freudenheim, J. L., Giles, G. G., Giovannucci, E., Håkansson, N., Horn-Ross, P. L., Jacobs, E. J., Leitzmann, M. F., Marshall, J. R., McCullough, M. L., … Smith-Warner, S. A. (2009). Intakes of fruit, vegetables, and carotenoids and renal cell cancer risk: A pooled analysis of 13 prospective studies. *Cancer Epidemiology, Biomarkers & Prevention: A Publication of the American Association for Cancer Research, Cosponsored by the American Society of Preventive Oncology*, *18*(6), 1730–1739.

Lee, J. E., Spiegelman, D., Hunter, D. J., Albanes, D., Bernstein, L., van den Brandt, P. A., Buring, J. E., Cho, E., English, D. R., Freudenheim, J. L., Giles, G. G., Graham, S., Horn-Ross, P. L., Håkansson, N., Leitzmann, M. F., Männistö, S., McCullough, M. L., Miller, A. B., Parker, A. S., … Smith-Warner, S. A. (2008). Fat, protein, and meat consumption and renal cell cancer risk: A pooled analysis of 13 prospective studies. *Journal of the National Cancer Institute*, *100*(23), 1695–1706.

Washio, M., Mori, M., Mikami, K., Miki, T., Watanabe, Y., Nakao, M., Kubo, T., Suzuki, K., Ozasa, K., Wakai, K., & Tamakoshi, A. (2014). Risk factors for renal cell carcinoma in a Japanese population. *Asian Pacific Journal of Cancer Prevention: APJCP*, *15*(21), 9065–9070.

Weng, H., Zeng, X.-T., Li, S., Kwong, J. S. W., Liu, T.-Z., & Wang, X.-H. (2016). Tea Consumption and Risk of Bladder Cancer: A Dose-Response Meta-Analysis. *Frontiers in Physiology*, *7*, 693.

Wolk, A., Lindblad, P., & Adami, H. O. (1996). Nutrition and renal cell cancer. *Cancer Causes & Control: CCC*, *7*(1), 5–18.

Wu, J., Yu, Y., Huang, L., Li, Z., Guo, P., & Xu, Y. W. (2020). Dairy Product Consumption and Bladder Cancer Risk: A Meta-Analysis. *Nutrition and Cancer*, *72*(3), 377–385.

Wu, S., Fisher-Hoch, S. P., Reininger, B. M., Lee, M., & McCormick, J. B. (2019). Fruit and Vegetable Intake is Inversely Associated with Cancer Risk in Mexican-Americans. *Nutrition and Cancer*, *71*(8), 1254–1262.

Xu, X., Zhu, Y., Zheng, X., & Xie, L. (2015). Does beer, wine or liquor consumption correlate with the risk of renal cell carcinoma? A dose-response meta-analysis of prospective cohort studies. *Oncotarget*, *6*(15), 13347–13358.

Yu, E. Y. W., Dai, Y., Wesselius, A., van Osch, F., Brinkman, M., van den Brandt, P., Grant, E. J., White, E., Weiderpass, E., Gunter, M., Hemon, B., & Zeegers, M. P. (2020). Coffee consumption and risk of bladder cancer: A pooled analysis of 501,604 participants from 12 cohort studies in the BLadder Cancer Epidemiology and Nutritional Determinants (BLEND) international study. *European Journal of Epidemiology*, *35*(6), 523–535.

Yu, E. Y. W., Wesselius, A., Sinhart, C., Wolk, A., Stern, M. C., Jiang, X., Tang, L., Marshall, J., Kellen, E., van den Brandt, P., Lu, C.-M., Pohlabeln, H., Steineck, G., Allam, M. F., Karagas, M. R., La Vecchia, C., Porru, S., Carta, A., Golka, K., … Zeegers, M. P. A. (2020). A data mining approach to investigate food groups related to incidence of bladder cancer in the BLadder cancer Epidemiology and Nutritional Determinants International Study. *The British Journal of Nutrition*, *124*(6), 611–619.

Yu, E. Y.-W., Wesselius, A., Mehrkanoon, S., Goosens, M., Brinkman, M., van den Brandt, P., Grant, E. J., White, E., Weiderpass, E., Le Calvez-Kelm, F., Gunter, M. J., Huybrechts, I., Riboli, E., Tjonneland, A., Masala, G., Giles, G. G., Milne, R. L., & Zeegers, M. P. (2021). Vegetable intake and the risk of bladder cancer in the BLadder Cancer Epidemiology and Nutritional Determinants (BLEND) international study. *BMC Medicine*, *19*(1), 56.

Zhou, J., Kelsey, K. T., Giovannucci, E., & Michaud, D. S. (2014). Fluid intake and risk of bladder cancer in the Nurses’ Health Studies. *International Journal of Cancer*, *135*(5), 1229–1237.

Supplemental Table 1：Conversion of 1 serving size to grams^[[1]](#footnote-1)^

| Food groups | Amount |
| --- | --- |
| Vegetables/fruits | 80grams |
| Legumes (soy, bean) | 100grams |
| Fish | 100grams |
| Eggs | 55grams |
| Dairy（milk, yogurt, cheese） | 200grams |
| Red meat (pork, beef) | 85grams |
| Processed meat | 30grams |
| Sugar sweetened beverages | 250ml/grams |

| Supplemental Table 2：General study characteristics of the included studies investigating the association between food groups intake and risk of urological cancer | | | | | | | | | | |
| --- | --- | --- | --- | --- | --- | --- | --- | --- | --- | --- |
| **Study name** | **Author year** | **Sex** | **Age** | **Country** | **Mean follow-up (years)** | **Cases/Participants** | **Main Exposure** | **Outcome** | **Exposure assessment** | **Covariates in fully adjusted model** |
| SMC | Allen 2011 | W | 50-64 | UK | 5.2 | 588/779369 | Tea, coffee, alcohol, milk | Renal cell carcinoma | FFQ | Socioeconomic status, BMI, smoking. |
| SMC | Allen 2009 | W | middle-aged | UK | 7.2 | 1141/1280296 | Alcohol | Renal cell carcinoma | FFQ | Age, region of residence, socioeconomic status, body mass index, smoking, physical activity, use of oral contraceptives, and hormone replacement therapy. |
| ATBC | Bertoia 2010 | M | 50-69 | Finnish | 19 | 255/27062 | Fruit, vegetables | Renal cell carcinoma | FFQ | Age, BMI, education level, measured systolic and diastolic blood pressure, self-reported history of hypertension, leisure-time physical activity, years of smoking, total number of cigarettes per day, trial intervention group , total energy intake and alcohol consumption(g/day). |
| NIH-AARP | Daniel 2012 | M/W | 50-71 | US | 9 | 1816/491841 | Fruits, vegetables | Renal cell carcinoma | FFQ | Age, sex, education, race, marital status, family history of any cancer, BMI, smoking status, hypertension, diabetes. |
| NIH-AARP | Daniel 2013 | M/W | 50-71 | US | 9 | 1816/491842 | Red meat, processed meat, fish | Renal cell carcinoma | FFQ | Age, sex, total energy intake, and mutually adjusted for other types of meat intake, education, marital status, family history of cancer, race, BMI, smoking status, history of diabetes, history of hypertension. |
| California seventh-day Adventists | Fraser 1990 | M/W | 72.2(mean) | US | 6.2 | 14/34198 | Red meat, processed meat | Renal cell carcinoma | FFQ | Age, sex |
| Cancer Prevention Study II | Gapstur 2017 | M/W | 28-94 | US | 30 | 1922/922896  1789/922896 | Coffee | Renal cell carcinoma  Bladder cancer | FFQ | Age, sex, smoking variables, as well as dummy variables for race, marital status, education, alcohol consumption, body mass index, physical activity, family history of cancer, red and processed meat/vegetable intake, and current tea drinking. |
| NIH-AARP | George 2009 | M/W | ≥50 | US | 9 | 363/483338  258/483338 | Fruits，vegetables | Renal cell carcinoma  Bladder cancer | FFQ | Adjusted for age, smoking, energy intake (log-transformed kcal), BMI, alcohol, physical activity, education, race, marital status, family history,  menopausal hormone therapy, and vegetable intake |
| EPIC | Jay 2017 | M/W | 35-70 | European countries | 11.5 | 931/477325 | Alcohol | Renal cell carcinoma | FFQ | Sex, BMI, smoking status, hypertension, for type of alcoholic beverage, models were additionally adjusted for other alcoholic beverages. |
| EPIC | Heath 2021 | M/W | 52（median ） | European countries | 15 | 888/389220 | Soft drink | Renal cell carcinoma | FFQ | age at recruitment , educational attainment, smoking status, alcohol consumption, physical  activity, juice intake, and total soft drink intake，body mass index and total energy intake |
| PLCO | Hashibe 2015 | M/W | 50-64 | US | 10 | 318/97334  398/97334 | Coffee | Renal cell carcinoma  Bladder cancer | FFQ | Age, gender, race, education, smoking, alcohol. |
| ATBC | Hashmian 2019 | M/W | 50-69 | Finland | 17.6 | 835/29133  366/29133 | Coffee, tea | Renal cell carcinoma  Bladder cancer | FFQ | Age, BMI, level of education, years of smoking, cigarettes smoked per day, alcohol, history of diabetes, frequency of physical activity in leisure time, Energy. |
| PLCO | Karami 2015 | M/W | 55-74 | US | 11.4 | 408/107998 | Alcohol | Renal cell carcinoma | FFQ | Sex, race, hypertension, BMI, smoking status and study center. |
| NHS, HPFS | Lee 2006 | M/W | W:30-55 M: 40-75 | US | NHS:20 HPFS:14 | W:132/121700 M:116/51529 | Tea, coffee, alcohol, milk | Renal cell carcinoma | FFQ | BMI, history of hypertension, parity, history of diabetes, smoking status, total energy intake. |
| NHS, HPFS | Lee 2006 | M/W | W:30-55 M: 40-75 | US | NHS:20 HPFS:14 | W:132/121700 M:116/51529 | Fruits, vegetables | Renal cell carcinoma | FFQ | BMI, history of hypertension, parity, history of diabetes, smoking status, total energy intake. |
| VIP | Nilsson 2010 | M/W | 40-60 | US | 6 | 56/64603 | Coffee | Renal cell carcinoma | FFQ | Age, sex, BMI, smoking, education, and recreational physical activity. |
| JPHC | Leung 2021 | M/W | 40-69 | Japan | 15.8 | 169/73024  297/73024 | Sugary drink | Renal cell carcinoma  Bladder cancer | FFQ | Age, public health center, body mass index history of hypertension, history of diabetes, smoking status and intensity, physical activities height, and intake of total energy. |
| NIH-AARP | Lew 2011 | M/W | 50-71 | US | 9 | 1814/491841 | Alcohol | Renal cell carcinoma | FFQ | Age, race, body mass index, marital status, education, vigorous physical activity, smoking, history of hypertension, and intakes of protein and total energy excluding energy from alcohol. |
| The NOWAC Study, the NSHDS study | Lukic 2018 | M/W | 30-70 | Scandinavian countries | 13.6 | 475/193439  479/193439 | Coffee | Renal cell carcinoma  Bladder cancer | FFQ | Smoking status, body mass index, sex. |
| VITAL | Macleod 2013 | M | 50-76 | European countries | 8 | 249/77260 | Alcohol, fruits, vegetables | Renal cell carcinoma | FFQ | Age, BMI, race, years of smoking, gender hypertension, diabetes race. |
| ATBC | Mahabir 2003 | M | 50-69 | Finland | 6.1 | 195/27111 | Alcohol | Renal cell carcinoma | FFQ | Age, BMI, supplement group, calories, blood pressure, years of smoking regularly, total number of cigarettes smoked per day, and fruits and vegetables. |
| JPHC | Minami 2021 | M/W | 40-69 | Japan | 19 | 340/105663 | Alcohol | Renal cell carcinoma | FFQ | Age, public health center area, BMI, medical history, tobacco smoking. |
| The Iowa Women’s Health Study | Nicodemus 2004 | W | 55-69 | Iowa | 15 | 124/34637 | Alcohol, fruits, vegetables | Renal cell carcinoma | FFQ | Age |
| SMC | Rashidkhani 2004 | W | 40-76 | Swedish | 14.2 | 254/120237 | Fruits, vegetables | Renal cell carcinoma | FFQ | Age, BMI. |
| SMC | Rashidkhani 2005 | W | 40-76 | Swedish | 14.2 | 132/59237 | Alcohol | Renal cell carcinoma | FFQ | Age, BMI. |
| NIH-AARP | Rhee 2021 | M/W | 50-71 | US | 15 | 2674/420118 | Coffee | Renal cell carcinoma | FFQ | Sex, race, BMI, marriage status, education, smoking, history of diabetes, total energy intake, alcohol consumption |
| EPIC | Rohrmann 2015 | M/W | 35-70 | European countries | 11.8 | 691/477231 | Red meat, processed meat, fish | Renal cell carcinoma | FFQ | Age, center, sex, education, BMI, history of hypertension, smoking status, duration of smoking, energy intake from fat sources, energy intake from non-fat sources, alcohol consumption, fruit consumption, vegetable consumption. |
| Norwegian prospective study | Ursin1990 | M/W | - | Norway | 11 | 42/15914  31/15914 | Milk | Renal cell carcinoma  Bladder cancer | FFQ | Sex, age, and place of residence. |
| NCS | Van Dijk 2005 | M/W | 55-69 | Netherlands | 9.3 | 275/120852 | Fruits, vegetables | Renal cell carcinoma | FFQ | Age, sex, current smoker, number of cigarettes per day, number of smoking years, BMI, history of hypertension and fruit or vegetable consumption for vegetable or fruit consumption; |
| JACC | Washio 2005 | W/M | ≥40 | Japan | 10 | 48/114517 | Red meat, coffee, milk | Renal cell carcinoma | FFQ | Age, sex. |
| ATBC | Wilson 2009 | M | 50-69 | Finland | 15.2 | 228/27111 | Tea, alcohol, fish, fruits | Renal cell carcinoma | FFQ | Age, body mass index, pack-years of smoking, systolic blood pressure, alcohol intake, leisure time physical activity, urban residence, educational level, All dietary intake variables were energy adjusted. |
| SMC | Wolk 2006 | W | 40-76 | Swedish | 15.3 | 150/61433 | Fish | Renal cell carcinoma | FFQ | Education, body mass index, and intakes of total energy, alcohol, total meat , fruits, vegetables. |
| The Iowa Women's Health Study | Zheng 1996 | W | 55-69 | Iowa | 8 | 61/35369 | Tea | Renal cell carcinoma | FFQ | Age at menarche, age at menopause, age at first pregnancy, and other confounding variables toted In the basic model. |
| EPIC | Botteri 2017 | M/W | 35-71 | European countries | 13.9 | 1802/476160 | Alcohol | Bladder cancer | FFQ | Smoking status, energy intake, body mass index, physical activity and educational level completed. |
| EPIC | Buchner 2009 | M/W | 25-70 | European countries | 8.7 | 1015/478533 | Fruits, vegetables | Bladder cancer | FFQ | Age at recruitment, gender and center and adjusted for smoking status, duration of smoking, lifetime number. |
| The Japan-Hawaii Cancer Study | Chyou 1993 | M | - | Hawaii | 22 | 96/7995 | Alcohol, fish, processed meat, milk, fruit, coffee, tea | Bladder cancer | FFQ | Age, smoking. |
| The Framingham heart study | Djousse 2004 | M/W | 50-70 | US | 27.3 | 126/10125 | Alcohol | Bladder cancer | FFQ | Age, sex, cohort, smoking status, and pack-years of cigarette smoking; for beverage specific analyses, also controlled for other beverage types. |
| NIH-AARP | Ferrucci 2010 | M/W | 20-71 | US | 7 | 854/300933 | Red meat, processed meat | Bladder cancer | FFQ | Age, gender, smoking, and intakes of fruit, vegetables, beverages, and total energy. |
| - | Heilbrun 1986 | M | 45-68 | Japan | 3 | 57/7833 | Tea | Bladder cancer | FFQ | Age at examination and pack-yrs of smoking. |
| NHS | Holick 2005 | W | W:30-55 | US | 20 | 237/88976 | Fruits, vegetables | Bladder cancer | FFQ | Age, pack-years of cigarette smoking, current smoking, and total caloric intake. |
| NHS,HPFS | Holick 2006 | M/W | W:30-55 M: 40-75 | US | NHS:20 HPFS:14 | W:235/121700 M:501/51529 | Fish | Bladder cancer | FFQ | Age, total caloric intake, pack-years of cigarette smoking, and current smoking. |
| EPIC | Jakszyn 2011 | M/W | 35-70 | Europen couns | 8.7 | 1001/481419 | Red meat | Bladder cancer | FFQ | Age at recruitment, sex, and center and adjusted for educational level, BMI (as continuous variable), smoking status, lifetime intensity of smoking (number of cigarettes per day), time since quitting or duration of smoking, and total energy intake. |
| NCS | Keszei 2010 | M/W | 55-69 | Netherlands | 16.3 | 1549/120852 | Dairy products | Bladder cancer | FFQ | Age，sex, additionally, for current cigarette smoking status, years of smoking, number of cigarettes smoked, and intakes of vegetables, fruits, meat, beverages, energy, and fat. |
| JPHC | Kurahashi 2009 | M/W | 40-69 | Japan | 15 | 206/104440 | Coffee, tea | Bladder cancer | FFQ | Age, area, smoking status, and alcohol drinking |
| SWC | Larsson 2008 | M/W | 40-76 | Swedish | 15 | 485/82002 | Fruits, vegetables | Bladder cancer | FFQ | Age, sex, education, smoking status, and total energy intake. |
| SMC | Larsson 2008 | M/W | 45-83 | Swedish | 15 | 485/82002 | Dairy products | Bladder cancer |  | Age, sex, education, cigarette smoking, and total energy intake. |
| SWC | Larsson 2009 | M/W | 40-76 | Swedish | 15.3 | 485/82002 | Red meat, processed meat, fish | Bladder cancer | FFQ | Age, sex, education, smoking status, pack-years of smoking, and total energy intake |
| NIH-AARP | Loftfield 2017 | M/W | 50-71 | US | 6.3 | 6012/469047 | Coffee | Bladder cancer | FFQ | Age at study baseline, sex, smoking, race/ethnicity, BMI, level of education ， alcohol consumption , self-reported general health status, total energy intake, nutrient density-adjusted fruit intake, nutrient-density-adjusted vegetable intake, supplement use , physical activity, history of diabetes , and family history of cancer . |
| JPHC | Masaoka 2017 | M/W | 40-69 | Japan | 18.2 | 464/95915 | Alcohol | Bladder cancer | FFQ | Age, sex, area and smoking. |
| NHS,HPFS | Michaud 2006 | M/W | W:30-55 M: 40-75 | US | NHS:20 HPFS:14 | 808/173229 | Processed meat | Bladder cancer | FFQ | Sex, age, caloric intake, and pack-years of smoking and for geographic region and total fluid intake. |
| ATBC | Michaud 2002 | M | 50-69 | Finland | 11 | 344/27111 | Fruits, vegetables | Bladder cancer | FFQ | Age, duration of smoking, smoking dose, total energy, and trial interventions. |
| HPFS | Michaud 1999 | M | 40-75 | US | 14 | 252/47909 | Fruits, vegetables | Bladder cancer | FFQ | Age, pack-years of cigarette smoking, current smoking status, geographic region, total fluid intake, and caloric intake. |
| HPFS | Michaud 1999 | M | 40-75 | US | 10 | 252/47909 | Coffee, tea, alcohol | Bladder cancer | FFQ | Geographic region, age, smoking status, energy intake, intake of fruits and vegetables. |
| The Adventist health Study | Mills 1991 | M/W | ≥25 | US | 8 | 52/34198 | Alcohol, coffee, vegetables, meat | Bladder cancer | FFQ | Age, sex, smoking. |
| ATOMIC-BOMB SURVIVORS | Nagano 2001 | M/W | M:52.8.W:56.8 | Japan | 13 | 122/38540 | Tea | Bladder cancer | FFQ | Age, gender, radiation dose, smoking status, drinking history, BMI, education level, and calendar time. |
| ATOMIC-BOMB SURVIVORS | Nagano 2000 | M/W | M:52.8.W:56.8 | Japan | 13 | 114/38540 | Fruits, vegetables, meat, processed meat, tea, coffee | Bladder cancer | FFQ | Adjusted for age, gender, radiation dose, smoking status, education level, body-mass index, and calendar time. |
| JPHC | Narii 2022 | M/W | 40-69 | Japan | 5 | 401/80952 | Fruits, vegetables | Bladder cancer | FFQ | Age, area, BMI, smoking status, history of diabetes mellitus, family history of any cancers, Alcohol intake, coffee intake, physical activity, fish intake, red meat intake and supplement intake. |
| NIH-AARP | Nguyen 2021 | M/W | 50-71 | US | 6.3 | 8567/515628 | Cruciferous vegetables | Bladder cancer | FFQ | - |
| VIP | Nilsson 2010 | M/W | 40-60 | US | 6 | 118/64603 | Coffee | Bladder cancer | FFQ | Age, sex, BMI, smoking, education, and recreational physical activity. |
| SMC | Park 2013 | W | 45-75 | Hawaii and California. | 12.5 | 581/185885 | Fruits, vegetables | Bladder cancer | FFQ | Age at cohort entry, ethnicity, and total energy intake. |
| EPIC | Ros 2011 | M/W | 25-70 | European countries | 9.3 | 513/233236 | Alcohol, coffee, tea, milk | Bladder cancer | FFQ | Age，sex and center，smoking status. |
| EPIC | Ros 2012 | M/W | 25-71 | European countries | 8.9 | 947/468656 | Fruits, vegetables | Bladder cancer | FFQ | Age at entry, sex and center and adjusted for smoking status, duration of smoking, lifetime intensity of smoking, energy intake from fat and non-fat sources. |
| JACC | Sakauchi 2004 | M/W | ≥40 | Japan | 10 | 88/114517 | Meat, milk, vegetables, fruits | Bladder cancer | FFQ | Sex, Age, and Smoking index. |
| Leisure World Study | Shibata 1992 | M | 65-84 | US | 7 | 71/11580 | Fruits, vegetables | Bladder cancer | FFQ | Age and smoking. |
| CVD screening Participants Study | Stensvold 1994 | M/W | 35-54 | Norway | 10.1 | 53/42973 | Coffee | Bladder cancer | FFQ | Age, residence, and smoking. |
| MCS,OCS | Sugiyama 2017 | M/W | MCS:40-64, OCS:40-79 | Japan | MCS17.6 OCS:13.3 | 274/73346 | Coffee | Bladder cancer | FFQ | Sex, age, BMI, history of hepertension, diabetes mellitus, myocardial infarction, job status, education status, alcohol consumption, and time spent walking. |
| Singapore Chinese Health Study | Sun 2002 | M/W | 45-74 | China | 5 | 61/63257 | Soy | Bladder cancer | FFQ | Age at baseline interview, year of recruitment, sex, and dialect group, cigarette smoking status and level of education. |
| The shanghai Cohort Study | Sun 2004 | M | 45-64 | China | 14.5 | 61/18224 | Soy | Bladder cancer | FFQ | Age, level of education, and cigarette smoking status. |
| The Iowa Women’s Health Study | Tripathi 2002 | W | 55-69 | Iowa | 13 | 112/37459 | Coffee, tea, alcohol | Bladder cancer | FFQ | Age |
| The Takayama study | Wada 2018 | M/W | ≥35 | Japan | 13.6 | 161/30817 | Soy | Bladder cancer | FFQ | Total energy intake by Willet method. |
| PLCO | Xu 2019 | M/W | 55-74 | US | 12.5 | 776/101721 | Red meat white meat processed meat | Bladder cancer | FFQ | Age, sex, race, BMI, education, smoking status, alcohol drinking status, total energy, intake, randomization arm, family history of any cancer, and marital status. |
| PLCO | Xu 2020 | M/W | 50-64 | US | 12.5 | 776/101721 | Dairy products | Bladder cancer | FFQ | Age, sex, race, BMI, education, smoking status, vegetable intake, fruit intake, tea intake, alcohol drinking status, total energy intake, randomization arm, family history of any cancer, and marital status. |
| NCS | Zeegers 2001 | M/W | 55-69 | Netherlands | 6.3 | 569/120852 | Vegetables, Fruits | Bladder cancer | FFQ | Age, sex, number of cigarettes per day, years of cigarette smoking, and total vegetable consumption or total fruit consumption. |
| NCS | Zeegers 2001 | M/W | 55-69 | Netherlands | 6.3 | 569/120852 | Coffee，tea | Bladder cancer | FFQ | Age, sex, number of cigarettes per day, years of cigarette smoking, and total vegetable consumption or total fruit consumption. |

**Abbreviations**

ATBC-Alpha-Tocopherol, Beta-Carotene Cancer Prevention study

BMI-body mass index

EPIC-European Prospective Investigation into Cancer and Nutrition

FFQ-food frequency questionnaire

JACC-Japan Collaborative Cohort Study

JPHC-Japan Public Health based Cohort

HPFS-The Health Professionals Follow-up Study

m-men

NCS-The Netherlands cohort study

NHI-National Health Insurance

NIH-ARRP-NIH-AARP Diet and Health Study

NHS-Nurses’ Health Study

PLCO-The Prostate, Lung, Colorectal, and Ovarian cancer screening trial

RR-relative risk

SMC-The Mammography Cohort Study

VIP-The Vasterbotten Intervention Project

VITAL-The Vitamins and Lifestyle Study

w women

Supplemental Table 3 Newcastle-Ottawa Scale for assessing the quality of studies in meta-Analysis

|  | **Selection** | | | | **Comparability** | **Outcome** | | |  |
| --- | --- | --- | --- | --- | --- | --- | --- | --- | --- |
| Study | Represent activeness  of the exposed cohort | Selection of the non exposed cohort into the study | Ascertainment of exposure | Demonstration that outcome of interest  was not present at start of study | Comparability of cohorts on the basis of the design or analysis | Assessment of outcome | Was follow-up long enough for outcomes to occur | Adequacy of follow up of cohorts | Quality score |
| Allen 2009[1] | * | * | * | * | ** | * | * |  | 8 |
| Allen 2011[2] | * | * | * | * | ** | * | * |  | 8 |
| Bertoia 2010[3] | * | * | * | * | ** | * | * | * | 9 |
| Botteri 2017[4] | * | * | * | * | ** | * | * | * | 9 |
| Buchner 2009[5] | * | * | * | * | ** | * | * |  | 8 |
| Chyou 1993[6] |  | * | * | * | ** | * | * | * | 8 |
| Daniel 2012[7] | * | * | * | * | ** | * | * | * | 9 |
| Daniel 2013[8] | * | * | * | * | ** | * | * | * | 9 |
| Djousse 2004[9] | * | * | * | * | ** | * | * | * | 9 |
| Ferrucci 2010 [10] | * | * | * | * | ** | * | * | * | 9 |
| Fraser 1990[11] | * | * | * | * | * | * | * |  | 7 |
| Gapstur 2017[12] | * | * | * | * | ** | * | * | * | 9 |
| George 2009[13] | * | * | * | * | ** | * | * | * | 9 |
| Hashibe 2015[14] | * | * | * | * | ** | * | * | * | 9 |
| Hashmian 2019[15] | * | * | * | * | ** | * | * |  | 8 |
| Heilbrun 1986[16] | * | * | * | * | ** | * | * |  | 8 |
| Holick 2005[17] | * | * | * | * | ** | * | * |  | 8 |
| Holick 2006[18] | * | * | * | * | ** | * | * | * | 9 |
| Jakszyn 2011[19] | * | * | * | * | ** | * | * | * | 9 |
| Jay 2017[20] | * | * | * | * | ** | * | * |  | 8 |
| Karami 2015[21] | * | * | * | * | ** | * | * |  | 8 |
| Keszei 2010[22] | * | * | * | * | ** | * | * | * | 9 |
| Kurahashi 2009[23] | * | * | * | * | ** | * | * | * | 9 |
| Larsson 2008[24] | * | * | * | * | ** | * | * | * | 9 |
| Larsson 2008[25] | * | * | * | * | ** | * | * | * | 9 |
| Larsson 2009[26] | * | * | * | * | ** | * | * | * | 9 |
| Lee 2006[27] |  | * | * | * | ** | * | * | * | 8 |
| Lee 2006[28] |  | * | * | * | ** | * | * | * | 8 |
| Leung 2021[29] | * | * | * | * | ** | * | * |  | 8 |
| Lew 2011[30] | * | * | * | * | ** | * | * | * | 9 |
| Loftfield 2017[31] | * | * | * | * | ** | * | * | * | 9 |
| Lukic 2018[32] | * | * | * | * | * | * | * | * | 8 |
| Macleod 2013[33] | * | * | * | * | ** | * | * |  | 8 |
| Mahabir 2003[34] | * | * | * | * | ** | * |  | * | 8 |
| Masaoka 2017[35] | * | * | * | * | ** | * | * | * | 9 |
| Michaud 1999[36] | * | * | * | * | ** | * | * |  | 8 |
| Michaud 1999[37] | * | * | * | * | ** | * | * | * | 9 |
| Michaud 2002[38] | * | * | * | * | ** | * | * |  | 8 |
| Michaud 2006[39] | * | * | * | * | ** | * | * | * | 9 |
| Mills 1991[40] | * | * | * | * | ** | * | * | * | 9 |
| Minami 2021[41] | * | * | * | * | ** | * | * |  | 8 |
| Nagano 2000[42] | * | * | * | * | ** | * | * | * | 9 |
| Nagano 2001[43] | * | * | * | * | ** | * | * | * | 9 |
| Narii 2022[44] | * | * | * | * | ** | * | * | * | 9 |
| Nguyen 2021[45] | * | * | * | * |  | * | * | * | 7 |
| Nicodemus 2004[46] |  |  | * | * | * | * | * |  | 6 |
| Nilsson 2010[47] | * | * | * | * | ** | * | * | * | 9 |
| Park 2013[48] | * | * | * | * | ** | * | * | * | 9 |
| Rashidkhani 2004[49] | * | * | * | * | * | * | * | * | 8 |
| Rashidkhani 2005[50] | * | * | * | * | * | * | * |  | 7 |
| Rhee 2021[51] | * | * | * | * | ** | * | * | * | 9 |
| Rohrmann 2015[52] | * | * | * | * | ** | * | * |  | 8 |
| Ros 2011[53] | * | * | * | * | ** | * | * | * | 9 |
| Ros 2012[54] | * | * | * | * | ** | * | * |  | 8 |
| Sakauchi 2004[55] | * | * | * | * | ** | * | * | * | 9 |
| Shibata 1992[56] |  | * | * | * | ** | * | * | * | 8 |
| Stensvold 1994[57] | * | * | * | * | ** | * | * |  | 7 |
| Sugiyama 2017[58] | * | * | * | * | ** | * | * | * | 9 |
| Sun 2002[59] | * | * | * | * | ** | * | * |  | 8 |
| Sun 2004[60] |  | * | * | * | ** | * | * |  | 7 |
| Tripathi 2002[61] |  | * | * | * | * | * | * | * | 7 |
| Ursin 1990[62] |  | * | * | * | ** | * | * | * | 8 |
| Van Dijk 2005[63] | * | * | * | * | ** | * | * | * | 9 |
| Washio 2005[64] | * | * | * | * | * | * | * |  | 7 |
| Wilson 2009[65] | * | * | * | * | ** | * | * | * | 9 |
| Wolk 2006[66] | * | * | * | * | ** | * | * |  | 8 |
| Xu 2019[67] | * | * | * | * | ** | * | * |  | 8 |
| Xu 2020[68] | * | * | * | * | ** | * | * |  | 8 |
| Zeegers 2001[69] | * | * | * | * | ** | * | * | * | 9 |
| Zeegers 2001[70] | * | * | * | * | ** | * | * | * | 9 |
| Zeegers 2001[71] | * | * | * | * | ** | * | * | * | 9 |
| Zheng 1996[72] |  | * | * | * | ** | * | * | * | 8 |
| Heath 2021[73] | * | * | * | * | ** | * | * | * | 9 |
| Note: A study can be awarded a maximum of one star for each numbered item within the Selection and Outcome categories. A maximum of two stars can be given for Comparability | | | | | | | | | |

**Reference**

[1] N. E. Allen *et al.*, “Moderate Alcohol Intake and Cancer Incidence in Women,” *JNCI Journal of the National Cancer Institute*, vol. 101, no. 5, pp. 296–305, Mar. 2009, doi: 10.1093/jnci/djn514.

[2] N. E. Allen, A. Balkwill, V. Beral, J. Green, G. Reeves, and Million Women Study Collaborators, “Fluid intake and incidence of renal cell carcinoma in UK women,” *Br J Cancer*, vol. 104, no. 9, pp. 1487–1492, Apr. 2011, doi: 10.1038/bjc.2011.90.

[3] M. Bertoia, D. Albanes, S. T. Mayne, S. Männistö, J. Virtamo, and M. E. Wright, “No association between fruit, vegetables, antioxidant nutrients and risk of renal cell carcinoma,” *Int. J. Cancer*, p. NA-NA, 2010, doi: 10.1002/ijc.24829.

[4] E. Botteri *et al.*, “Alcohol consumption and risk of urothelial cell bladder cancer in the European prospective investigation into cancer and nutrition cohort: Alcohol consumption and risk of UCC,” *Int. J. Cancer*, vol. 141, no. 10, pp. 1963–1970, Nov. 2017, doi: 10.1002/ijc.30894.

[5] F. L. Büchner *et al.*, “Consumption of vegetables and fruit and the risk of bladder cancer in the European Prospective Investigation into Cancer and Nutrition,” *Int. J. Cancer*, vol. 125, no. 11, pp. 2643–2651, Dec. 2009, doi: 10.1002/ijc.24582.

[6] P.-H. Chyou, A. M. Y. Nomura, and G. N. Stemmermann, “A prospective study of diet, smoking, and lower urinary tract cancer,” *Annals of Epidemiology*, vol. 3, no. 3, pp. 211–216, May 1993, doi: 10.1016/1047-2797(93)90021-U.

[7] C. R. Daniel *et al.*, “Large prospective investigation of meat intake, related mutagens, and risk of renal cell carcinoma,” *The American Journal of Clinical Nutrition*, vol. 95, no. 1, pp. 155–162, Jan. 2012, doi: 10.3945/ajcn.111.019364.

[8] C. R. Daniel, Y. Park, W.-H. Chow, B. I. Graubard, A. R. Hollenbeck, and R. Sinha, “Intake of fiber and fiber-rich plant foods is associated with a lower risk of renal cell carcinoma in a large US cohort,” *The American Journal of Clinical Nutrition*, vol. 97, no. 5, pp. 1036–1043, May 2013, doi: 10.3945/ajcn.112.045351.

[9] L. Djousse, A. Schatzkin, L. B. Chibnik, R. B. D’Agostino, B. E. Kreger, and R. C. Ellison, “Alcohol Consumption and the Risk of Bladder Cancer in the Framingham Heart Study,” *JNCI Journal of the National Cancer Institute*, vol. 96, no. 18, pp. 1397–1400, Sep. 2004, doi: 10.1093/jnci/djh263.

[10] L. M. Ferrucci *et al.*, “Meat and components of meat and the risk of bladder cancer in the NIH-AARP Diet and Health Study,” *Cancer*, vol. 116, no. 18, pp. 4345–4353, Sep. 2010, doi: 10.1002/cncr.25463.

[11] G. E. Fraser, R. L. Phillips, and W. L. Beeson, “Hypertension, Antihypertensive Medication and Risk of Renal Carcinoma in California Seventh-Day Adventists,” *Int J Epidemiol*, vol. 19, no. 4, pp. 832–838, 1990, doi: 10.1093/ije/19.4.832.

[12] S. M. Gapstur *et al.*, “Associations of Coffee Drinking and Cancer Mortality in the Cancer Prevention Study-II,” *Cancer Epidemiol Biomarkers Prev*, vol. 26, no. 10, pp. 1477–1486, Oct. 2017, doi: 10.1158/1055-9965.EPI-17-0353.

[13] S. M. George *et al.*, “Fruit and vegetable intake and risk of cancer: a prospective cohort study,” *The American Journal of Clinical Nutrition*, vol. 89, no. 1, pp. 347–353, Jan. 2009, doi: 10.3945/ajcn.2008.26722.

[14] M. Hashibe *et al.*, “Coffee, tea, caffeine intake, and the risk of cancer in the PLCO cohort,” *Br J Cancer*, vol. 113, no. 5, pp. 809–816, Sep. 2015, doi: 10.1038/bjc.2015.276.

[15] M. Hashemian *et al.*, “Coffee and tea drinking and risk of cancer of the urinary tract in male smokers,” *Annals of Epidemiology*, vol. 34, pp. 33–39, Jun. 2019, doi: 10.1016/j.annepidem.2019.03.014.

[16] L. K. Heilbrun, A. Nomura, and G. N. Stemmermann, “Black tea consumption and cancer risk: A prospective study,” p. 7.

[17] C. N. Holick, I. De Vivo, D. Feskanich, E. Giovannucci, M. Stampfer, and D. S. Michaud, “Intake of Fruits and Vegetables, Carotenoids, Folate, and Vitamins A, C, E and Risk of Bladder Cancer Among Women (United States),” *Cancer Causes Control*, vol. 16, no. 10, pp. 1135–1145, Dec. 2005, doi: 10.1007/s10552-005-0337-z.

[18] C. N. Holick, E. L. Giovannucci, M. J. Stampfer, and D. S. Michaud, “A prospective study of fish, marine fatty acids, and bladder cancer risk among men and women (United States),” *Cancer Causes Control*, vol. 17, no. 9, pp. 1163–1173, Nov. 2006, doi: 10.1007/s10552-006-0059-x.

[19] P. Jakszyn *et al.*, “Red Meat, Dietary Nitrosamines, and Heme Iron and Risk of Bladder Cancer in the European Prospective Investigation into Cancer and Nutrition (EPIC),” *Cancer Epidemiol Biomarkers Prev*, vol. 20, no. 3, pp. 555–559, Mar. 2011, doi: 10.1158/1055-9965.EPI-10-0971.

[20] M. B. Wozniak *et al.*, “Alcohol consumption and the risk of renal cancers in the European prospective investigation into cancer and nutrition (EPIC): Alcohol consumption and the risk of renal cancers,” *Int. J. Cancer*, vol. 137, no. 8, pp. 1953–1966, Oct. 2015, doi: 10.1002/ijc.29559.

[21] S. Karami, S. E. Daugherty, and M. P. Purdue, “A prospective study of alcohol consumption and renal cell carcinoma risk: Alcohol intake and kidney cancer,” *Int. J. Cancer*, vol. 137, no. 1, pp. 238–242, Jul. 2015, doi: 10.1002/ijc.29359.

[22] A. P. Keszei, L. J. Schouten, R. A. Goldbohm, and P. A. van den Brandt, “Dairy Intake and the Risk of Bladder Cancer in the Netherlands Cohort Study on Diet and Cancer,” *American Journal of Epidemiology*, vol. 171, no. 4, pp. 436–446, Feb. 2010, doi: 10.1093/aje/kwp399.

[23] N. Kurahashi, M. Inoue, M. Iwasaki, S. Sasazuki, S. Tsugane, and for the Japan Public Health Center (JPHC) Study Group, “Coffee, green tea, and caffeine consumption and subsequent risk of bladder cancer in relation to smoking status: a prospective study in Japan,” *Cancer Science*, vol. 100, no. 2, pp. 284–291, Feb. 2009, doi: 10.1111/j.1349-7006.2008.01027.x.

[24] S. C. Larsson, S.-O. Andersson, J.-E. Johansson, and A. Wolk, “Cultured milk, yogurt, and dairy intake in relation to bladder cancer risk in a prospective study of Swedish women and men,” *The American Journal of Clinical Nutrition*, vol. 88, no. 4, pp. 1083–1087, Oct. 2008, doi: 10.1093/ajcn/88.4.1083.

[25] S. C. Larsson, S.-O. Andersson, J.-E. Johansson, and A. Wolk, “Fruit and Vegetable Consumption and Risk of Bladder Cancer: A Prospective Cohort Study,” *Cancer Epidemiol Biomarkers Prev*, vol. 17, no. 9, pp. 2519–2522, Sep. 2008, doi: 10.1158/1055-9965.EPI-08-0407.

[26] S. C. Larsson, J.-E. Johansson, S.-O. Andersson, and A. Wolk, “Meat intake and bladder cancer risk in a Swedish prospective cohort,” *Cancer Causes Control*, vol. 20, no. 1, pp. 35–40, Feb. 2009, doi: 10.1007/s10552-008-9214-x.

[27] J. E. Lee, “Total Fluid Intake and Use of Individual Beverages and Risk of Renal Cell Cancer in Two Large Cohorts,” *Cancer Epidemiology Biomarkers & Prevention*, vol. 15, no. 6, pp. 1204–1211, Jun. 2006, doi: 10.1158/1055-9965.EPI-05-0889.

[28] J. E. Lee, E. Giovannucci, S. A. Smith-Warner, D. Spiegelman, W. C. Willett, and G. C. Curhan, “Intakes of Fruits, Vegetables, Vitamins A, C, and E, and Carotenoids and Risk of Renal Cell Cancer,” *Cancer Epidemiol Biomarkers Prev*, vol. 15, no. 12, pp. 2445–2452, Dec. 2006, doi: 10.1158/1055-9965.EPI-06-0553.

[29] C. Y. Leung *et al.*, “Sugary drink consumption and risk of kidney and bladder cancer in Japanese adults,” *Sci Rep*, vol. 11, no. 1, p. 21701, Dec. 2021, doi: 10.1038/s41598-021-01103-x.

[30] J. Q. Lew, W.-H. Chow, A. R. Hollenbeck, A. Schatzkin, and Y. Park, “Alcohol consumption and risk of renal cell cancer: the NIH-AARP diet and health study,” *Br J Cancer*, vol. 104, no. 3, pp. 537–541, Feb. 2011, doi: 10.1038/sj.bjc.6606089.

[31] E. Loftfield, N. D. Freedman, M. Inoue-Choi, B. I. Graubard, and R. Sinha, “A Prospective Investigation of Coffee Drinking and Bladder Cancer Incidence in the United States:,” *Epidemiology*, vol. 28, no. 5, pp. 685–693, Sep. 2017, doi: 10.1097/EDE.0000000000000676.

[32] M. Lukic, L. M. Nilsson, G. Skeie, B. Lindahl, and T. Braaten, “Coffee consumption and risk of rare cancers in Scandinavian countries,” *Eur J Epidemiol*, vol. 33, no. 3, pp. 287–302, Mar. 2018, doi: 10.1007/s10654-018-0369-9.

[33] L. C. Macleod *et al.*, “Risk Factors for Renal Cell Carcinoma in the VITAL Study,” *Journal of Urology*, vol. 190, no. 5, pp. 1657–1661, Nov. 2013, doi: 10.1016/j.juro.2013.04.130.

[34] S. Mahabir *et al.*, “Prospective Study of Alcohol Drinking and Renal Cell Cancer Risk in a Cohort of Finnish Male Smokers,” *Cancer Epidemiol Biomarkers Prev*, p. 7, 2005.

[35] H. Masaoka *et al.*, “Alcohol consumption and bladder cancer risk with or without the flushing response: The Japan Public Health Center-based Prospective Study: Alcohol consumption and bladder cancer,” *Int. J. Cancer*, vol. 141, no. 12, pp. 2480–2488, Dec. 2017, doi: 10.1002/ijc.31028.

[36] D. S. Michaud *et al.*, “Fluid Intake and the Risk of Bladder Cancer in Men,” *N Engl J Med*, vol. 340, no. 18, pp. 1390–1397, May 1999, doi: 10.1056/NEJM199905063401803.

[37] D. S. Michaud, D. Spiegelman, S. K. Clinton, E. B. Rimm, W. C. Willett, and E. L. Giovannucci, “Fruit and Vegetable Intake and Incidence of Bladder Cancer in a Male Prospective Cohort,” *JNCI Journal of the National Cancer Institute*, vol. 91, no. 7, pp. 605–613, Apr. 1999, doi: 10.1093/jnci/91.7.605.

[38] D. S. Michaud, P. Pietinen, P. R. Taylor, M. Virtanen, J. Virtamo, and D. Albanes, “Intakes of fruits and vegetables, carotenoids and vitamins A, E, C in relation to the risk of bladder cancer in the ATBC cohort study,” *Br J Cancer*, vol. 87, no. 9, pp. 960–965, Oct. 2002, doi: 10.1038/sj.bjc.6600604.

[39] D. S. Michaud, C. N. Holick, E. Giovannucci, and M. J. Stampfer, “Meat intake and bladder cancer risk in 2 prospective cohort studies1Ϫ3,” p. 7, 2018.

[40] P. K. Mills, W. L. Beeson, R. L. Phillips, and G. E. Fraser, “Bladder Cancer in a Low Risk Population: Results from the Adventist Health Study,” *American Journal of Epidemiology*, vol. 133, no. 3, pp. 230–239, Feb. 1991, doi: 10.1093/oxfordjournals.aje.a115867.

[41] T. Minami, M. Inoue, N. Sawada, T. Yamaji, M. Iwasaki, and S. Tsugane, “Alcohol consumption, tobacco smoking, and subsequent risk of renal cell carcinoma: The JPHC study,” *Cancer Sci*, vol. 112, no. 12, pp. 5068–5077, Dec. 2021, doi: 10.1111/cas.15129.

[42] J. Nagano, S. Kono, D. L. Preston, and K. Mabuchi, “A prospective study of green tea consumption and cancer incidence, Hiroshima and Nagasaki (Japan),” p. 8.

[43] J. Nagano *et al.*, “Bladder-cancer incidence in relation to vegetable and fruit consumption: A prospective study of atomic-bomb survivors,” *Int. J. Cancer*, vol. 86, no. 1, pp. 132–138, Apr. 2000, doi: 10.1002/(SICI)1097-0215(20000401)86:1<132::AID-IJC21>3.0.CO;2-M.

[44] N. Narii *et al.*, “Vegetable and fruit intake and the risk of bladder cancer: Japan Public Health Center-based prospective study,” *Br J Cancer*, vol. 126, no. 11, pp. 1647–1658, Jun. 2022, doi: 10.1038/s41416-022-01739-0.

[45] T. P. Nguyen, C. A. Zhang, G. A. Sonn, M. L. Eisenberg, and J. D. Brooks, “Consumption of cruciferous vegetables and the risk of bladder cancer in a prospective US cohort: data from the NIH-AARP diet and health study,” p. 10.

[46] K. K. Nicodemus, C. Sweeney, and A. R. Folsom, “Evaluation of dietary, medical and lifestyle risk factors for incident kidney cancer in postmenopausal women,” *Int. J. Cancer*, vol. 108, no. 1, pp. 115–121, Jan. 2004, doi: 10.1002/ijc.11532.

[47] L. M. Nilsson, I. Johansson, P. Lenner, B. Lindahl, and B. Van Guelpen, “Consumption of filtered and boiled coffee and the risk of incident cancer: a prospective cohort study,” *Cancer Causes Control*, vol. 21, no. 10, pp. 1533–1544, Oct. 2010, doi: 10.1007/s10552-010-9582-x.

[48] S.-Y. Park, N. J. Ollberding, C. G. Woolcott, L. R. Wilkens, B. E. Henderson, and L. N. Kolonel, “Fruit and Vegetable Intakes Are Associated with Lower Risk of Bladder Cancer among Women in the Multiethnic Cohort Study,” *The Journal of Nutrition*, vol. 143, no. 8, pp. 1283–1292, Aug. 2013, doi: 10.3945/jn.113.174920.

[49] B. Rashidkhani, P. Lindblad, and A. Wolk, “Fruits, vegetables and risk of renal cell carcinoma: A prospective study of Swedish women,” *Int. J. Cancer*, vol. 113, no. 3, pp. 451–455, Jan. 2005, doi: 10.1002/ijc.20577.

[50] B. Rashidkhani, A. Åkesson, P. Lindblad, and A. Wolk, “Alcohol consumption and risk of renal cell carcinoma: A prospective study of Swedish women,” *Int. J. Cancer*, vol. 117, no. 5, pp. 848–853, Dec. 2005, doi: 10.1002/ijc.21231.

[51] J. Rhee, E. Loftfield, N. D. Freedman, L. M. Liao, R. Sinha, and M. P. Purdue, “Coffee consumption and risk of renal cell carcinoma in the NIH-AARP Diet and Health Study,” *International Journal of Epidemiology*, vol. 50, no. 5, pp. 1473–1481, Nov. 2021, doi: 10.1093/ije/dyab011.

[52] S. Rohrmann *et al.*, “Meat and fish consumption and the risk of renal cell carcinoma in the European prospective investigation into cancer and nutrition: Meat and Fish Consumption and the Risk of Renal Cell Carcinoma,” *Int. J. Cancer*, vol. 136, no. 5, pp. E423–E431, Mar. 2015, doi: 10.1002/ijc.29236.

[53] M. M. Ros *et al.*, “Fluid intake and the risk of urothelial cell carcinomas in the European Prospective Investigation into Cancer and Nutrition (EPIC),” *Int. J. Cancer*, vol. 128, no. 11, pp. 2695–2708, Jun. 2011, doi: 10.1002/ijc.25592.

[54] M. M. Ros *et al.*, “Fruit and vegetable consumption and risk of aggressive and non-aggressive urothelial cell carcinomas in the European Prospective Investigation into Cancer and Nutrition,” *European Journal of Cancer*, vol. 48, no. 17, pp. 3267–3277, Nov. 2012, doi: 10.1016/j.ejca.2012.05.026.

[55] F. Sakauchi *et al.*, “Dietary Habits and Risk of Urothelial Cancer Death in a Large-Scale Cohort Study (JACC Study) in Japan,” *Nutrition and Cancer*, vol. 50, no. 1, p. 8, 2004.

[56] A. Shibata, A. Paganini-Hill, R. Ross, and B. Henderson, “Intake of vegetables, fruits, beta-carotene, vitamin C and vitamin supplements and cancer incidence among the elderly: a prospective study,” *Br J Cancer*, vol. 66, no. 4, pp. 673–679, Oct. 1992, doi: 10.1038/bjc.1992.336.

[57] I. Stensvold and B. K. Jacobsen, “Coffee and cancer: A prospective study of 43,000 Norwegian men and women,” *Cancer Causes Control*, vol. 5, no. 5, pp. 401–408, Sep. 1994, doi: 10.1007/BF01694753.

[58] K. Sugiyama, Y. Sugawara, Y. Tomata, Y. Nishino, A. Fukao, and I. Tsuji, “The association between coffee consumption and bladder cancer incidence in a pooled analysis of the Miyagi Cohort Study and Ohsaki Cohort Study,” *European Journal of Cancer Prevention*, vol. 26, no. 2, pp. 125–130, Mar. 2017, doi: 10.1097/CEJ.0000000000000226.

[59] C.-L. Sun, J.-M. Yuan, K. Arakawa, S.-H. Low, H.-P. Lee, and M. C. Yu, “Dietary soy and increased risk of bladder cancer: the Singapore Chinese Health Study,” *Cancer Epidemiol Biomarkers Prev*, vol. 11, no. 12, pp. 1674–1677, Dec. 2002.

[60] C.-L. Sun, J.-M. Yuan, X.-L. Wang, Y.-T. Gao, R. K. Ross, and M. C. Yu, “Dietary soy and increased risk of bladder cancer: A prospective cohort study of men in Shanghai, China,” *Int. J. Cancer*, vol. 112, no. 2, pp. 319–323, Nov. 2004, doi: 10.1002/ijc.20384.

[61] A. Tripathi, A. R. Folsom, and K. E. Anderson, “Risk factors for urinary bladder carcinoma in postmenopausal women: The Iowa Women’s Health Study,” *Cancer*, vol. 95, no. 11, pp. 2316–2323, Dec. 2002, doi: 10.1002/cncr.10975.

[62] G. Ursin, E. Bjelke, I. Heuch, and S. Vollset, “Milk consumption and cancer incidence: a Norwegian prospective study,” *Br J Cancer*, vol. 61, no. 3, pp. 456–459, Mar. 1990, doi: 10.1038/bjc.1990.100.

[63] B. A. C. van Dijk, L. J. Schouten, L. A. L. M. Kiemeney, R. A. Goldbohm, and P. A. van den Brandt, “Vegetable and fruit consumption and risk of renal cell carcinoma: Results from the Netherlands cohort study,” *Int. J. Cancer*, vol. 117, no. 4, pp. 648–654, Nov. 2005, doi: 10.1002/ijc.21203.

[64] M. Washio *et al.*, “Risk Factors for Kidney Cancer in a Japanese Population: Findings from the JACC Study,” *Journal of Epidemiology*, vol. 15, no. Supplement_II, pp. S203–S211, 2005, doi: 10.2188/jea.15.S203.

[65] R. T. Wilson *et al.*, “Fish, Vitamin D, and Flavonoids in Relation to Renal Cell Cancer Among Smokers,” *American Journal of Epidemiology*, vol. 170, no. 6, pp. 717–729, Sep. 2009, doi: 10.1093/aje/kwp178.

[66] A. Wolk, S. C. Larsson, J.-E. Johansson, and P. Ekman, “Long-term Fatty Fish Consumption and Renal Cell Carcinoma Incidence in Women,” p. 6.

[67] X. Xu, “Processed Meat Intake and Bladder Cancer Risk in the Prostate, Lung, Colorectal, and Ovarian (PLCO) Cohort,” *Cancer Epidemiol Biomarkers Prev*, vol. 28, no. 12, pp. 1993–1997, Dec. 2019, doi: 10.1158/1055-9965.EPI-19-0604.

[68] X. Xu, “Dairy Product Consumption and Bladder Cancer Risk in the Prostate, Lung, Colorectal, and Ovarian (PLCO) Cohort,” *Front. Nutr.*, vol. 7, p. 97, Jul. 2020, doi: 10.3389/fnut.2020.00097.

[69] M. P. Zeegers, E. Dorant, R. A. Goldbohm, and P. A. van den Brandt, “Are coffee, tea, and total fluid consumption associated with bladder cancer risk? Results from the Netherlands Cohort Study,” *Cancer Causes Control*, vol. 12, no. 3, pp. 231–238, Apr. 2001, doi: 10.1023/a:1011245627593.

[70] Zeegers, M. P., Goldbohm, R. A., & van den Brandt, P. A. (2001). Consumption of vegetables and fruits and urothelial cancer incidence: a prospective study. *Cancer epidemiology, biomarkers & prevention : a publication of the American Association for Cancer Research, cosponsored by the American Society of Preventive Oncology*, *10*(11), 1121–1128.

[71] M. P. A. Zeegers, A. Volovics, E. Dorant, R. Alexandra Goldbohm, and P. A. van den Brandt, “Alcohol Consumption and Bladder Cancer Risk: Results from the Netherlands Cohort Study,” *American Journal of Epidemiology*, vol. 153, no. 1, pp. 38–41, Jan. 2001, doi: 10.1093/aje/153.1.38.

[72] W. Zheng, T. J. Doyle, L. H. Kushi, T. A. Sellers, C.-P. Hong, and A. R. Folsom, “Tea Consumption and Cancer Incidence in a Prospective Cohort Study of Postmenopausal Women,” *American Journal of Epidemiology*, vol. 144, no. 2, pp. 175–182, Jul. 1996, doi: 10.1093/oxfordjournals.aje.a008905.

[73] Heath AK, Clasen JL, Jayanth NP, Jenab M, Tjønneland A, Petersen KEN, Overvad K, Srour B, Katzke V, Bergmann MM, et al. Soft Drink and Juice Consumption and Renal Cell Carcinoma Incidence and Mortality in the European Prospective Investigation into Cancer and Nutrition. Cancer Epidemiology, Biomarkers & Prevention 2021;30:1270–4.

Supplemental Table 4 High vs. low intake meta-analysis for fish and bladder cancer, stratified by sex, follow-up, geographic location, and number of participants

| **Dietary factor** | **No of studies** | **RR** | **95%CI** | **I^2^(%)** | **P for difference among I-squares** |
| --- | --- | --- | --- | --- | --- |
| **Sex** |  |  |  |  |  |
| Men | 1 | 0.67 | 0.26,1.70 | NA | 0.712 |
| Women | 0 | NA | NA | NA |  |
| Men and women | 4 | 0.81 | 0.55,1.20 | 67.8 |  |
| **Follow-up** |  |  |  |  |  |
| ≥10years | 5 | 0.79 | 0.56,1.11 | 57.4 | NA |
| <10years | 0 | NA | NA | NA |  |
| **Geographic location** |  |  |  |  |  |
| America | 2 | 0.55 | 0.41,0.75 | 0 | 0.044 |
| Europe | 1 | 0.92 | 0.65,1.30 | NA |  |
| Asia | 2 | 1.01 | 0.57,1.79 | 42.2 |  |
| **Number of participants** |  |  |  |  |  |
| ≥100,000 | 2 | 0.57 | 0.43,0.76 | 0.0 | 0.008 |
| <100,000 | 3 | 0.98 | 0.74,1.30 | 0.0 |  |

I, Inconsistency; NA, not applicable; RR, risk ratio

Supplemental Table 5 High vs. low intake meta-analysis for coffee and bladder cancer, stratified by sex, follow-up, geographic location, and number of participants

| **Dietary factor** | **No of studies** | **RR** | **95%CI** | **I^2^(%)** | **P for difference among I-squares** |
| --- | --- | --- | --- | --- | --- |
| **Sex** |  |  |  |  |  |
| Men | 3 | 1.08 | 0.73,1.59 | 42.9 | 0.249 |
| Women | 1 | 1.59 | 0.95,2.67 | NA |  |
| Men and women | 12 | 1.00 | 0.83,1.20 | 66.3 |  |
| **Follow-up** |  |  |  |  |  |
| ≥10years | 11 | 0.96 | 0.76,1.20 | 69.1 | 0.196 |
| <10years | 5 | 1.14 | 1.00,1.29 | 0 |  |
| **Geographic location** |  |  |  |  |  |
| America | 8 | 1.09 | 0.92,1.28 | 39.1 | 0.020 |
| Europe | 5 | 1.19 | 1.01,1.39 | 0 |  |
| Asia | 3 | 0.57 | 0.35,0.93 | 62.8 |  |
| **Number of participants** |  |  |  |  |  |
| ≥100,000 | 6 | 0.98 | 0.76,1.25 | 79.6 | 0.497 |
| <100,000 | 10 | 1.09 | 0.89,1.34 | 35.9 |  |

I, Inconsistency; NA, not applicable; RR, risk ratio

Supplemental Table 6 High vs. low intake meta-analysis for fruit and bladder cancer, stratified by sex, follow-up, geographic location, and number of participants

| **Dietary factor** | **No of studies** | **RR** | **95%CI** | **I^2^(%)** | **P for difference among I-squares** |
| --- | --- | --- | --- | --- | --- |
| **Sex** |  |  |  |  |  |
| Men | 4 | 0.87 | 0.62,1.22 | 46.1 | 0.745 |
| Women | 2 | 0.75 | 0.52,1.08 | 56.7 |  |
| Men and women | 7 | 0.88 | 0.72,1.08 | 62.8 |  |
| **Follow-up** |  |  |  |  |  |
| ≥10years | 8 | 0.80 | 0.65,0.98 | 51.7 | 0.292 |
| <10years | 5 | 0.94 | 0.76,1.17 | 61.1 |  |
| **Geographic location** |  |  |  |  |  |
| America | 5 | 0.76 | 0.59,0.97 | 38 | 0.276 |
| Europe | 5 | 0.94 | 0.83,1.06 | 0 |  |
| Asia | 3 | 0.75 | 0.37,1.51 | 85.3 |  |
| **Number of participants** |  |  |  |  |  |
| ≥100,000 | 6 | 0.80 | 0.66,0.98 | 58.9 | 0.413 |
| <100,000 | 7 | 0.91 | 0.72,1.16 | 58.1 |  |

I, Inconsistency; NA, not applicable; RR, risk ratio

Supplemental Table 7 High vs. low intake meta-analysis for vegetable and bladder cancer, stratified by sex, follow-up, geographic location, and number of participants

| **Dietary factor** | **No of studies** | **RR** | **95%CI** | **I^2^(%)** | **P for difference among I-squares** |
| --- | --- | --- | --- | --- | --- |
| **Sex** |  |  |  |  |  |
| Men | 3 | 0.98 | 0.72,1.35 | 36.7 | 0.860 |
| Women | 2 | 0.82 | 0.35,1.93 | 92.5 |  |
| **Men and women** | 7 | 1.02 | 0.92,1.14 | 32 |  |
| Follow-up |  |  |  |  |  |
| ≥10years | 6 | 0.87 | 0.64,1.17 | 75.0 | 0.249 |
| <10years | 6 | 1.04 | 0.94,1.17 | 31.5 |  |
| **Geographic location** |  |  |  |  |  |
| America | 5 | 0.89 | 0.63,1.26 | 86.4 | 0.475 |
| Europe | 5 | 0.95 | 0.85,1.07 | 0 |  |
| Asia | 2 | 1.22 | 0.81,1.83 | 34.7 |  |
| **Number of participants** |  |  |  |  |  |
| ≥100,000 | 7 | 0.92 | 0.75,1.12 | 79.4 | 0.431 |
| <100,000 | 5 | 1.04 | 0.82,1.30 | 49.7 |  |

I, Inconsistency; NA, not applicable; RR, risk ratio

Supplemental Table 8 High vs. low intake meta-analysis for tea and bladder cancer, stratified by sex, follow-up, geographic location, and number of participants

| **Dietary factor** | **No of studies** | **RR** | **95%CI** | **I^2^(%)** | **P for difference among I-squares** |
| --- | --- | --- | --- | --- | --- |
| **Sex** |  |  |  |  |  |
| Men | 4 | 0.70 | 0.41,1.19 | 82 | 0.106 |
| Women | 1 | 0.33 | 0.08,1.36 | NA |  |
| Men and women | 5 | 1.08 | 0.86.1.35 | 42.4 |  |
| **Follow-up** |  |  |  |  |  |
| ≥10years | 7 | 1.03 | 0.82,1.29 | 59 | 0.126 |
| <10years | 3 | 0.43 | 0.15,1.28 | 80.8 |  |
| **Geographic location** |  |  |  |  |  |
| America | 4 | 0.95 | 0.67,1.33 | 54 | 0.789 |
| Europe | 2 | 0.82 | 0.66,1.02 | 0 |  |
| Asia | 4 | 0.83 | 0.42,1.65 | 84.1 |  |
| **Number of participants** |  |  |  |  |  |
| ≥100,000 | 2 | 1.22 | 0.68,2.17 | 80.4 | 0.223 |
| <100,000 | 8 | 0.82 | 0.62,1.08 | 64.9 |  |

I, Inconsistency; NA, not applicable; RR, risk ratio

Supplemental Table 9 High vs. low intake meta-analysis for dairy and bladder cancer, stratified by sex, follow-up, geographic location, and number of participants

| **Dietary factor** | **No of studies** | **RR** | **95%CI** | **I^2^(%)** | **P for difference among I-squares** |
| --- | --- | --- | --- | --- | --- |
| Sex |  |  |  |  |  |
| Men | 1 | 0.67 | 0.46,0.98 | NA | 0.076 |
| Women | 0 | NA | NA | NA |  |
| Men and women | 6 | 0.97 | 0.84,1.10 | 52.8 |  |
| Follow-up |  |  |  |  |  |
| ≥10years | 5 | 0.90 | 0.76,1.06 | 65.2 | 0.309 |
| <10years | 1 | 1.04 | 0.83,1.31 | NA |  |
| Geographic location |  |  |  |  |  |
| America | 2 | 0.87 | 0.56,1.34 | 79.2 | 0.507 |
| Europe | 3 | 1.00 | 0.88,1.14 | 23.2 |  |
| Asia | 2 | 0.73 | 0.41,1.30 | 69.7 |  |
| Number of participants |  |  |  |  |  |
| ≥100,000 | 4 | 0.98 | 0.82,1.17 | 62.0 | 0.218 |
| <100,000 | 3 | 0.84 | 0.71,1.00 | 0 |  |

I, Inconsistency; NA, not applicable; RR, risk ratio


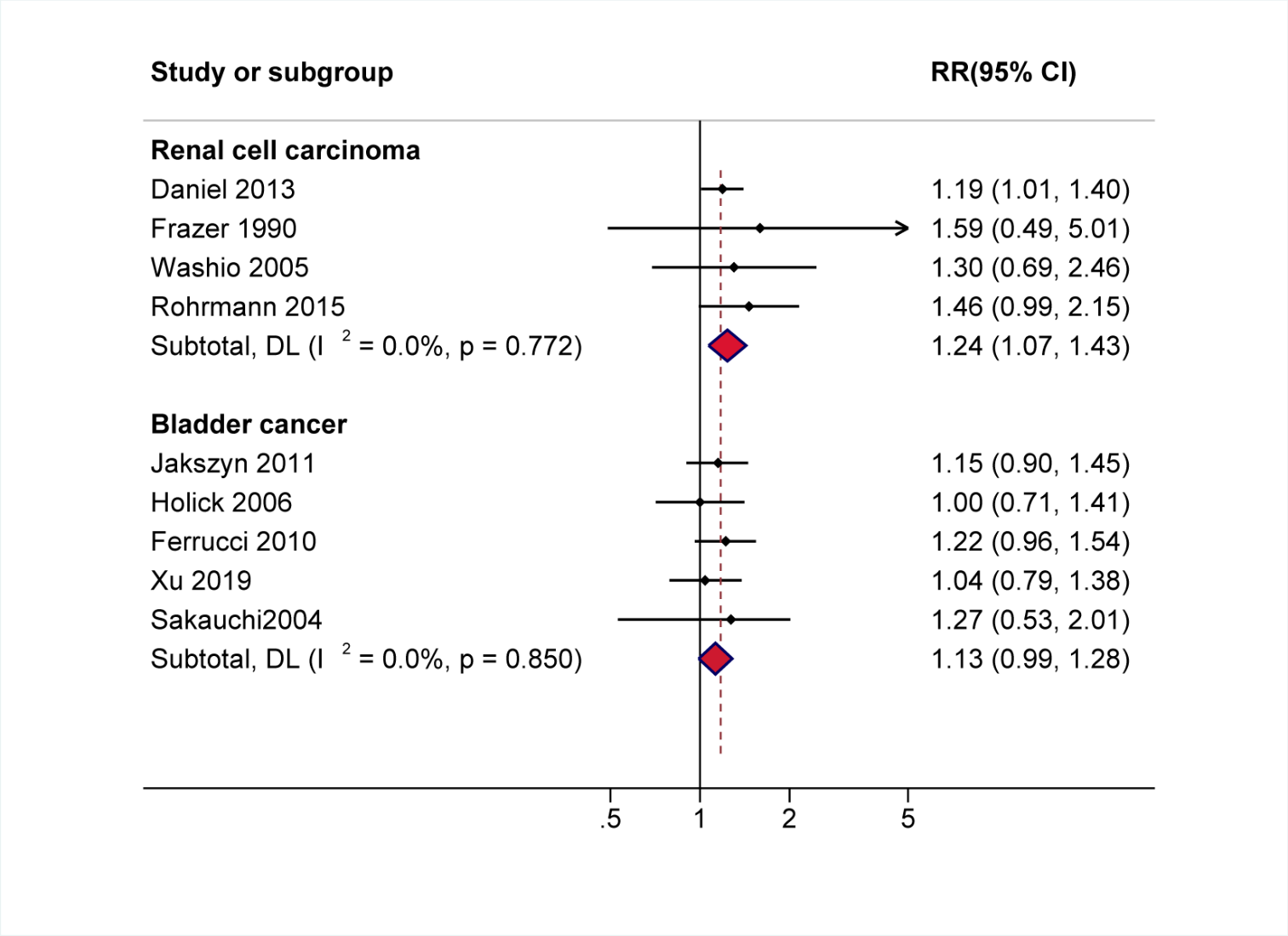
Supplemental Figure 1: Summary of relative risk of bladder cancer, renal cell carcinoma for high versus low red meat intake.95%CI,95%confidence interval


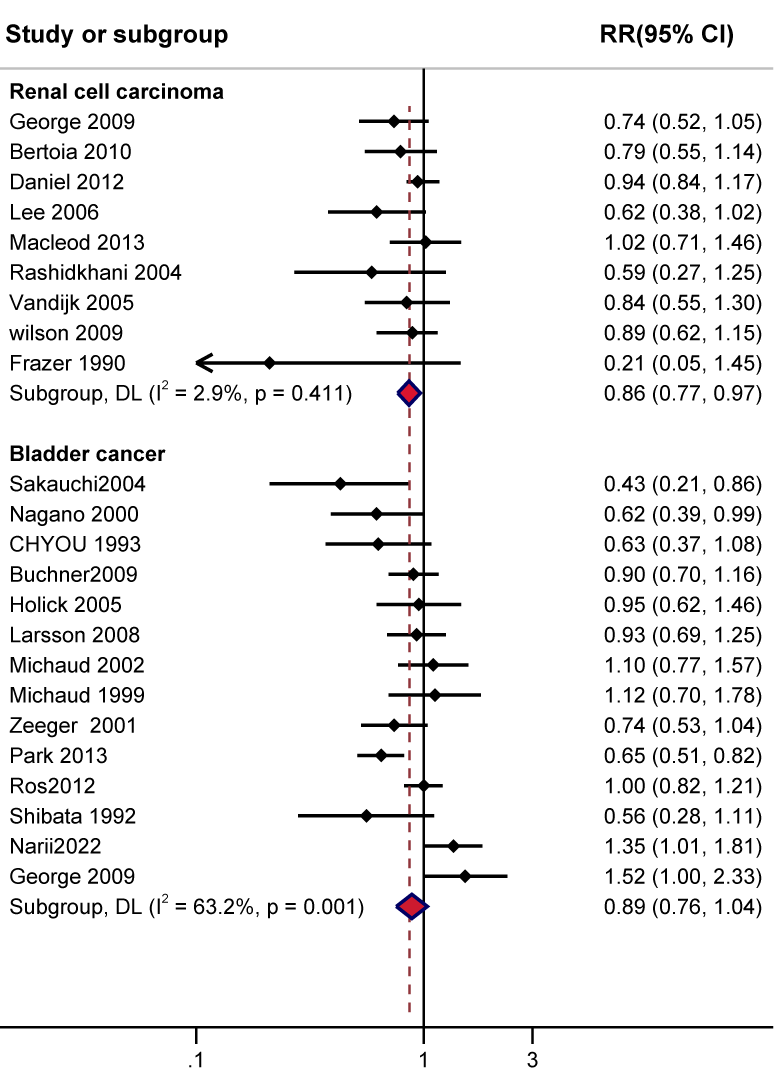


Supplemental Figure 2: Summary of relative risk of Bladder cancer, renal cell carcinoma for high versus low fruit intake.95%CI,95% confidence interval


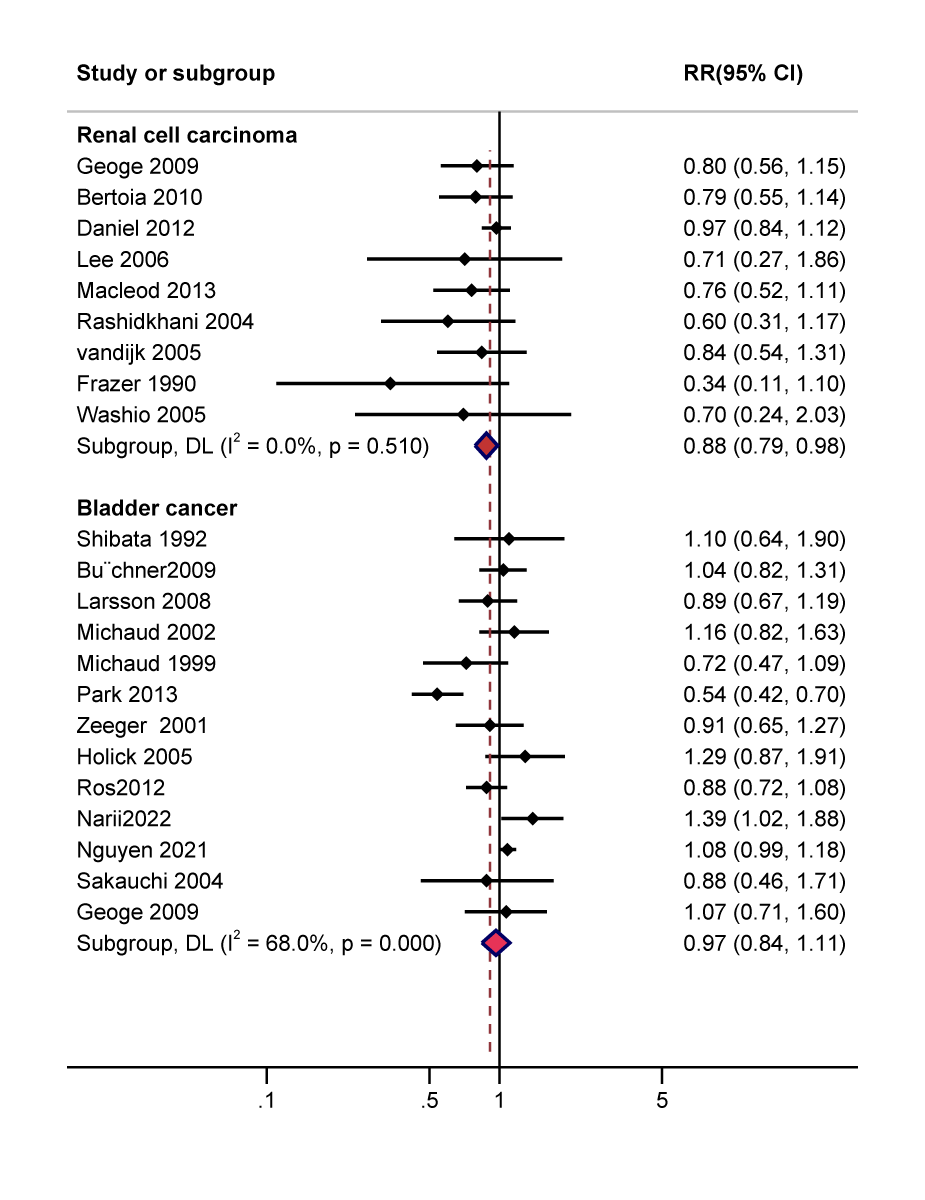


Supplemental Figure 3: Summary of relative risk of bladder cancer, renal cell carcinoma for high versus low vegetables intake.95%CI,95%confidence interval


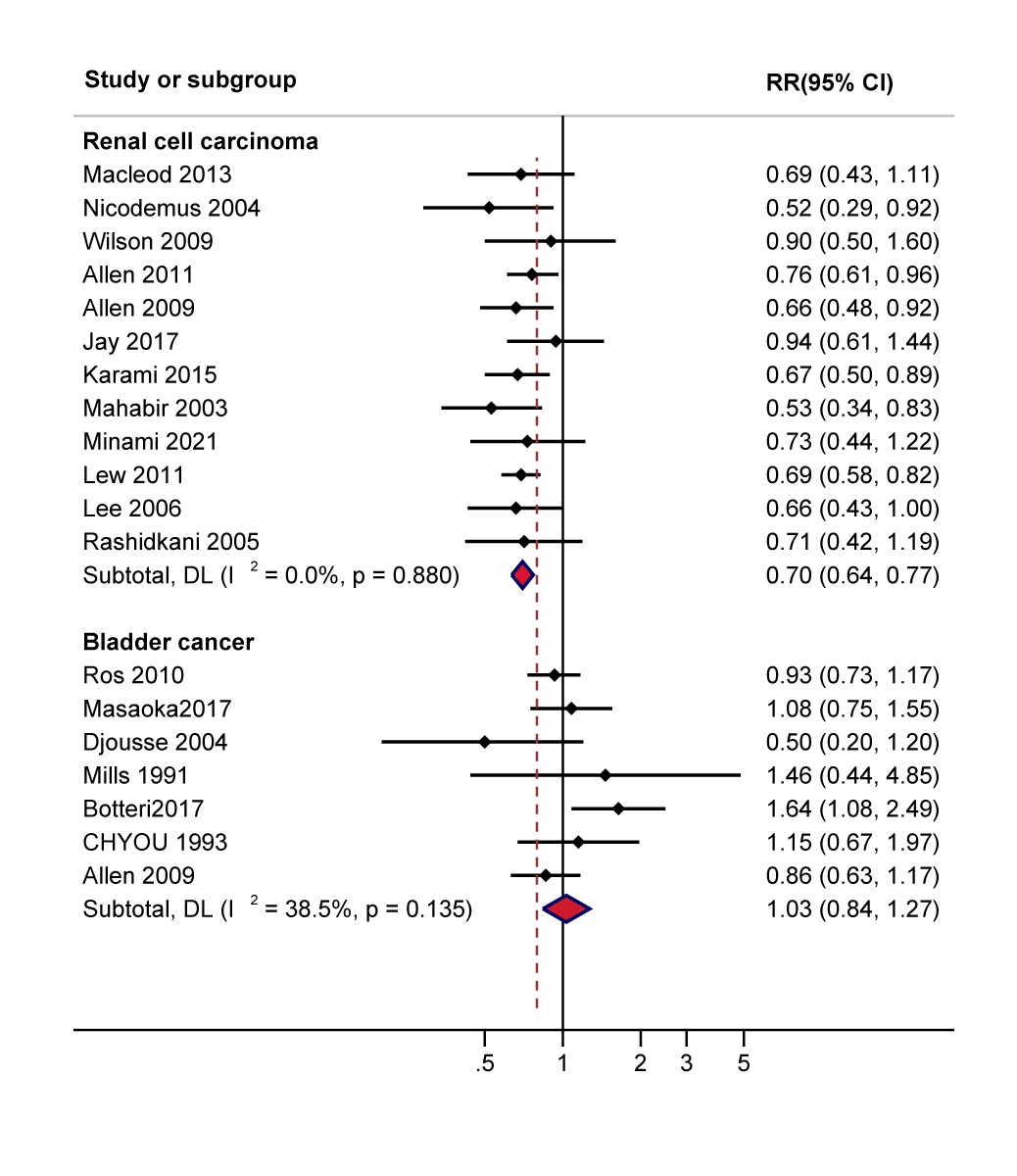


Supplemental Figure 4: Summary of relative risk of bladder cancer, renal cell carcinoma for high versus low alcohol intake.95%CI,95%confidence interval


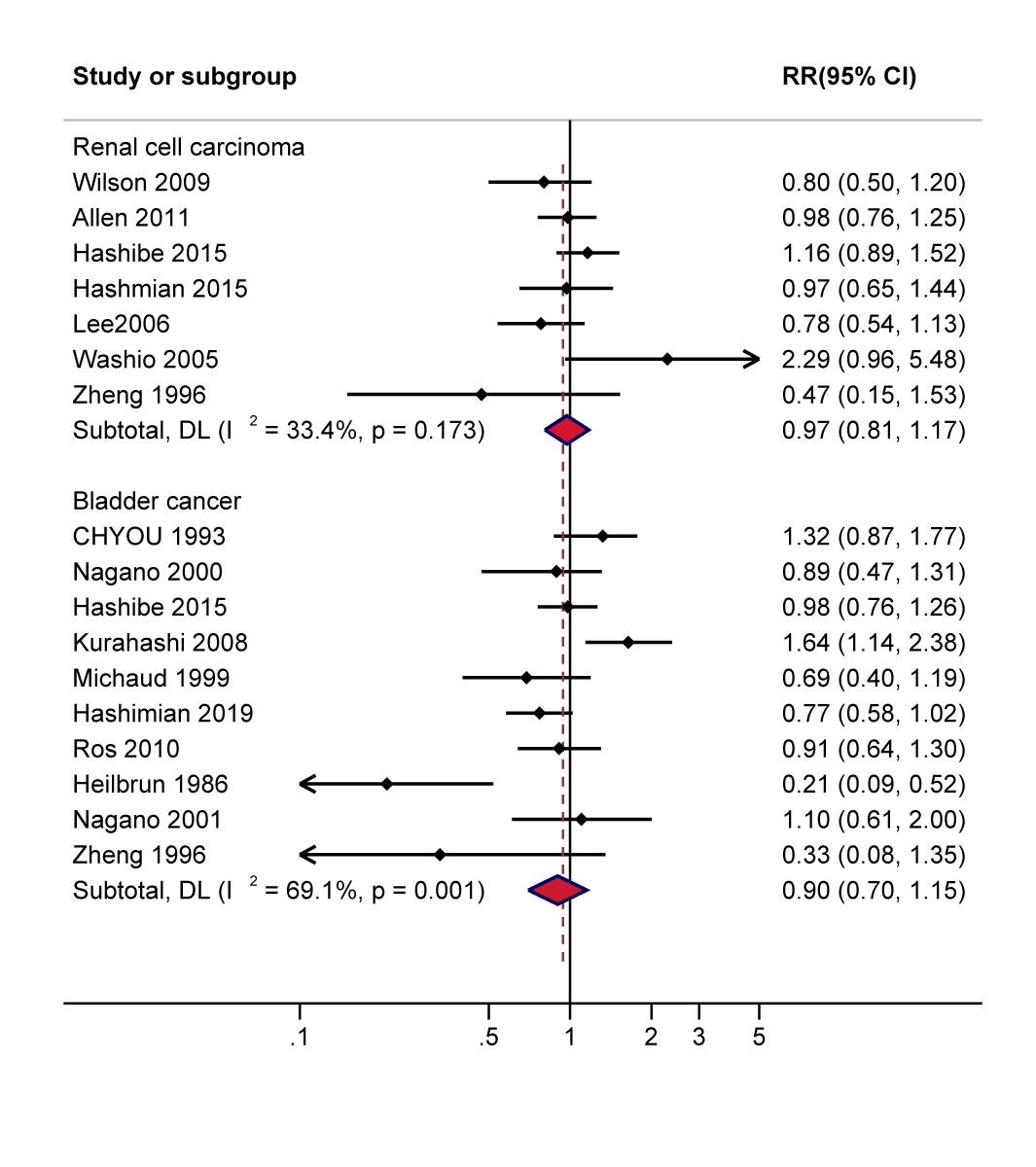


Supplemental Figure 5: Summary of relative risk of bladder cancer, renal cell carcinoma for high versus low tea intake.95%CI,95%confidence interval


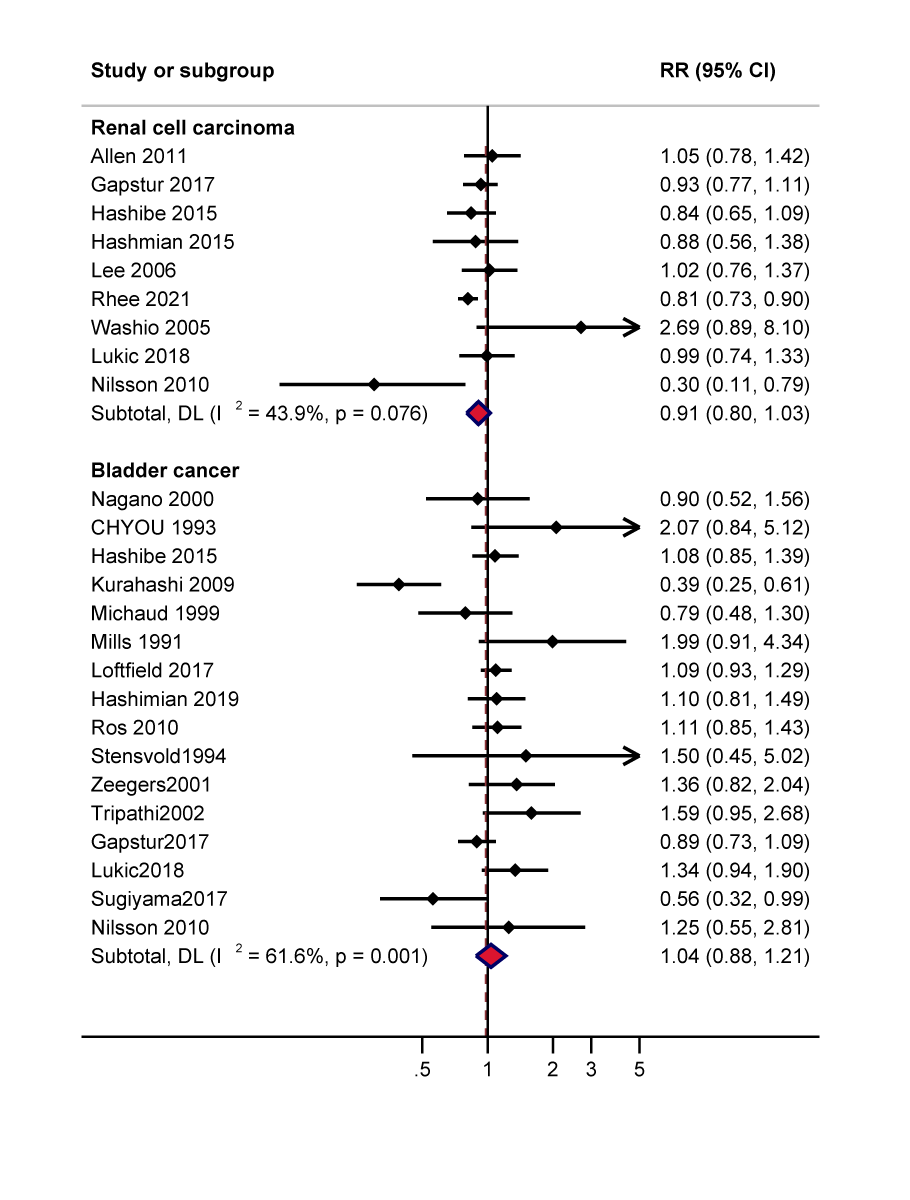
Supplemental Figure 6: Summary of relative risk of bladder cancer, renal cell carcinoma for high versus low coffee intake.95%CI,95%confidence interval


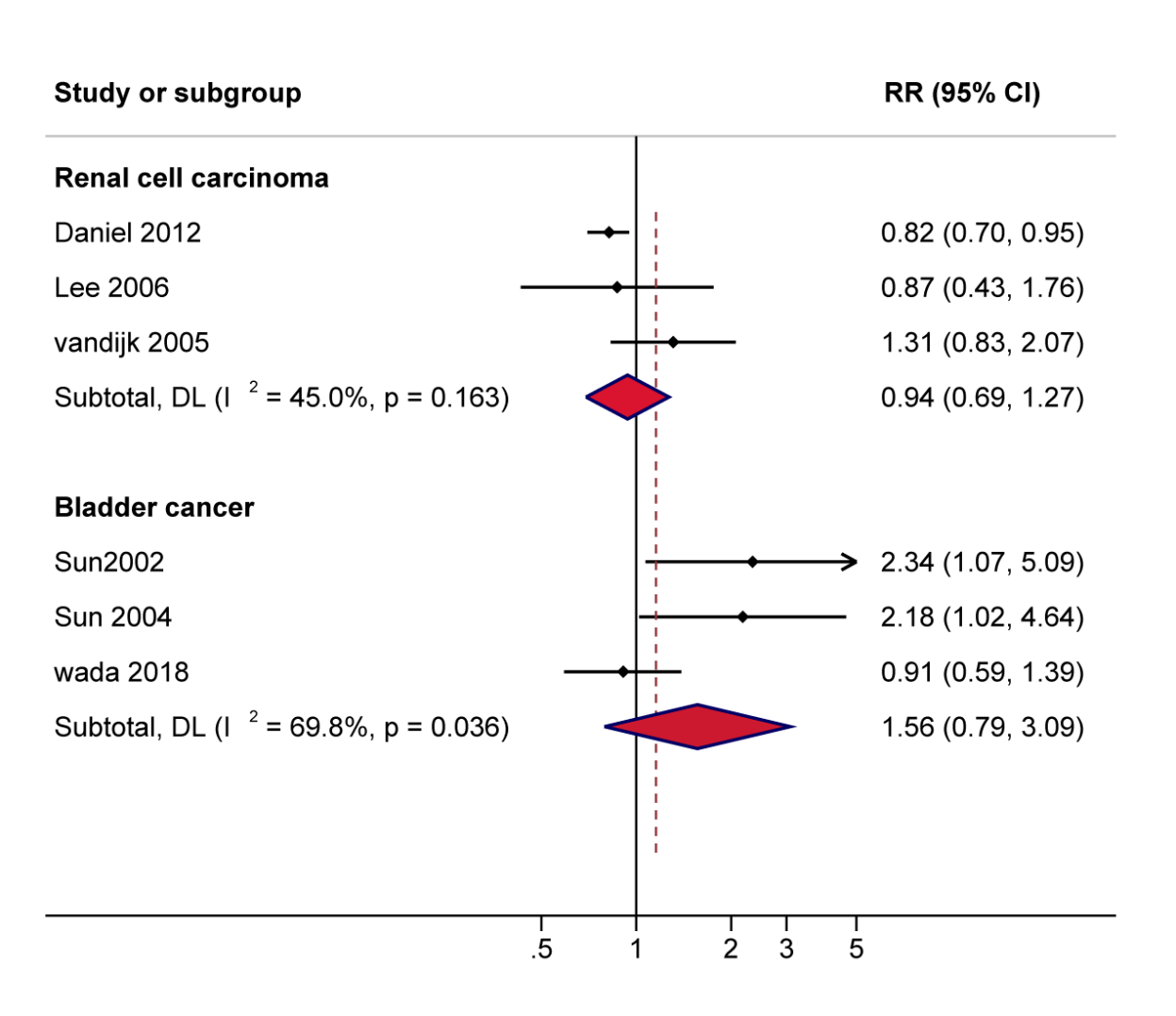
Supplemental Figure 7: Summary of relative risk of bladder cancer, renal cell carcinoma for high versus low legumes intake.95%CI,95%confidence interval


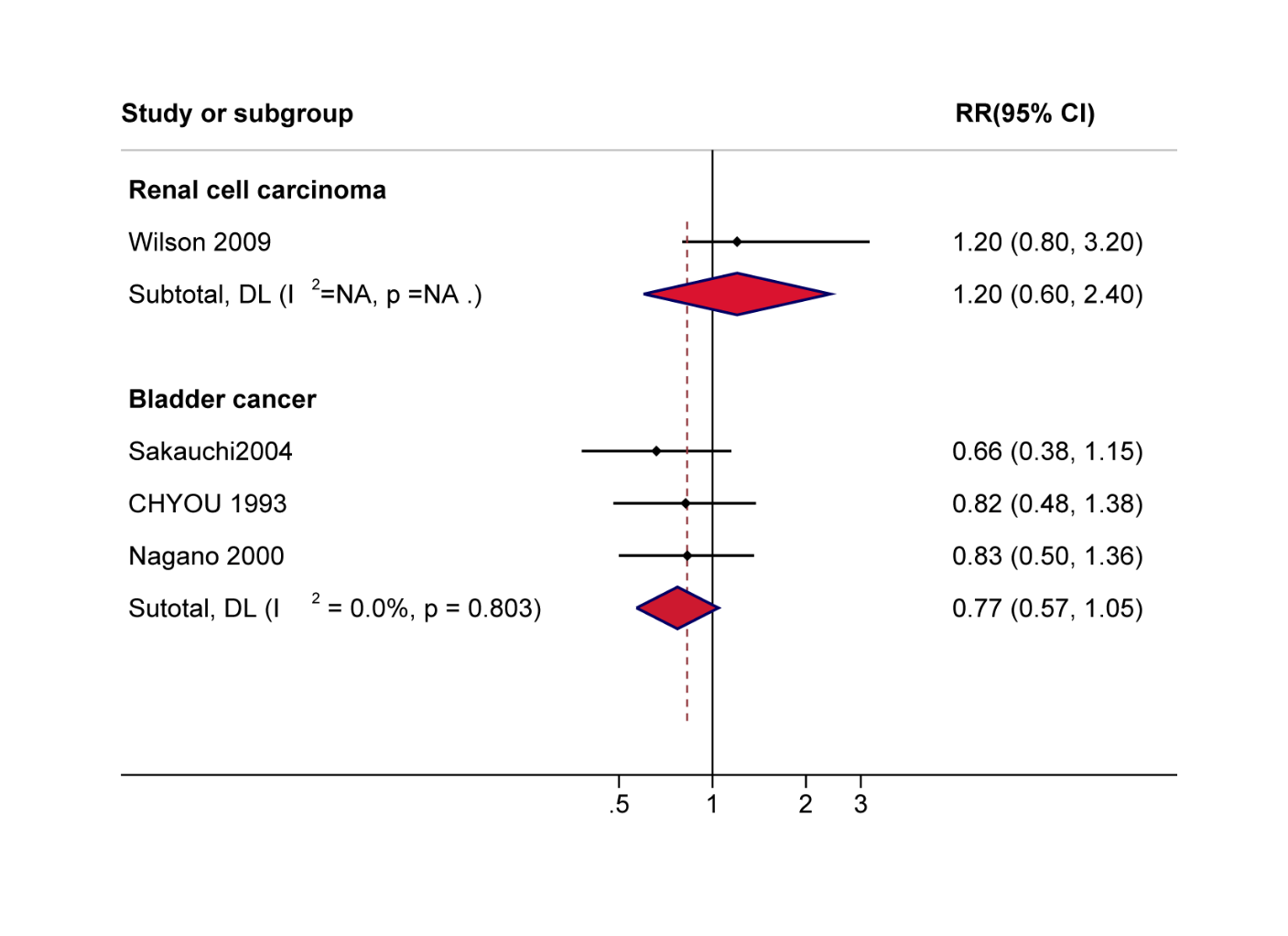


Supplemental Figure 8: Summary of relative risk of bladder cancer, renal cell carcinoma for high versus low egg intake.95%CI,95%confidence interval


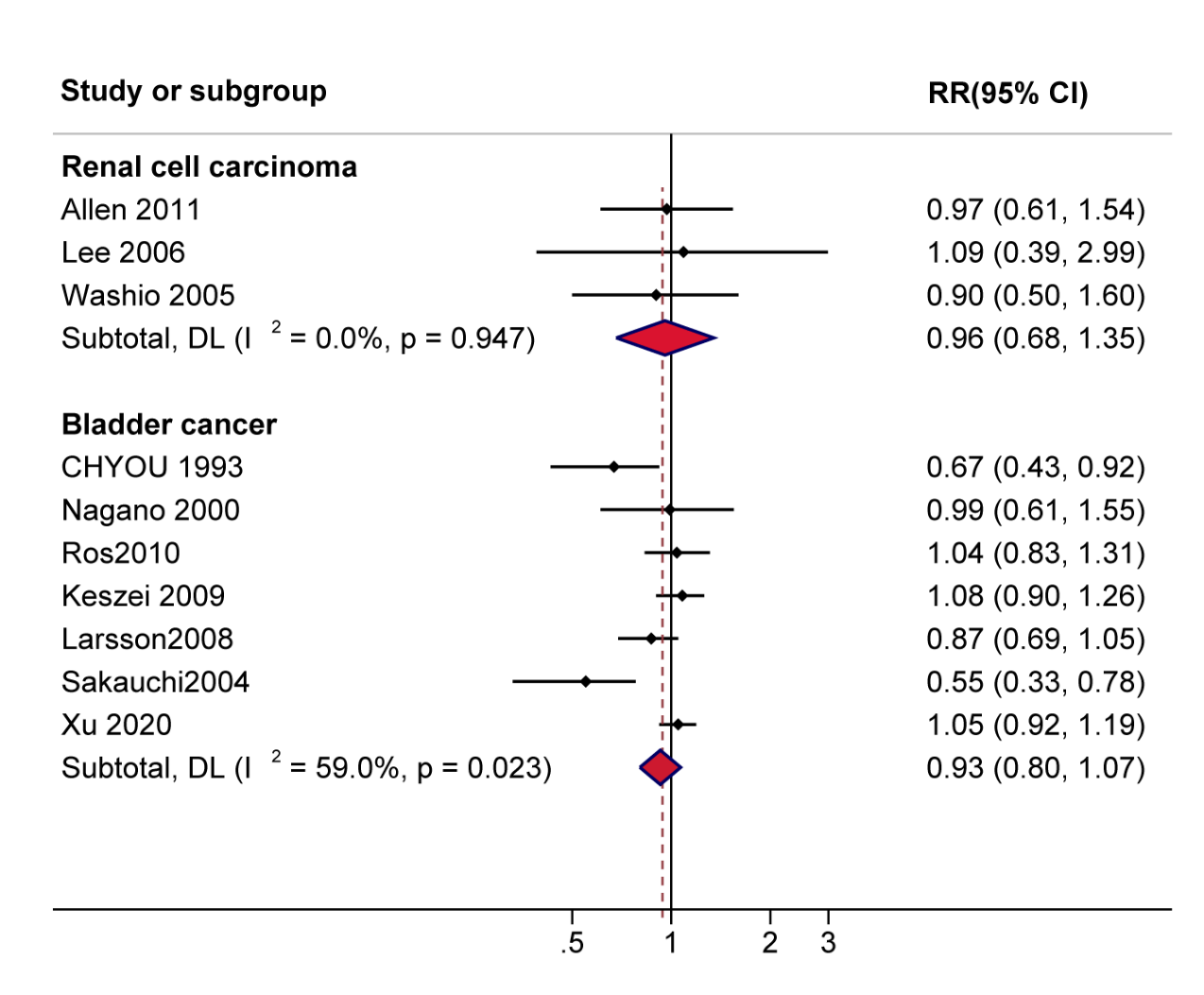


Supplemental Figure 9: Summary of relative risk of bladder cancer, renal cell carcinoma for high versus low dairy intake.95%CI,95%confidence interval


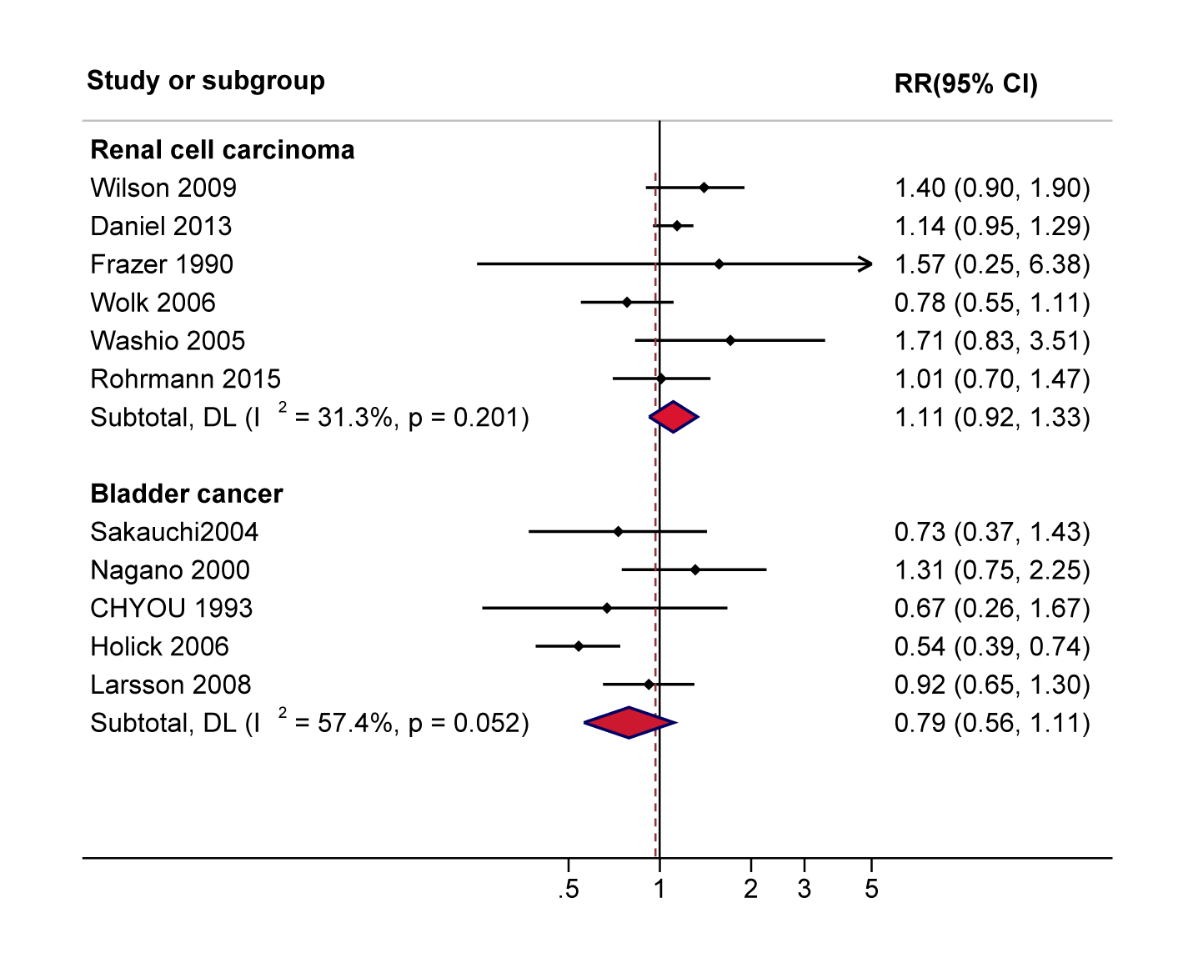


Supplemental Figure 10: Summary of relative risk of bladder cancer, renal cell carcinoma for high versus low fish intake.95%CI,95%confidence interval


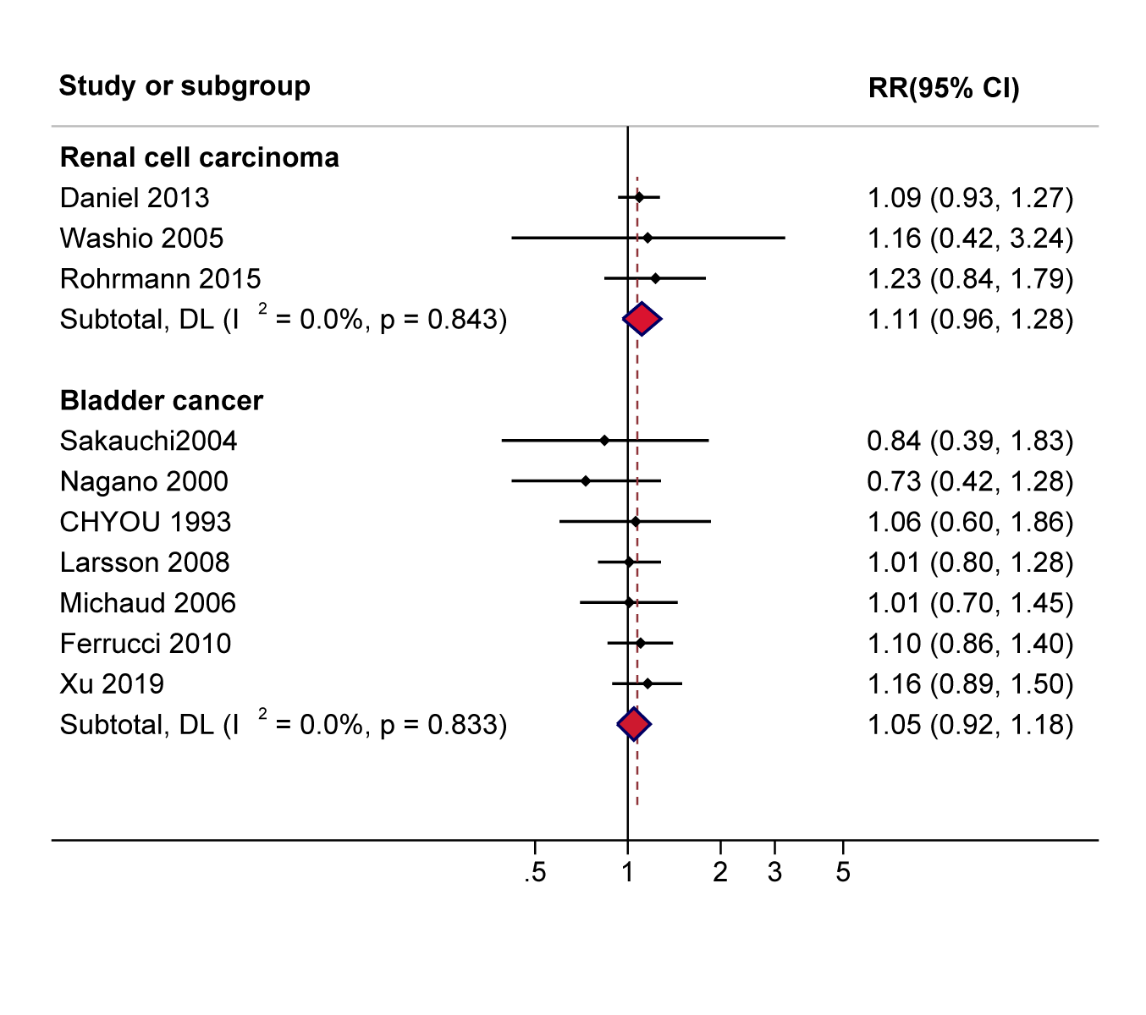


Supplemental Figure 11: Summary of relative risk of bladder cancer, renal cell carcinoma for high versus low processed meat intake.95%CI,95%confidence interval


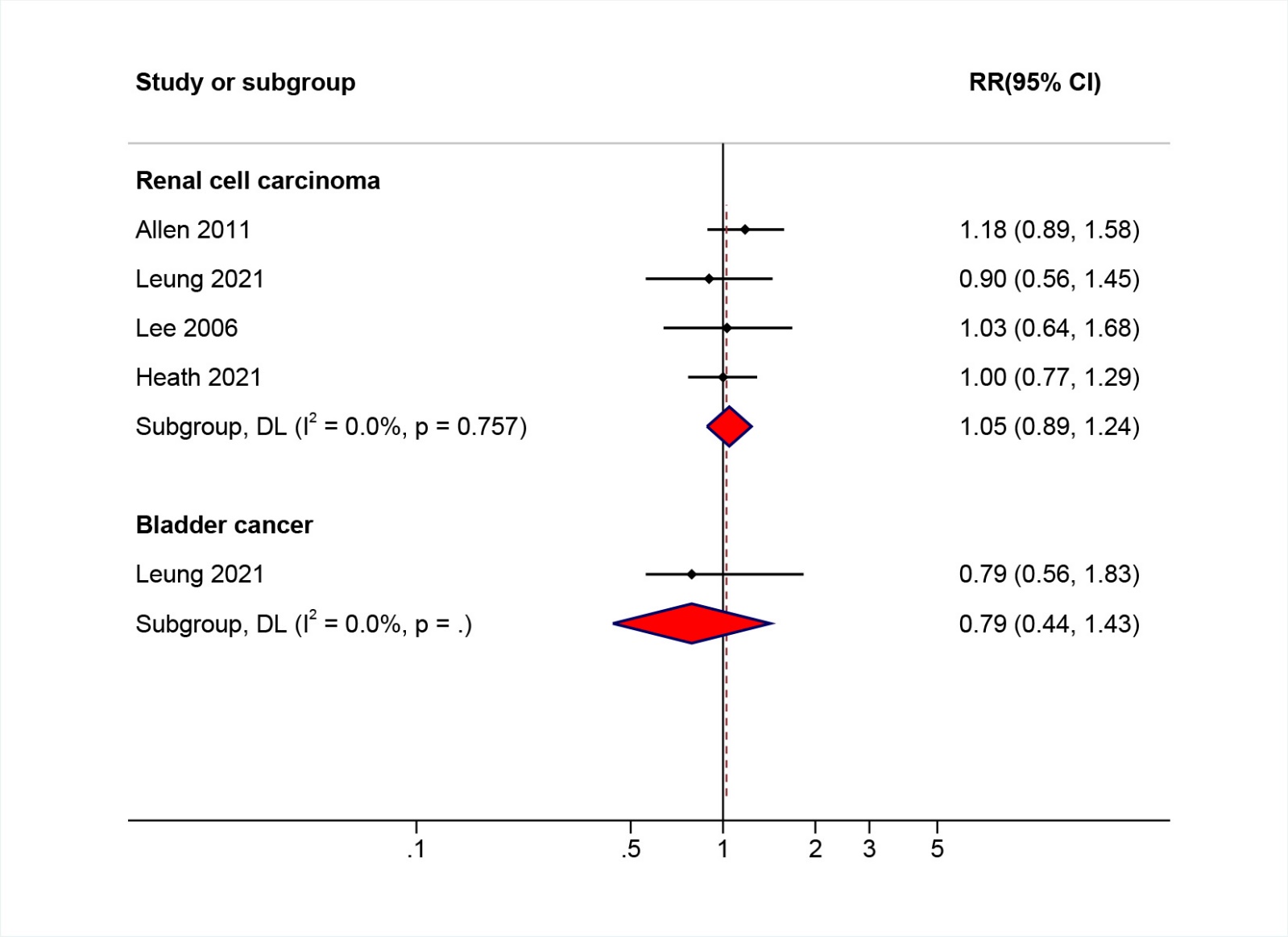


Supplemental Figure 12: Summary of relative risk of bladder cancer, renal cell carcinoma for high versus low sugar sweetened beverage intake.95%CI,95%confidence interval


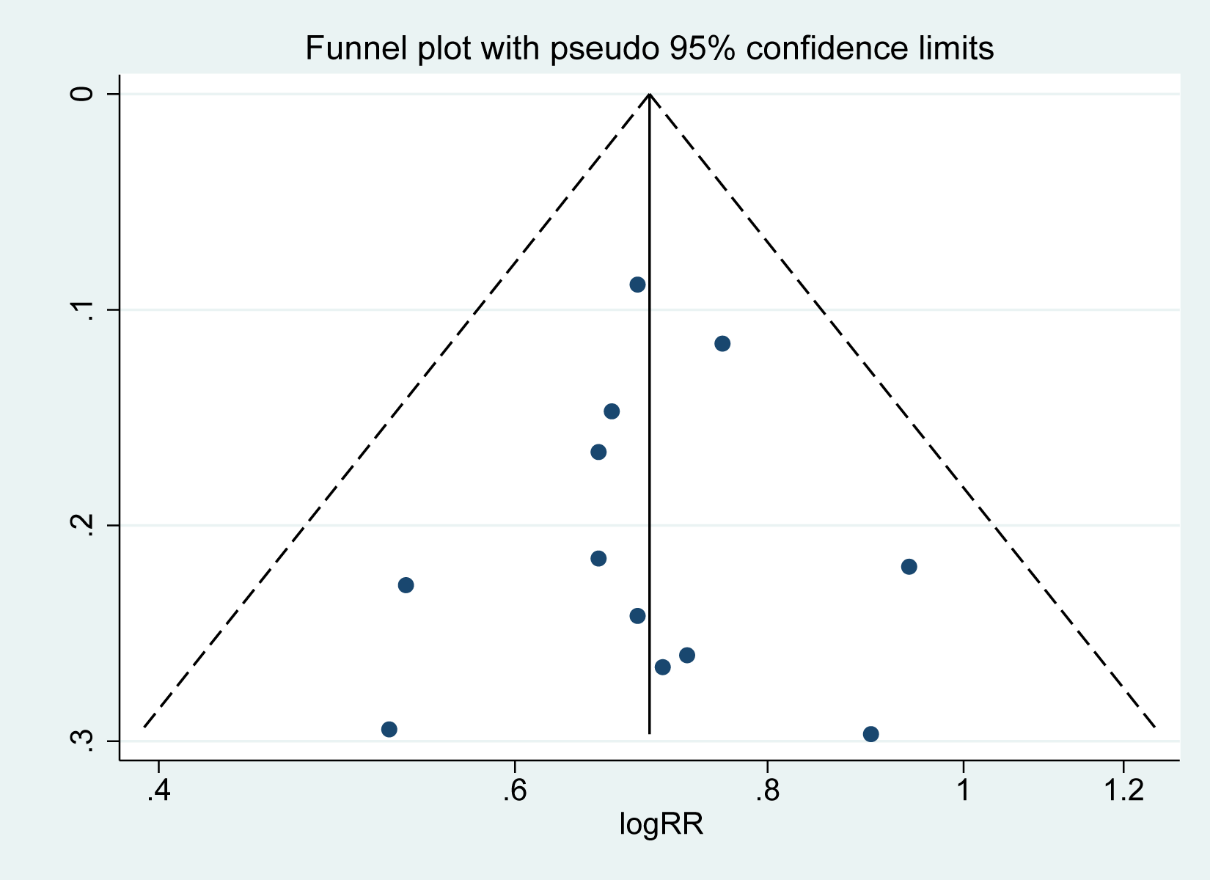


Supplemental Figure 13: Funnel plot for alcohol intake (high vs. low meta-analysis) and renal cell carcinoma. SE =

Standard error


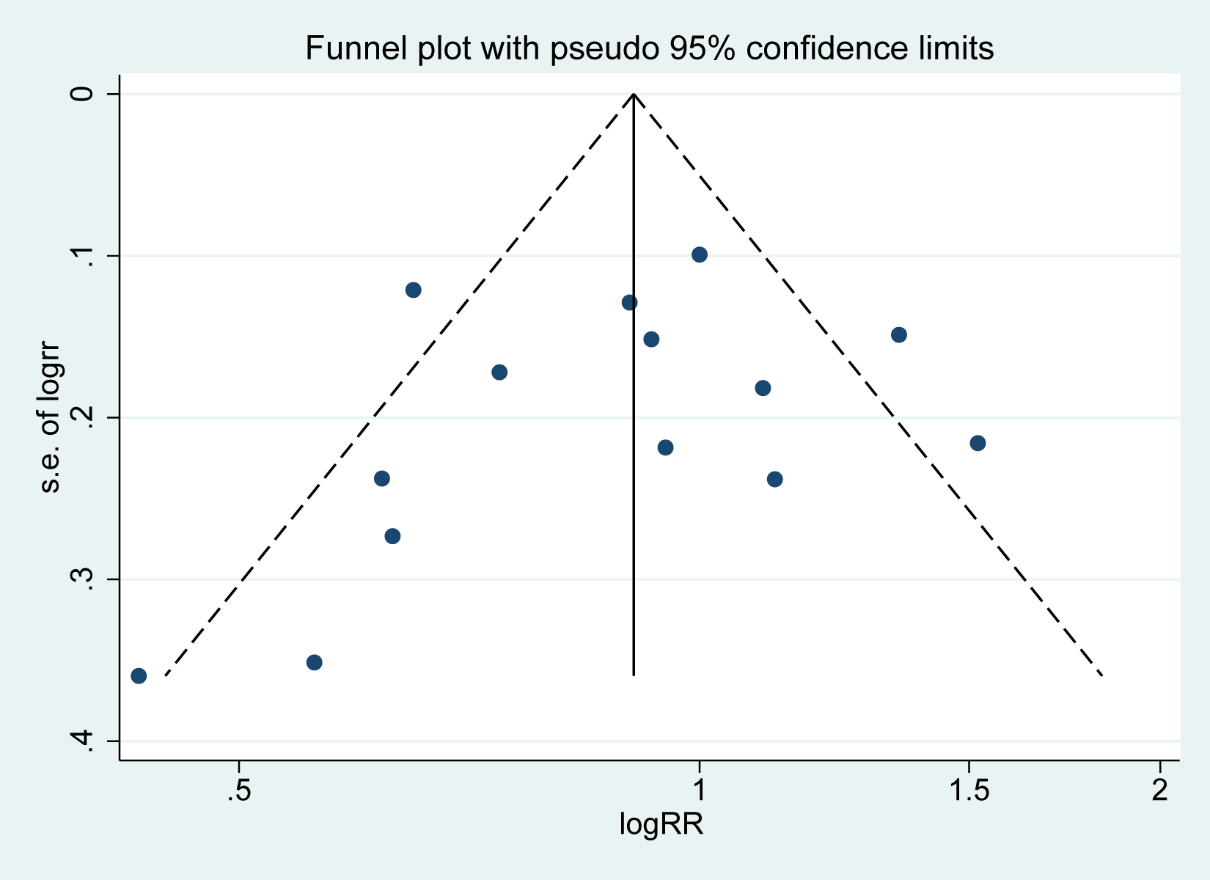


Supplemental Figure 14: Funnel plot for fruit intake (high vs. low meta-analysis) and bladder cancer. SE =

Standard error


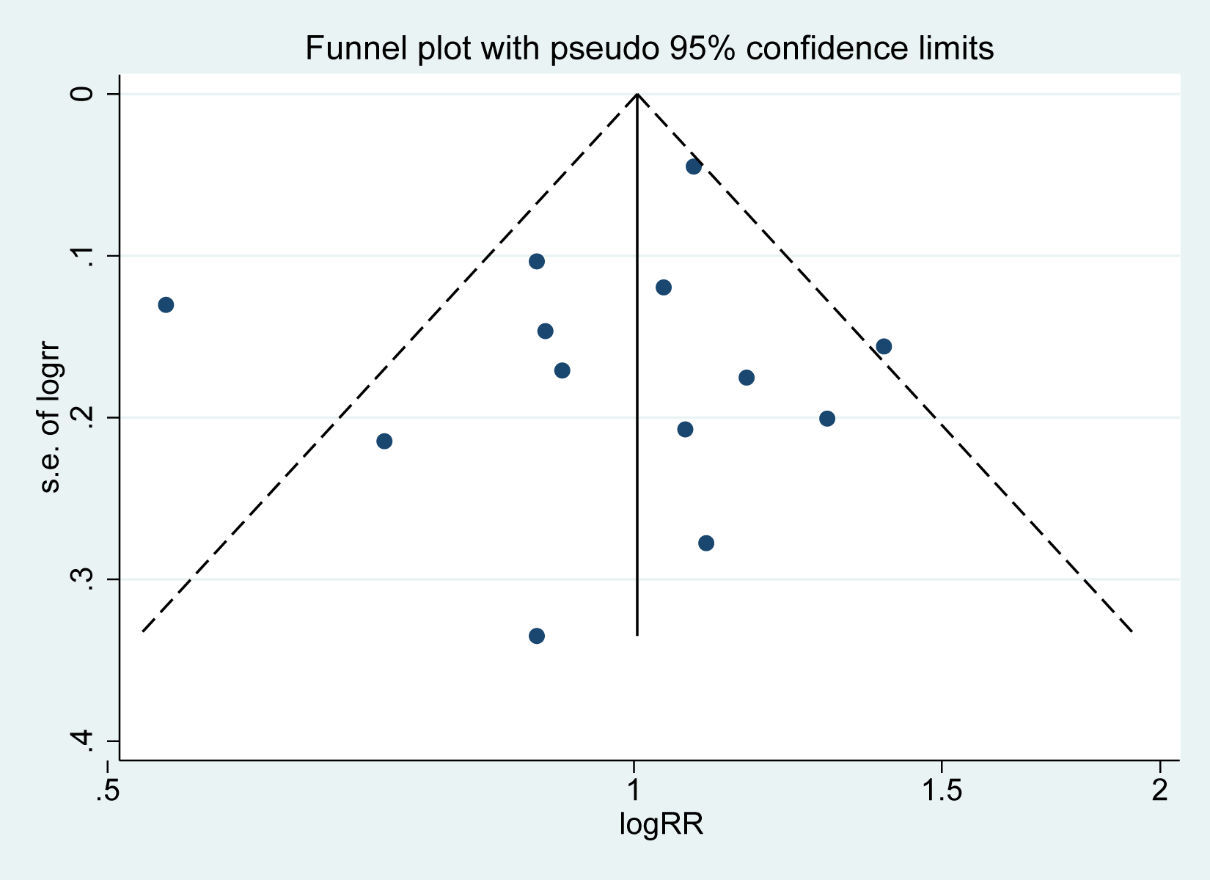


Supplemental Figure 15: Funnel plot for vegetable intake (high vs. low meta-analysis) and bladder cancer. SE =

Standard error


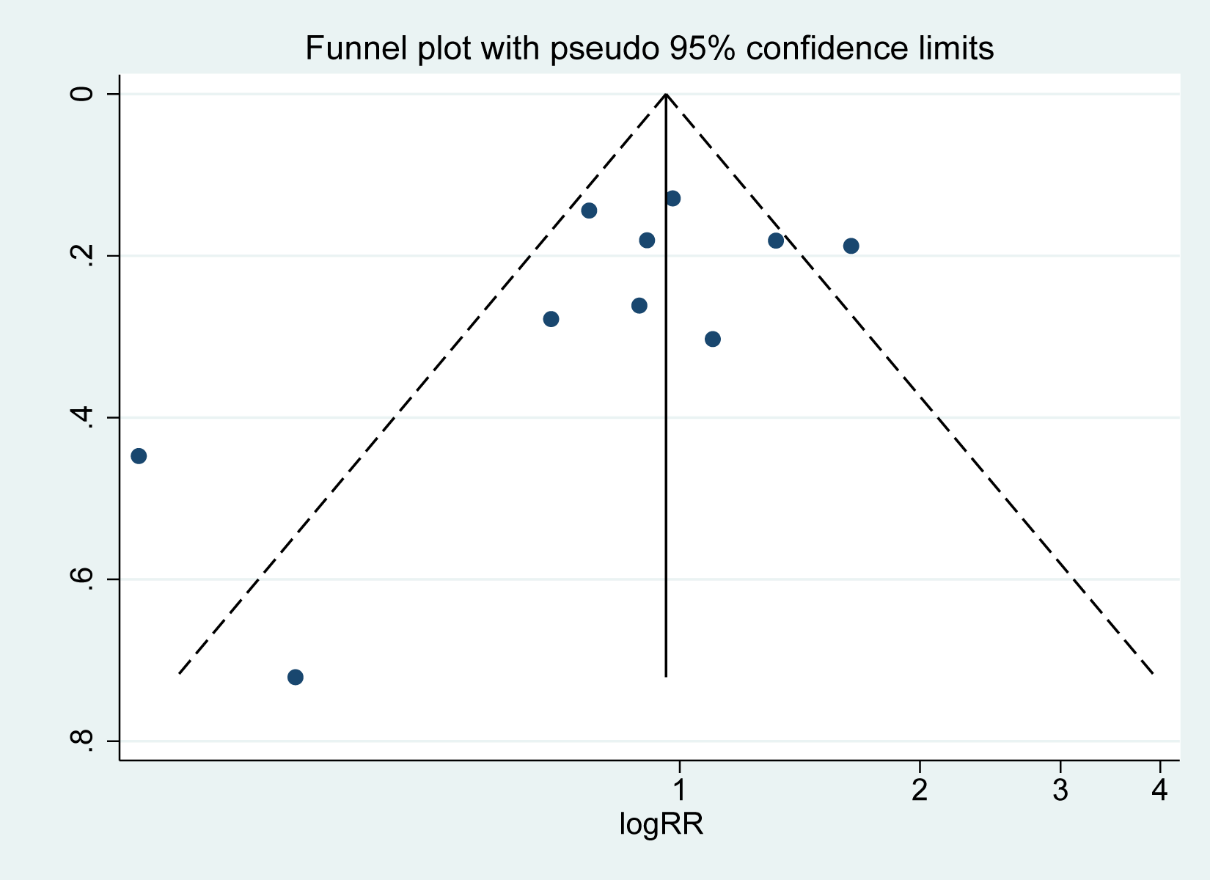


Supplemental Figure 16: Funnel plot for tea intake (high vs. low meta-analysis) and bladder cancer. SE =

Standard error


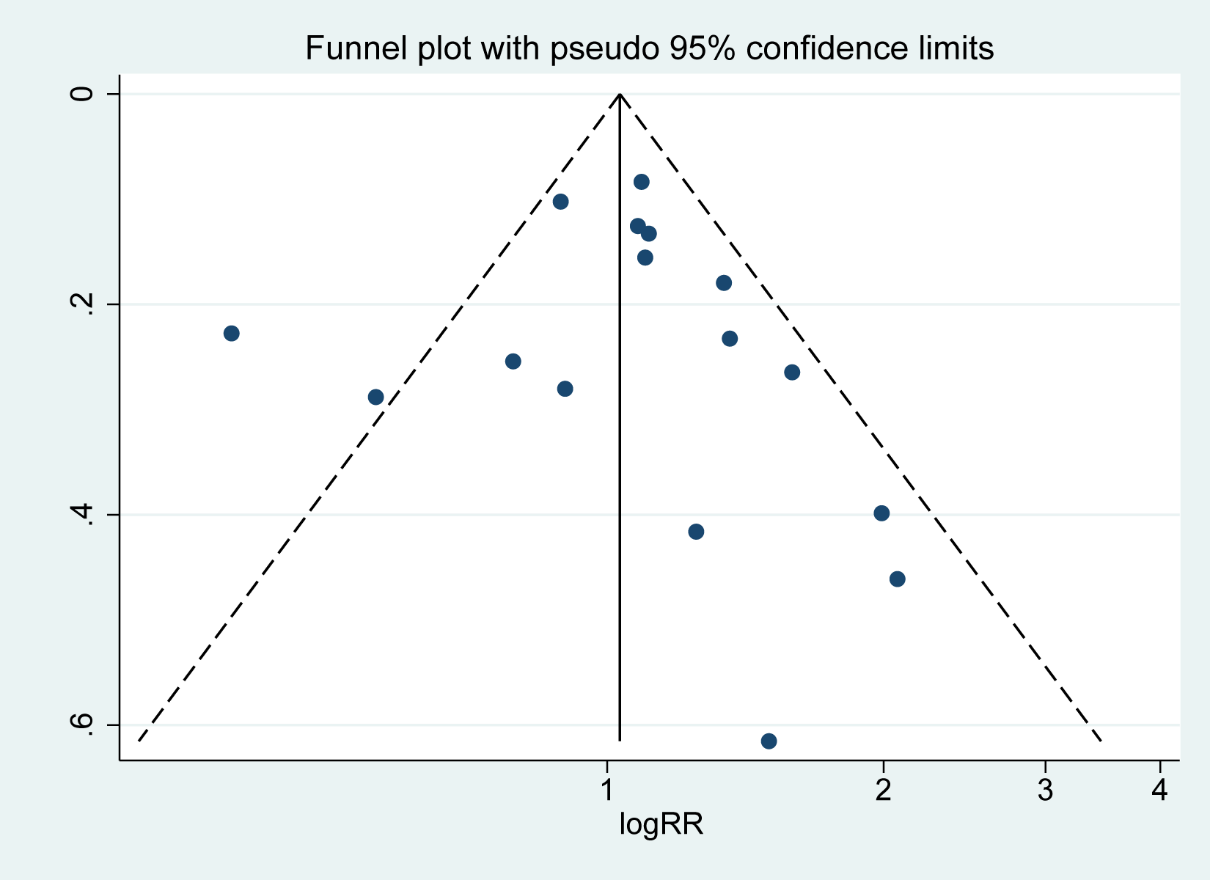


Supplemental Figure 17: Funnel plot for coffee intake (high vs. low meta-analysis) and bladder cancer. SE =

Standard error


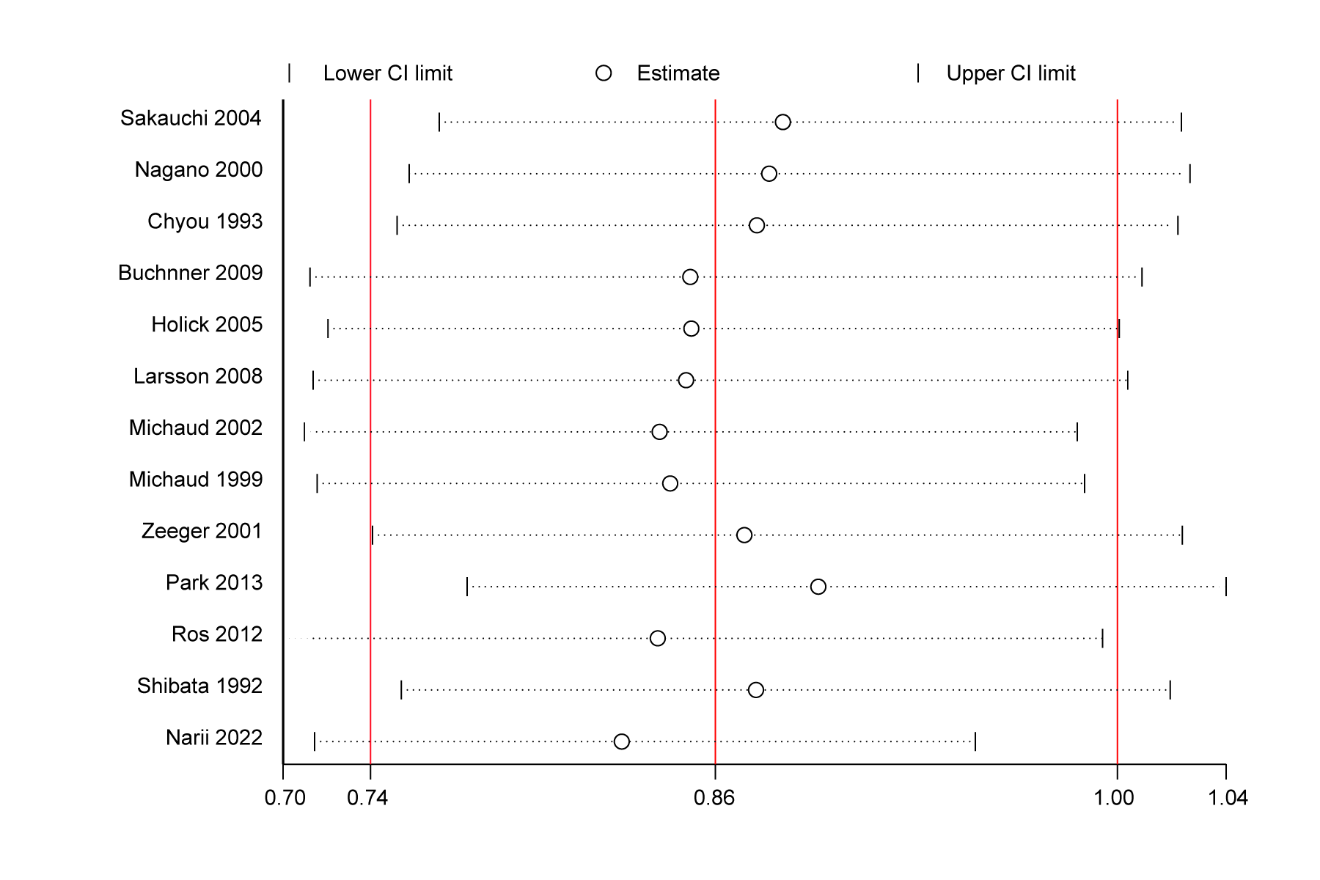


Supplemental Figure 18: Sensitivity analysis for fruit intake (high vs. low meta-analysis) and bladder cancer.


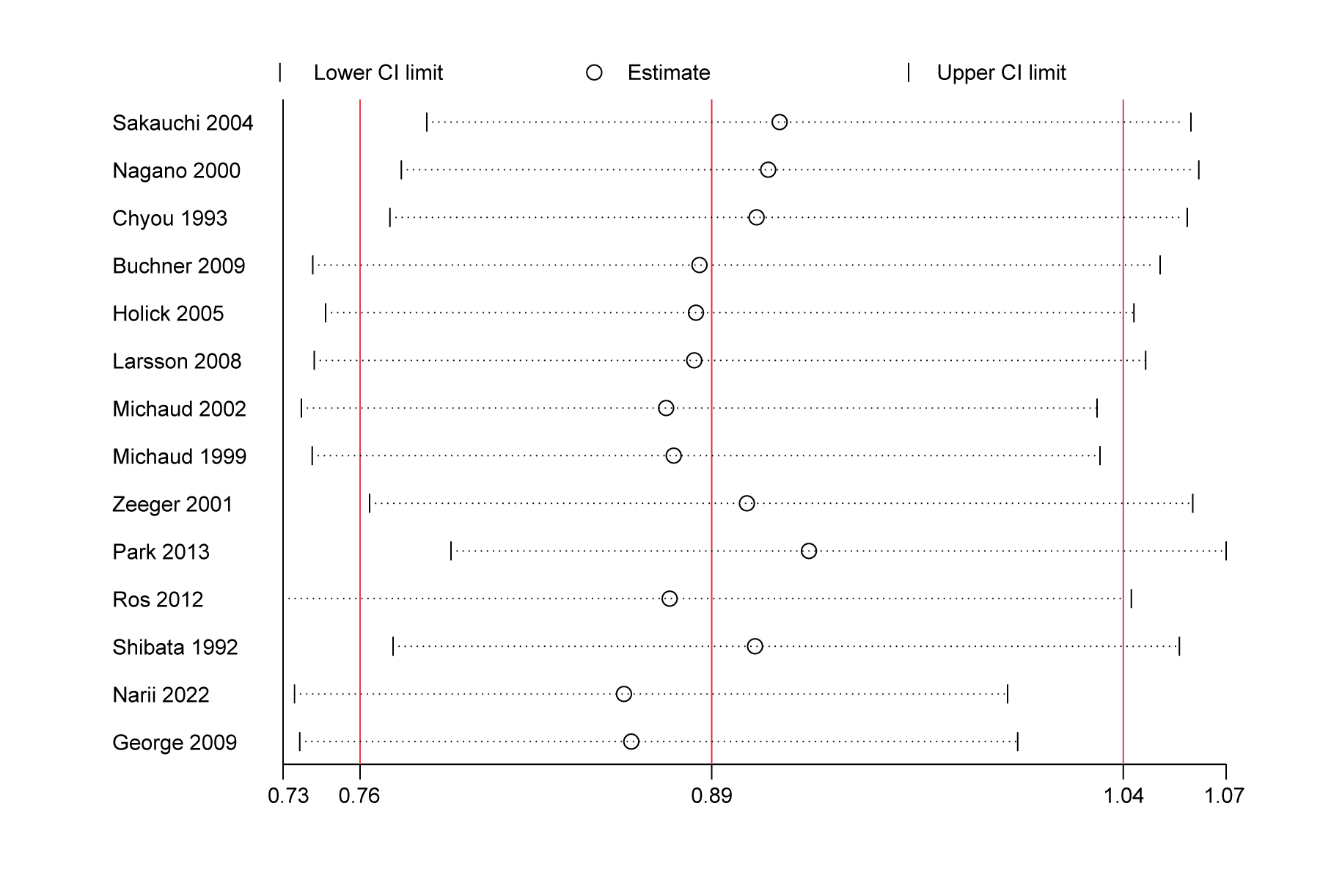


Supplemental Figure 19: Sensitivity analysis for vegetable intake (high vs. low meta-analysis) and bladder cancer.


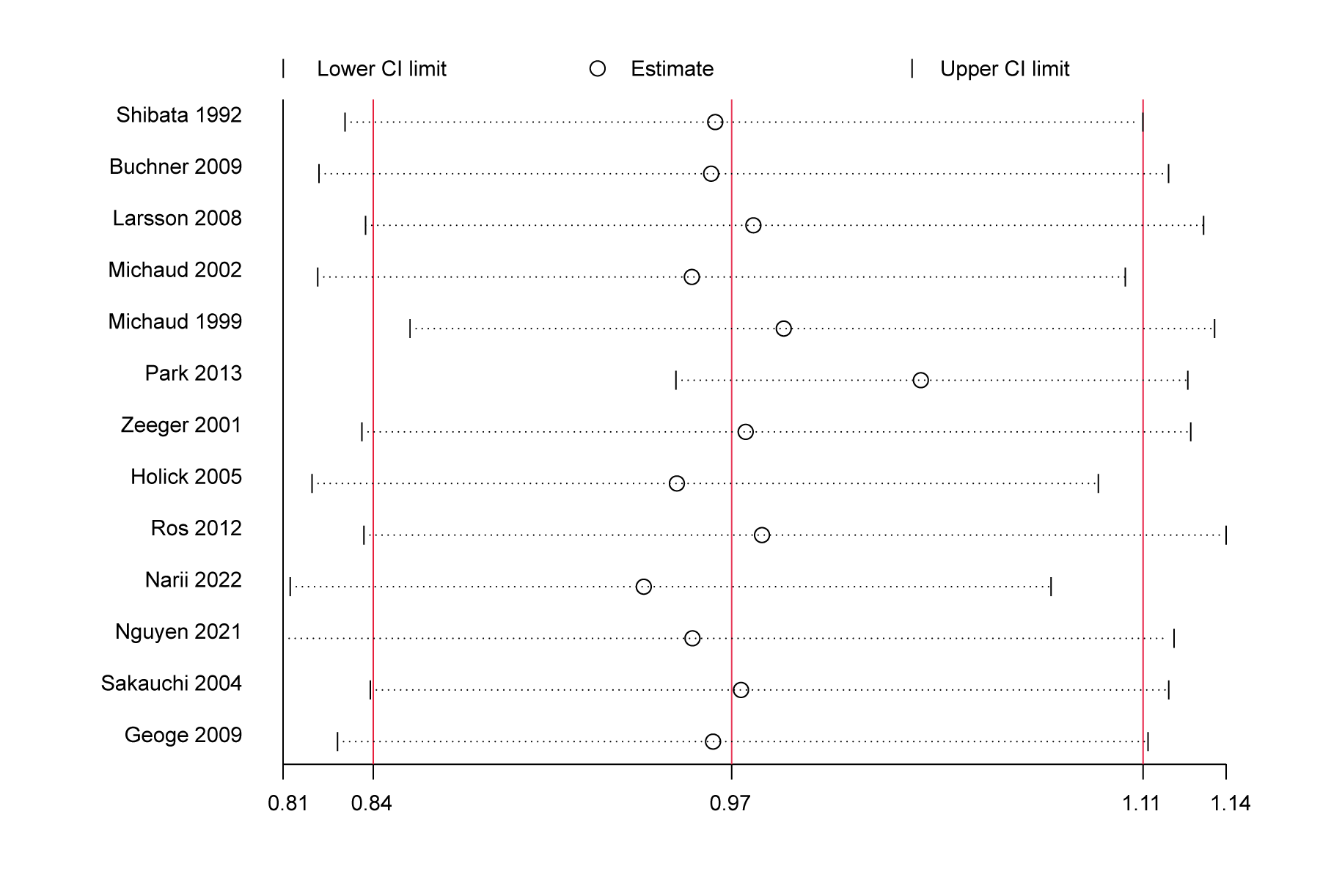


Supplemental Figure 20: Sensitivity analysis for legume intake (high vs. low meta-analysis) and bladder cancer.


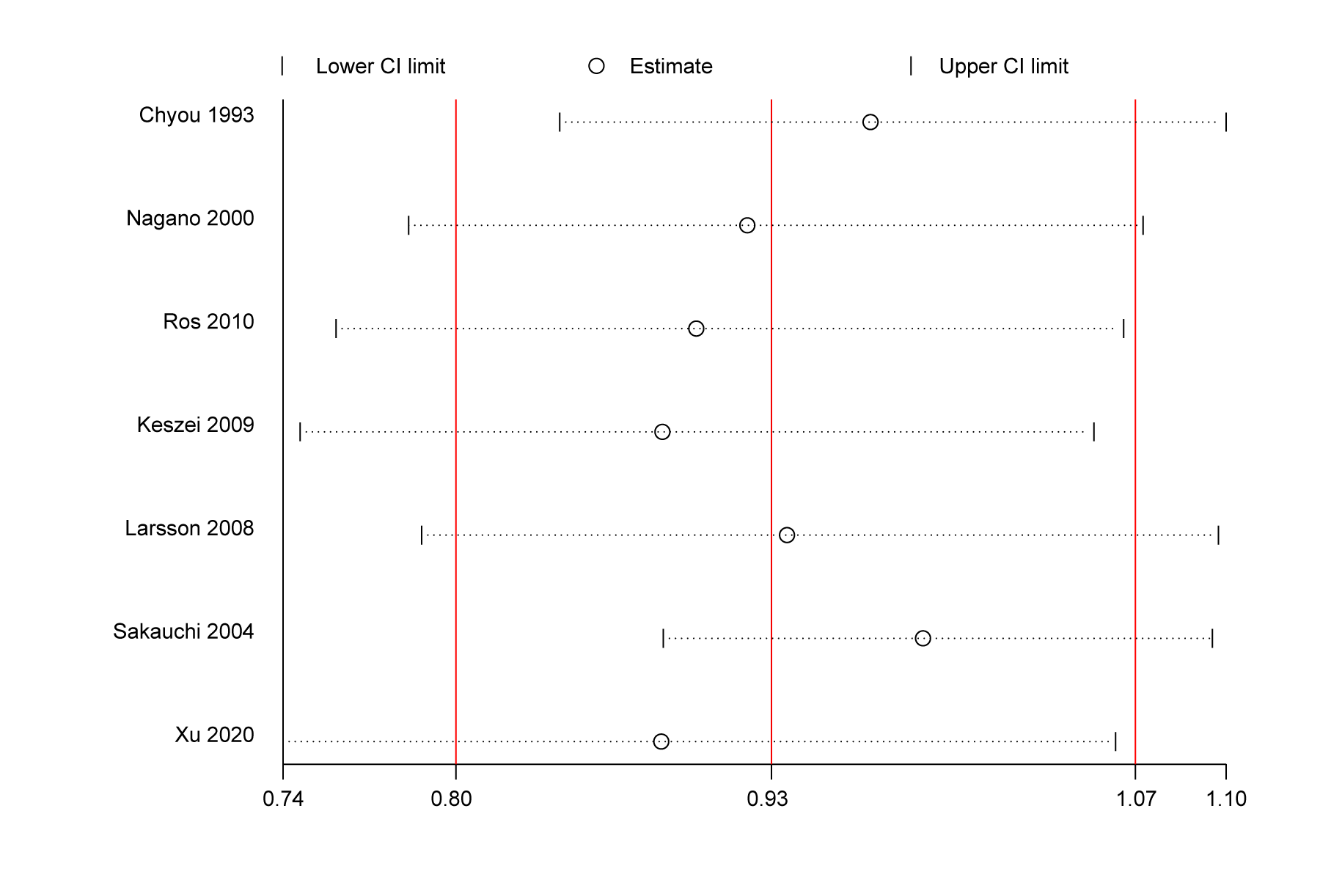


Supplemental Figure 21: Sensitivity analysis for dairy intake (high vs. low meta-analysis) and bladder cancer.


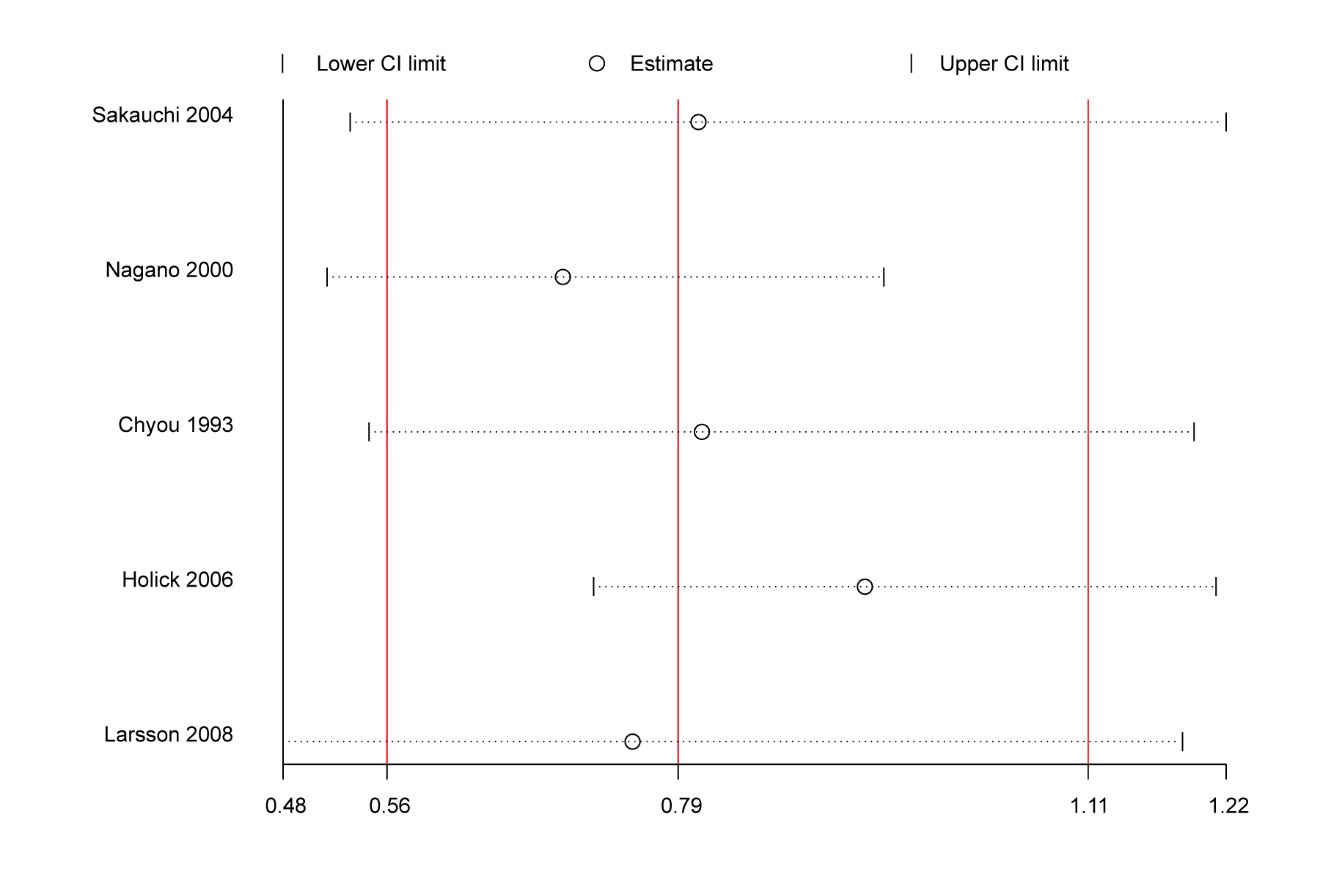


Supplemental Figure 22: Sensitivity analysis for fish intake (high vs. low meta-analysis) and bladder cancer.


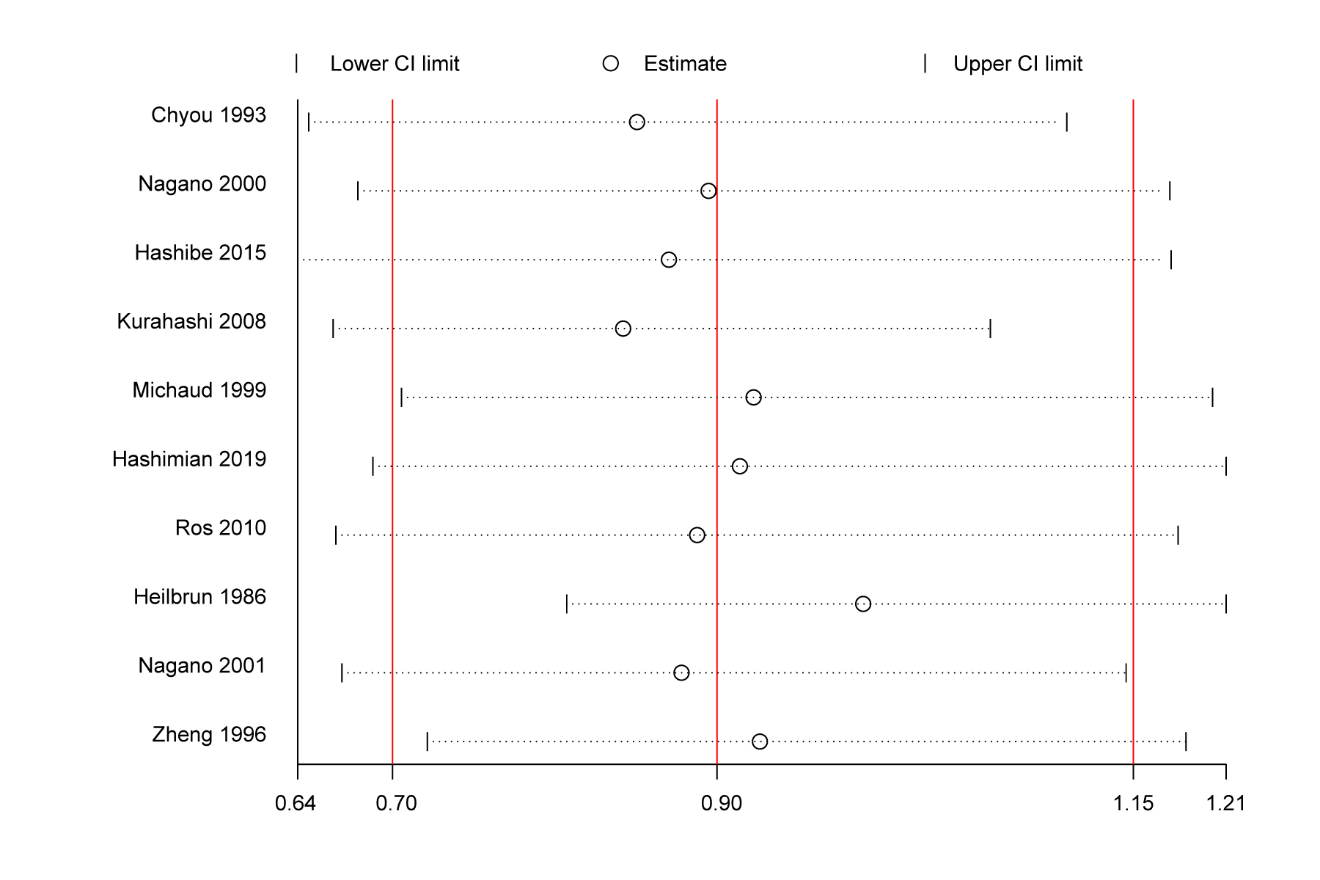


Supplemental Figure 23: Sensitivity analysis for tea intake (high vs. low meta-analysis) and bladder cancer.


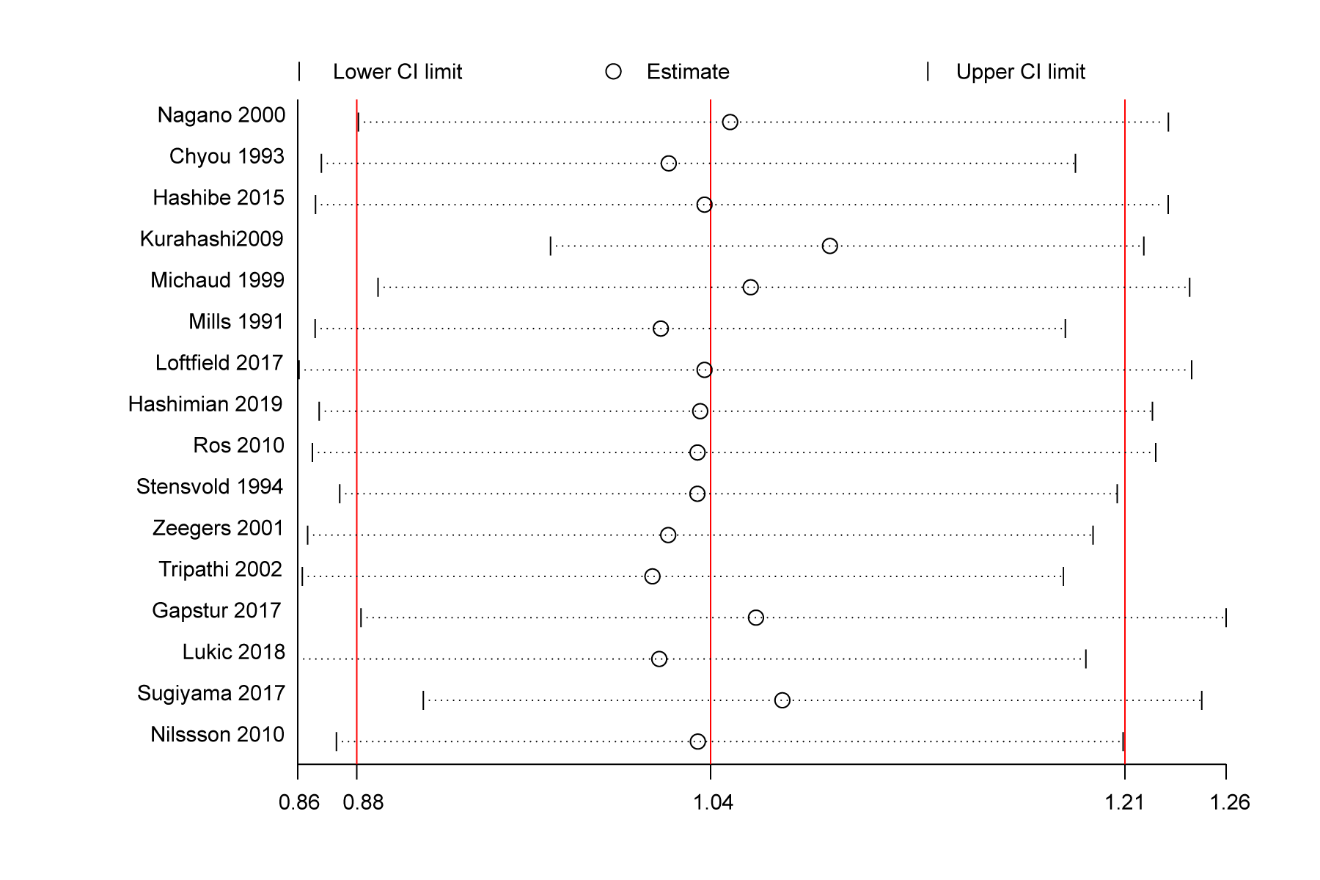
Supplemental Figure 24: Sensitivity analysis for coffee intake (high vs. low meta-analysis) and bladder cancer

1. World Cancer Research Fund International: Continuous Update Project (CUP). London (2017). http://www.wcrf.org/int/research-we-fund/continuous-update-project-cup [↑](#footnote-ref-1)
